# DYRK1A reinforces epithelial-mesenchymal transition and metastasis of hepatocellular carcinoma via cooperatively activating STAT3 and SMAD

**DOI:** 10.1186/s12929-022-00817-y

**Published:** 2022-06-02

**Authors:** Yang-ling Li, Man-man Zhang, Lin-wen Wu, Ye-han Liu, Zuo-yan Zhang, Ling-hui Zeng, Neng-ming Lin, Chong Zhang

**Affiliations:** 1 Department of Clinical Pharmacology, Key Laboratory of Clinical Cancer Pharmacology and Toxicology Research of Zhejiang Province, Affiliated Hangzhou First People’s Hospital, Zhejiang University School of Medicine, No.261 Huansha Road, Hangzhou, 310006 Zhejiang China; 2 School of Medicine, Zhejiang University City College, No.51 Huzhou Street, Hangzhou, 310015 Zhejiang China; 3 College of Pharmaceutical Sciences, Zhejiang University, Hangzhou, 310058 Zhejiang China; 4 Key Laboratory of Clinical Cancer Pharmacology and Toxicology Research of Zhejiang Province, Affiliated Hangzhou First People’s Hospital, Zhejiang University School of Medicine, Hangzhou, 310006 Zhejiang China; 5 Westlake Laboratory of Life Sciences and Biomedicine of Zhejiang Province, Hangzhou, 310024 China

**Keywords:** Hepatocellular carcinoma, DYRK1A, EMT, Metastasis, TSC1

## Abstract

**Background:**

Hepatocellular carcinoma (HCC) accounts for the majority of liver cancer cases, while metastasis is considered the leading cause of HCC-related death. However, the currently available treatment strategies for efficient suppression of metastasis are limited. Therefore, novel therapeutic targets to inhibit metastasis and effectively treat HCC are urgently required.

**Methods:**

Wound healing and Transwell assays were used to determine the migration and invasion abilities of HCC cells in vitro. Quantitative real-time PCR (qRT-PCR), protein array, immunofluorescence, and immunoprecipitation experiments were used to study the mechanism of DYRK1A-mediated metastasis. A tail vein metastasis model and H&E staining were utilized to assess metastatic potential in vivo.

**Results:**

The results of the current study demonstrated that dual-specificity tyrosine phosphorylation-regulated kinase 1A (DYRK1A) was upregulated in HCC tissues compared with normal liver tissues. Additionally, the level of DYRK1A was increased in primary HCC tissues of patients with metastasis compared with those of patients without metastasis, and DYRK1A overexpression correlated with worse outcomes in liver cancer patients. Gain- and loss-of-function studies suggested that DYRK1A enhanced the invasion and migration abilities of HCC cells by promoting epithelial-mesenchymal transition (EMT). Regarding the promoting effect of DYRK1A on cell invasion, the results showed that DYRK1A was coexpressed with TGF-β/SMAD and STAT3 signalling components in clinical tumour samples obtained from patients with HCC. DYRK1A also activated TGF-β/SMAD signalling by interacting with tuberous sclerosis 1 (TSC1) and enhanced metastasis of HCC cells by activating STAT3. Furthermore, DYRK1A promoted EMT by cooperatively activating STAT3/SMAD signalling.

**Conclusion:**

Overall, the present study not only uncovered the promoting effect of DYRK1A on HCC metastasis and revealed the mechanism but also provided a new approach to predict and treat metastatic HCC.

**Supplementary Information:**

The online version contains supplementary material available at 10.1186/s12929-022-00817-y.

## Background

The high mortality rate of liver cancer is primarily due to metastasis and postsurgical recurrence [1]. The majority of patients with hepatocellular carcinoma (HCC), the most common type of liver cancer, are diagnosed at an advanced stage of the disease with distant metastases, when the available treatment options are limited [2, 3]. Therefore, identifying key molecules involved in HCC progression is of great importance for the development of novel therapeutic strategies. Epithelial-mesenchymal transition (EMT) is a key step in the early stages of cancer metastasis [4]. The Janus kinase (JAK)/signal transducer and activator of transcription 3 (STAT3) and TGF-β1-Smad2/3 signalling pathways, important cascades for signal transduction from plasma membrane receptors to the nucleus, synergistically augment EMT and promote metastasis [5]. These two signalling pathways participate in crosstalk during EMT in HCC: STAT3 regulates Smad3-mediated signalling induced by TGF-β, while Smad3 regulates STAT3-mediated signalling induced by various cytokines [6]. Thus, it is critical to develop novel therapeutic molecules to target these two signalling pathways linked to EMT and metastasis in HCC.

Dual-specificity tyrosine phosphorylation-regulated kinase 1A (DYRK1A), located in the Down’s syndrome critical region, plays pivotal roles in neurodegenerative diseases, such as Alzheimer’s disease and Down’s syndrome [7]. Furthermore, it has been suggested that targeting DYRK1A is a potential strategy for treating cancer [8]. For instance, a study revealed that DYRK1A can modulate the expression of c-mesenchymal-epithelial transition receptor (c-MET) and drive tumour growth, and it was therefore considered a target for treating pancreatic ductal adenocarcinoma [9]. In addition to mediating tumour growth, DYRK1A is also involved in the regulation of several cellular processes associated with cancer progression, such as cancer stemness maintenance and cancer cell invasion [10].

Tumour metastasis, the major cause of mortality in patients with cancer, is a multistep process that involves tumour cell invasion through the basal membrane into blood vessels followed by extravasation and colonization of host organs [11, 12]. Angiogenesis represents the process by which new blood capillaries are formed from preexisting vasculature, thus promoting primary tumour growth and metastasis [13]. A study demonstrated that DYRK1A positively regulated VEGF-dependent nuclear factor of activated T cells (NFAT) transcriptional responses in endothelial cells and was involved in angiogenesis [14]. In addition, DYRK1A enhanced the migration ability of glioblastoma cells by activating NFATC1 in vitro [15]. Although current evidence has highlighted the importance of DYRK1A in tumour metastasis, its promoting effects on tumour metastasis, as well as the underlying mechanism, need further investigation. Furthermore, EMT is involved in metastatic dissemination of carcinomas [16]. The present study first demonstrated that DYRK1A can promote HCC cell EMT and metastasis both in vitro and in vivo.

## Materials and methods

Transforming growth factor-β (TGF-β; catalogue number: 100–21) was obtained from PeproTech (Rocky Hill, NJ, USA). We obtained harmine (catalogue number: HY-N0737A) from MedChemExpress (Monmouth Junction, NJ, USA). Anti-N-cadherin (14215S), anti-E-cadherin (3195P), anti-DYRK1A (2771), anti-p-Smad3 (9520), anti-Slug (9585) and anti-Snail (3879S) antibodies were obtained from Cell Signaling Technology (Danvers, MA, USA). Anti-β-actin (47778), anti-Smad2/3 (133098), and anti-STAT3 (482) antibodies were purchased from Santa Cruz Biotechnology (Dallas, Texas, USA). The anti-p-STAT3 (Tyr705) (ab76315) and anti-tuberous sclerosis 1 (TSC1; ab270967) antibody was obtained from Abcam (Cambridge, Cambridgeshire, UK). The anti-α-tubulin (AF0001) antibody was purchased form Beyotime Institute of Biotechnology (Shanghai, China).

### Cell culture

HepG2 cells and Hep3B cells were purchased from Shanghai Institute of Biochemistry and Cell Biology and cultured in Dulbecco’s modified Eagle medium (DMEM) containing 10% foetal bovine serum (FBS). The human foetal hepatocyte line L-02 was cultured in Dulbecco’s modified Eagle’s medium (DMEM) containing 10% FBS.

### Wound healing assay

Treated HCC cells (8 × 10^5^ cells/well) were incubated in 24-well plates. Following 24 h of incubation, the cells were pretreated with 5 μM mitomycin C for 3 h to suppress proliferation. Subsequently, the HCC cell monolayer was scratched using a sterile 10-µL pipette tip and was then washed with PBS. After 24 h, images of the migrated cells were acquired under a microscope. The migrating cells in five distinct scratched areas per well were counted, and the migration rate was calculated.

### Transwell migration and invasion assays

Briefly, following serum starvation overnight, HCC cells (1 × 10^5^ cells/mL) in serum-free medium were seeded in the Transwell chambers, with or without a Matrigel coating on the insert membrane, for the invasion or migration assay, respectively. Then, 600 μL of DMEM supplemented with 20% FBS was added to the lower compartments of the Transwell chambers. Following incubation at indicated times, cells on the bottom surface of the membrane were fixed and stained with crystal violet solution containing 20% methanol for 30 min. Quantification of migrated and invaded cells was performed with ImageJ software.

### Transfection and DYRK1A short hairpin RNA (shRNA) construction

HCC cells were incubated in six-well plates (2 × 10^5^ cells/well) overnight, and transfection of siRNA or plasmids was then performed with jetPRIME (Polyplus, NY, USA) following the manufacturers’ protocols. The sense sequences of the DYRK1A siRNAs were 5′-AUGGAGCUAUGGACGUUAATT-3′ (DYRK1A siRNA-1) and 5′-AAACUCGAAUUCAACCUUATT-3′ (DYRK1A siRNA-2), that of the STAT3 siRNA was 5′- CCACUUUGGUGUUUCAUAATT-3′, that of the TSC-1 siRNA was 5′-CGGCUGAUGUUGUUAAAUATT-3′, and that of the negative control siRNA was 5′-UUCUCCGAACGUGUCACGUTT-3′.

The following oligonucleotide sequence (forward primer) was used to synthesize the DYRK1A shRNA constructs (the targeted sequence is bolded). 5′-CCGG**GTTCGGCTTGCACCGTCATTT**CTCGAGAAATGACGGTGCAAGCCGAACTTTTTG-3′. The reverse primer sequence was 5′-AATTCAAAAA**GTTCGGCTTGCACCGTCATTT**CTCGAGAAATGACGGTGCAAGCCGAAC-3′. The designed shRNA contained 58 bp with 21 bp of perfectly matched nucleotide base pairs connected by a 6 bp loop [CTCGAG]. The shRNA targeting DYRK1A was synthesized and inserted into the pLKO.1-TRC Cloning vector empty vector according to the manufacturer’s instructions. The DYRK1A shRNA and NC shRNA were transfected into HepG2 cells using Lipofectamine 3000 reagent (Invitrogen, Shanghai, China) according to the manufacturer’s instructions. After transfection, HepG2 cells were selected using puromycin (10 μg/ml) for 5 days. Puromycin-resistant colonies were then single-cell cloned and expanded. The expression of DYRK1A was determined by western blot analysis.

### Western blot

HCC cells were first lysed in RIPA buffer, and samples containing ~ 20 μg of protein were then separated by SDS–PAGE and transferred to a PVDF membrane. Subsequently, the membrane was blocked using 5% (w/v) skim milk, incubated with primary antibodies, and then incubated with the corresponding secondary antibodies. Finally, the blots were visualized using an imaging system.

### Immunofluorescence analysis

For immunofluorescence analysis, treated HCC cells were incubated in a culture dish overnight at 37 °C. Following incubation, HCC cells were incubated with 4% paraformaldehyde for 15 min and permeabilized using 0.3% Triton X-100 for 30 min. After blocking using 1% BSA for 30 min, the cells were incubated with specific antibodies at 4 °C for 24 h and were then stained with the corresponding isotype-specific secondary antibodies for 1 h in the dark. Nuclei were stained with 0.1% DAPI solution, and staining was analysed using a fluorescence microscope.

### qRT–PCR

Total RNA was isolated with TRIzol, sedimented with isopropyl alcohol, washed with 70% ethanol two times, and reverse transcribed into cDNA. SYBR Green-based qRT–PCR was used to examine mRNA expression levels. The primers were as follows: DYRK1A, 5′-TCTGGGTATTCCACCTGCTC-3′ (forward) and 5′-GTCCTCCTGTTTCCACTCCA-3′ (reverse); GAPDH, 5′-GAGTCAACGGATTTGGTCGT-3′ (forward) and 5′-TTGATTTTGGAGGGATCTCG-3′ (reverse); N-cadherin, 5′-CAACTTGCCAGAAAACTCCAGG-3′ (forward) and 5′-ATGAAACCGGGCTATCTGCTC-3′ (reverse); E-cadherin, 5′-GAACGCATTGCCACATACAC-3′ (forward) and 5′-GAATTCGGGCTTGTTGTCAT-3′ (reverse).

### Immunoprecipitation

An immunoprecipitation assay was performed to evaluate protein–protein interactions. Briefly, HCC cells were lysed using an NP-40 solution supplemented with protease inhibitor cocktail. After centrifugation at 12,000×*g* for 15 min, the lysates were incubated with 20 μL of protein A/G beads for 1 h. Subsequently, following centrifugation, the supernatants were incubated first with 5 μL of a specific primary antibody for 10 h and then with protein A/G magnetic beads for 2 h. Finally, the beads were analysed using western blotting.

### Tail vein injection model of metastasis in mice

Four- to six-week-old female BALB/c nude mice were purchased from Shanghai SLAC laboratory animal company and randomly divided into two groups (n = 6 mice per group). Subsequently, 1 × 10^6^ HCC cells suspended in 100 μL of PBS were injected into the tail veins of nude mice. Sixty days after injection, the nude mice were sacrificed to evaluate cell metastasis in vivo. Briefly, tissues were embedded in paraffin and were then stained with haematoxylin and eosin (H&E) to evaluate cell metastasis. Lung metastasis was detected by staining with Bouin’s solution and H&E. Neutral balsam was used to preserve the samples. The experiments were performed in compliance with the National Institutes of Health Guide for the Care and Use of Laboratory Animals.

### Statistical analyses

Data are presented as the mean ± standard deviation values. Two‐tailed Student’s t‐test was used to determine significant differences. *p < 0.05; **p < 0.01; ***p < 0.001.

## Results

### DYRK1A plays an essential role in the malignant progression and metastasis of HCC

**Fig. 1:**
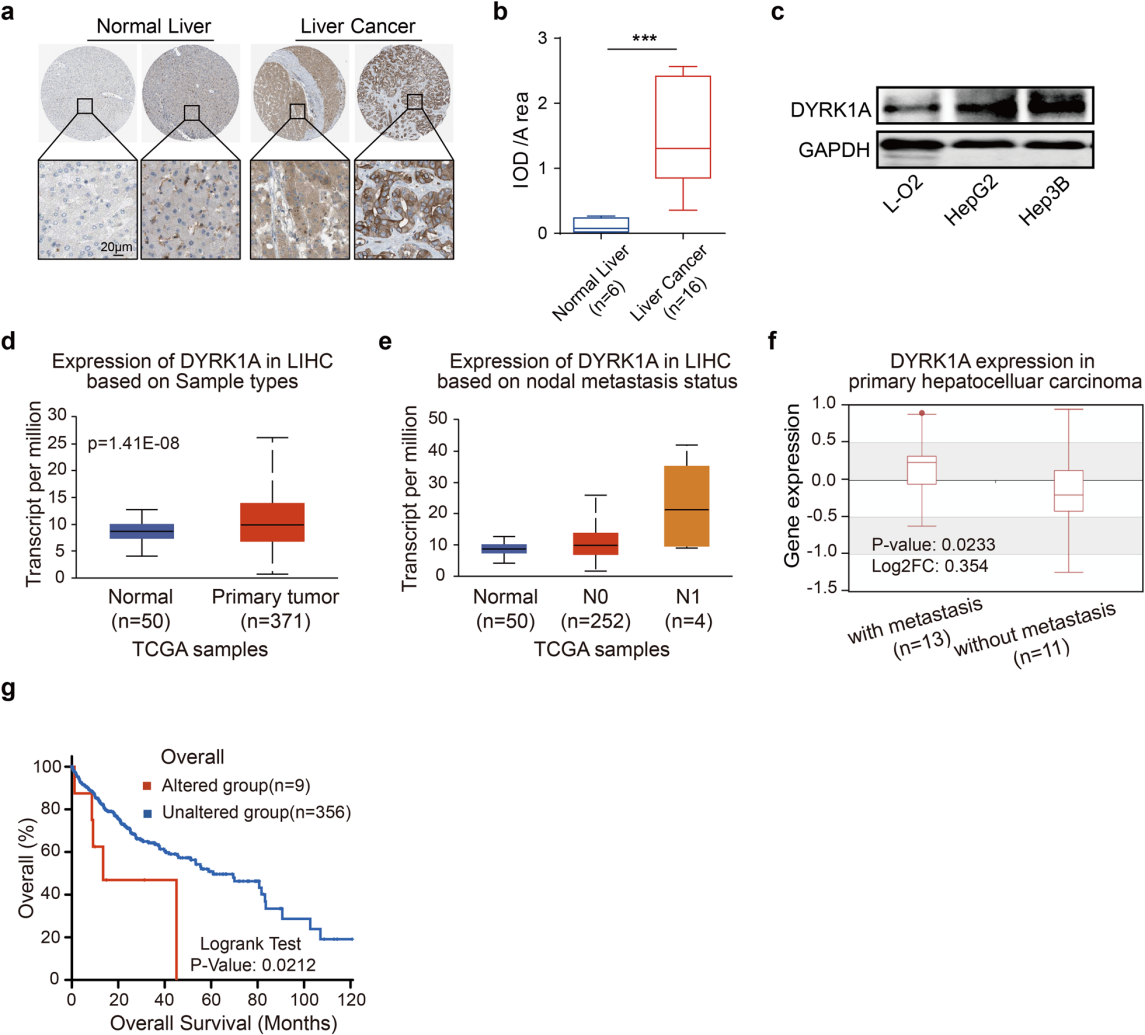
DYRK1A promotes malignant progression and especially metastasis in HCC. **a** and **b** Immunohistochemical staining of DYRK1A in HCC tissues and normal liver tissues was analysed in the Human Protein Atlas (www.proteinatlas.org). Staining intensity was analysed by using Image Pro-Plus and is presented as integrated optical density per stained area (IOD/area). **c** The expression of DYRK1A was detected in liver cancer cells and normal liver cells. **d** and **e** The levels of DYRK1A in HCC and normal liver tissues are shown. Data obtained from UALCAN (ualcan.path.uab.edu/analysis.html) are shown. Gene: DYRK1A; TCGA dataset: Liver HCC; DYRK1A expression based on **d** sample type and **e** nodal metastasis status. The criteria for determining the status of lymph node metastasis in the UALCAN platform were as follows: N0 indicated no regional lymph node metastasis; N1 indicated metastasis to 1–3 axillary lymph nodes. **f** Data acquired from the Human Cancer Metastasis Database (hcmdb.i-sanger.com/index) are shown. Gene: DYRK1A; Exp ID: EXP00156. **g** Data acquired from cBioPortal (www.cbioportal.org) are shown. Gene: DYRK1A; Sample type: Liver HCC (TCGA, Firehose Legacy); Survival type: Overall

To investigate the key genes involved in HCC metastasis, we established a series of HepG2 cells with different invasion abilities by serial selection in a Transwell assay (Additional file 1: Fig. S1a). RNA sequencing was performed to compare the gene transcription levels between highly invasive HepG2 cells (P5) and weakly invasive HepG2 cells (P0), and DYRK1A was found to be overexpressed in P5 compared with P0 HepG2 cells (Additional file 6: Table S1). Furthermore, bioinformatic analysis using the Human Protein Atlas database revealed that DYRK1A was upregulated in HCC tissues compared with normal tissues (Fig. 1a and b, Additional file 2: Fig. S2a and b; p < 0.001) [17, 18]. Moreover, DYRK1A was overexpressed in HCC cells compared with normal foetal hepatocytes (Fig. 1c). Additionally, further bioinformatic analysis with the UALCAN database showed that the mRNA level of DYRK1A was increased in HCC tissues compared with normal liver tissues (Fig. 1d). The level of DYRK1A was correlated with the lymph node metastasis status in HCC patients (Fig. 1e) [19]. DYRK1A was also upregulated in primary metastatic HCC tissues compared with nonmetastatic HCC tissues (Fig. 1f; p < 0.05) [20]. Additionally, alteration of DYRK1A expression was correlated with poor outcomes in patients with liver cancer (n = 365: n = 9 in the altered group and n = 356 in the unaltered group; p < 0.05; Fig. 1g) [21, 22]. Moreover, high DYRK1A levels were associated with shorter progression-free survival and progression/metastasis-free survival times in liver cancer patients (Additional file 1: Fig. S1b) [23]. Therefore, we hypothesized that DYRK1A upregulation could be associated with malignant progression and especially metastasis in HCC. Gene–gene Pearson correlation analysis with DYRK1A in HCC clinical samples and functional enrichment analysis revealed that DYRK1A expression was strongly associated with the expression of genes enriched in the cell migration, cell motility and cell–cell junction ontologies, suggesting that DYRK1A could regulate the invasion of HCC cells (Additional file 1: Fig. S1c and Additional file 7: Table S2) [24].

### DYRK1A promotes the migration and invasion abilities of HCC cells

**Fig. 2:**
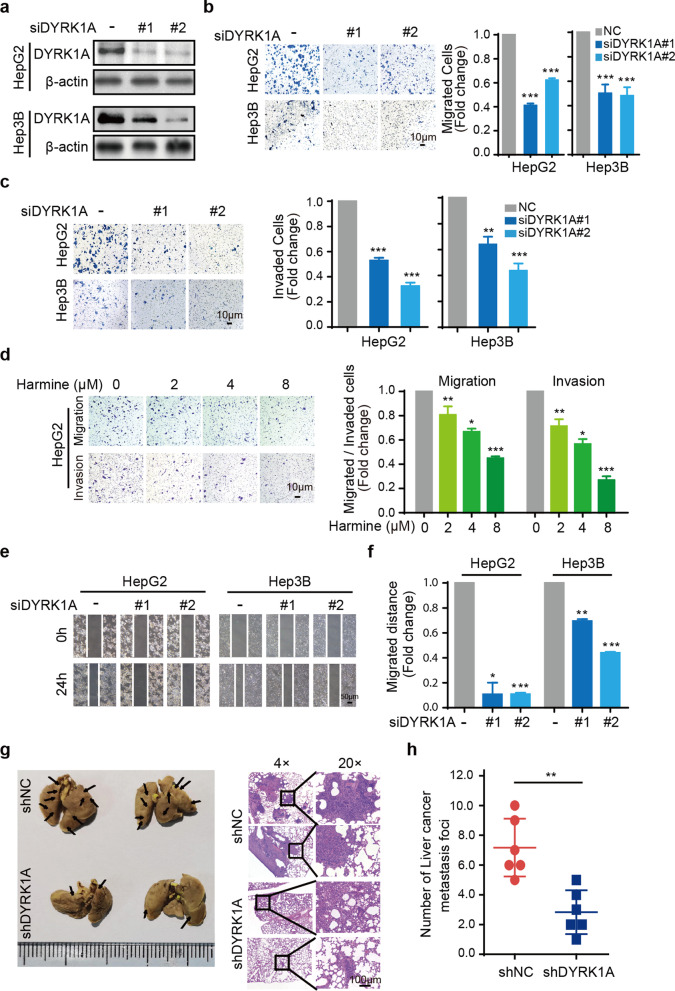
DYRK1A depletion attenuates the metastatic and invasion abilities of HCC cells. **a** HCC cells were incubated with DYRK1A siRNA or control siRNA for 48 h, and western blot analysis was performed. **b** and **c** The migration and invasion abilities of HCC cells incubated with DYRK1A siRNA or control siRNA for 48 h were assessed. **d** HepG2 cells were incubated with harmine and were then cultured in the upper compartment of Transwell chambers for 24 h. Then, the cells were stained with crystal violet solution. **e** and **f** A scratch was made in the HCC cell monolayer using a sterile pipette tip, and the monolayer was then washed with PBS. Images were acquired under a microscope. **g** and **h** DYRK1A-depleted and parental HCC cells were intravenously injected into nude mice (n = 6). Sixty days after injection, lung tissues and other visceral organs were excised from mice in different groups. Tissues were stained with H&E

**Fig. 3:**
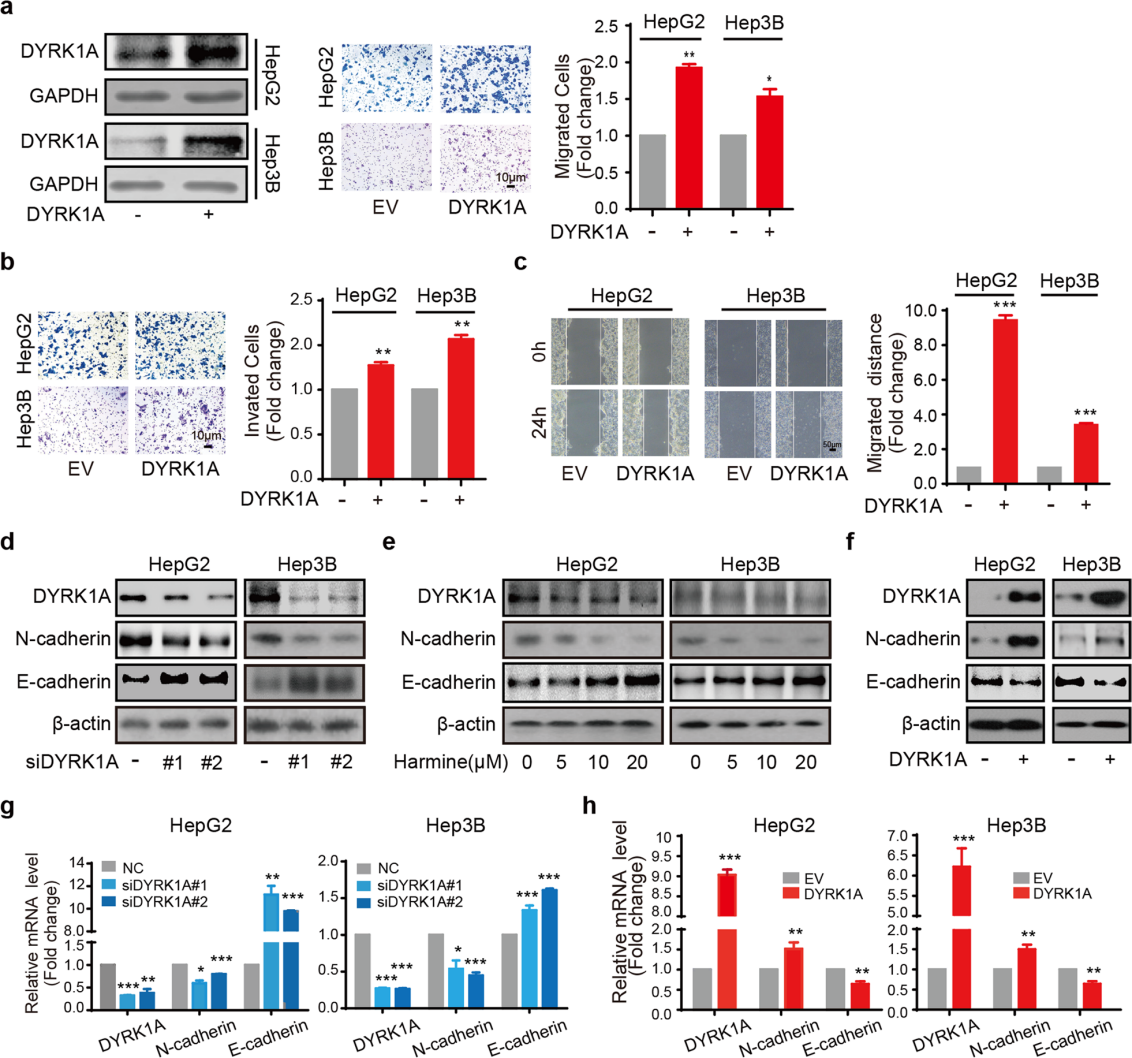
DYRK1A enhances EMT and the migration and invasion abilities of HCC cells. **a** and **b** HCC cells were incubated with the DYRK1A overexpression plasmid or empty vector for 24 h, and cell migration and invasion assays were then carried out. **c** HCC cells were incubated with the DYRK1A overexpression plasmid or empty vector for 24 h, and a scratch was then created. Following incubation for 24 h, images were acquired under a microscope. **d** Western blot analysis was performed in the DYRK1A knockdown group and control group. **e** Following treatment with harmine for 24 h at the indicated concentrations, total protein was extracted from HCC cells and analysed by western blotting. **f** HCC cells were incubated with the DYRK1A overexpression plasmid or empty vector for 48 h. Subsequently, total protein was extracted from HCC cells and analysed by western blotting. **g** HCC cells were incubated with DYRK1A siRNA or control siRNA for 48 h, and mRNA levels were determined. **h** HCC cells were incubated with DYRK1A overexpression plasmid or empty vector for 48 h, and mRNA levels were determined

DYRK1A has been reported to promote cell proliferation and tumour growth in several cancer types [9, 25]. However, the effect of DYRK1A on the proliferation of HCC cells remains unclear. Proliferation was evaluated in HepG2 and Hep3B cells transfected with a DYRK1A small interfering RNA (siRNA) to knock down DYRK1A expression (Fig. 2a). DYRK1A knockdown did not efficiently suppress cell proliferation compared with that in the control siRNA group, and DYRK1A overexpression also could not promote the proliferation of HCC cells (Additional file 3: Fig. S3a and b). However, DYRK1A knockdown inhibited the migration and invasion of HCC cells (Fig. 2b and c). Additionally, treatment of HepG2 cells with harmine, a DYRK1A inhibitor, notably attenuated their migration and invasion in a dose-dependent manner but had little effect on their proliferation (Fig. 2d and Additional file 3: Fig. S3c). Consistent with these findings, the results of wound healing assays indicated the potent antimetastatic effect of DYRK1A depletion in HCC cells (Fig. 2e and f). To further evaluate the role of DYRK1A in metastasis in vivo, we investigated whether DYRKA knockdown can suppress HCC metastasis in vivo. To this end, DYRKA-knockdown HepG2 cells and parental HepG2 cells were intravenously injected into nude mice. As expected, the number of pulmonary metastatic nodules was decreased in the DYRK1A-knockdown cell group compared with the control group (Fig. 2g and h). In contrast, ectopic DYRK1A overexpression clearly enhanced the migration and invasion of HCC cells (Fig. 3a and b). In addition, wound healing assays showed that DYRK1A overexpression significantly increased the migration ability of HCC cells (Fig. 3c). These findings indicated that DYRK1A could promote the metastasis of HCC cells.

### DYRK1A promotes EMT in HCC cells

Epithelial cells go through EMT to acquire mesenchymal characteristics following the dissociation of cell–cell junctions which is crucial in cancer invasion and metastasis, and the hallmark of EMT is the upregulation of N-cadherin followed by the downregulation of E-cadherin [26]. Our data indicated that siRNA- and harmine-induced DYRK1A suppression increased the level of the epithelial marker E-cadherin and reduced the level of the mesenchymal marker N-cadherin, suggesting that DYRK1A silencing can inhibit the EMT process in HCC cells (Fig. 3d and e). In contrast, ectopic DYRK1A overexpression significantly upregulated N-cadherin and downregulated E-cadherin (Fig. 3f). Moreover, in HepG2 and Hep3B cells, DYRK1A silencing significantly increased E-cadherin and reduced N-cadherin mRNA expression, and DYRK1A overexpression increased N-cadherin and decreased E-cadherin mRNA expression (Fig. 3g and h). These findings indicated that DYRK1A could enhance the metastatic potential of HCC cells by promoting EMT.

### The TGF-β/SMAD and JAK/STAT3 pathways are highly enriched in HCC clinical samples with increased DYRK1A expression

**Fig. 4:**
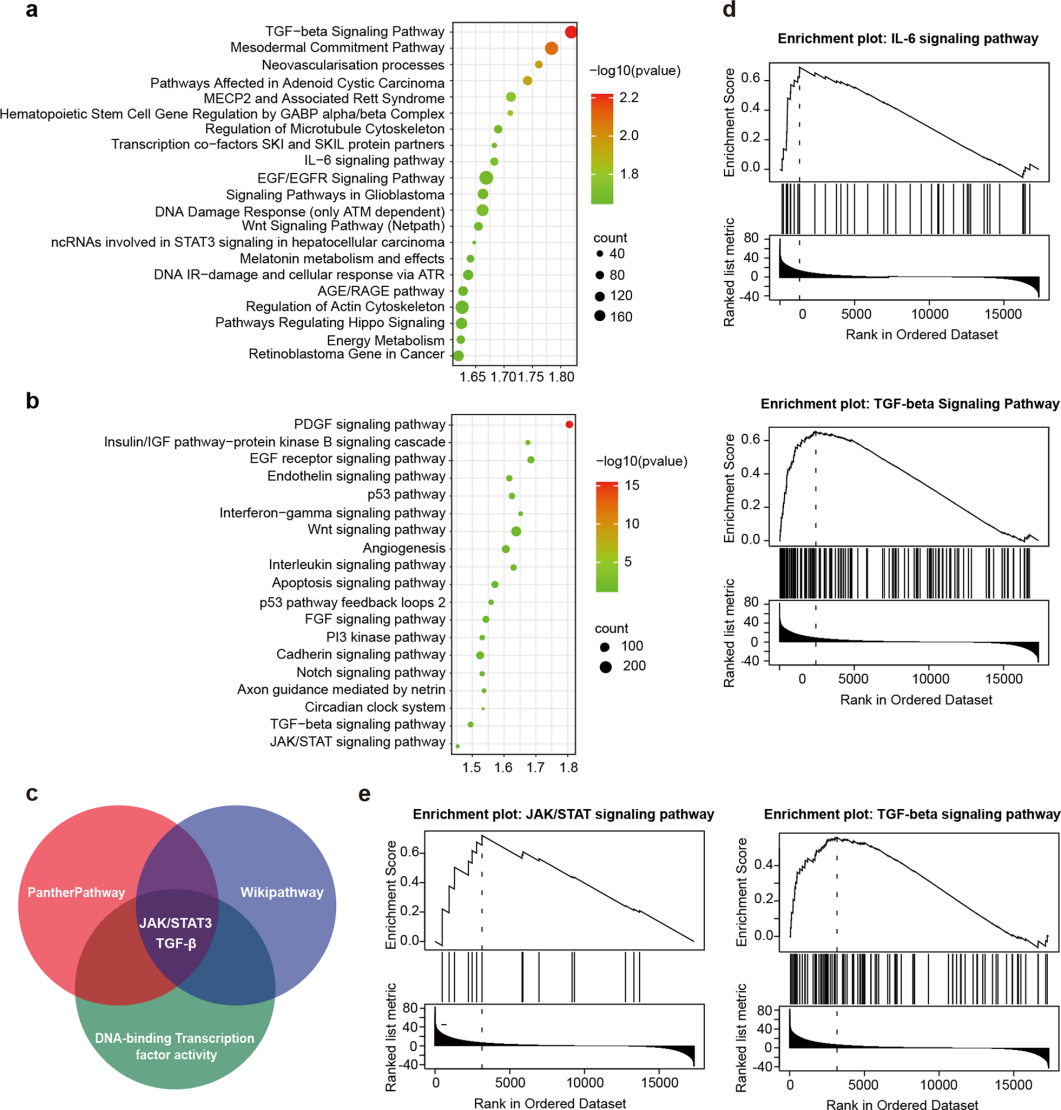
The TGF-β and JAK/STAT3 pathways are highly enriched in HCC clinical samples with increased DYRK1A expression. **a** and **b** Data collected from the LinkedOmics platform (www.linkedomics.org/admin.php) are shown. Sample cohort: The Cancer Genome Atlas_liver HCC; Institute: UNC; Data type: RNAseq; Platform: HiSeq RNA; Attribute: DYRK1A; Statistical methods: Pearson correlation test; Patients: 371; Tool: GSEA, Enrichment Categories: **a** PANTHER pathways and **b** WikiPathways. **c** The TGF-β, JAK/STAT3 and EGF/EGFR signalling pathways were enriched in the DYRK1A high expression group in both the WikiPathways and PANTHER pathways analyses. **d** and **e** GSEA was performed, and the results indicated that DYRK1A could play an important role in regulating the TGF-β and JAK/STAT3 pathways

To better understand the mechanism underlying the effect of DYRK1A on HCC progression, gene set enrichment analysis (GSEA) was performed in HCC samples with low and high DYRK1A expression. GSEA of pathways in the WikiPathways and PANTHER pathway databases was performed using the LinkedOmics platform (Fig. 4a and b; Additional file 8: Table S3 and Additional file 9: Table S4) [24]. As shown in Fig. 4c, bioinformatic analysis with the WikiPathway and PANTHER pathway databases revealed that the TGF-β/SMAD, IL-6/JAK/STAT3 and EGF/EGFR pathways were significantly enriched in the samples with high DYRK1A expression. A previous study by our laboratory demonstrated that DYRK1A could regulate STAT3/EGFR/MET signalling and sensitize EGFR wild-type non-small cell lung cancer (NSCLC) cells to AZD9291, thus indicating that DYRK1A could regulate EGFR via STAT3 [27]. The results of the present study showed that the expression of DYRK1A in clinical samples obtained from patients with HCC could be associated with the activity of DNA-binding transcription factors. Therefore, the SMAD and STAT3 transcription factors could play a critical role in regulating tumorigenesis (Additional file 1: Fig. S1b). These results suggested that DYRK1A could regulate metastasis and EMT via the TGF-β/SMAD and IL-6/JAK/STAT3 signalling pathways (Fig. 4d and e).

### DYRK1A promotes TGF-β/SMAD signalling by interacting with TSC1

**Fig. 5:**
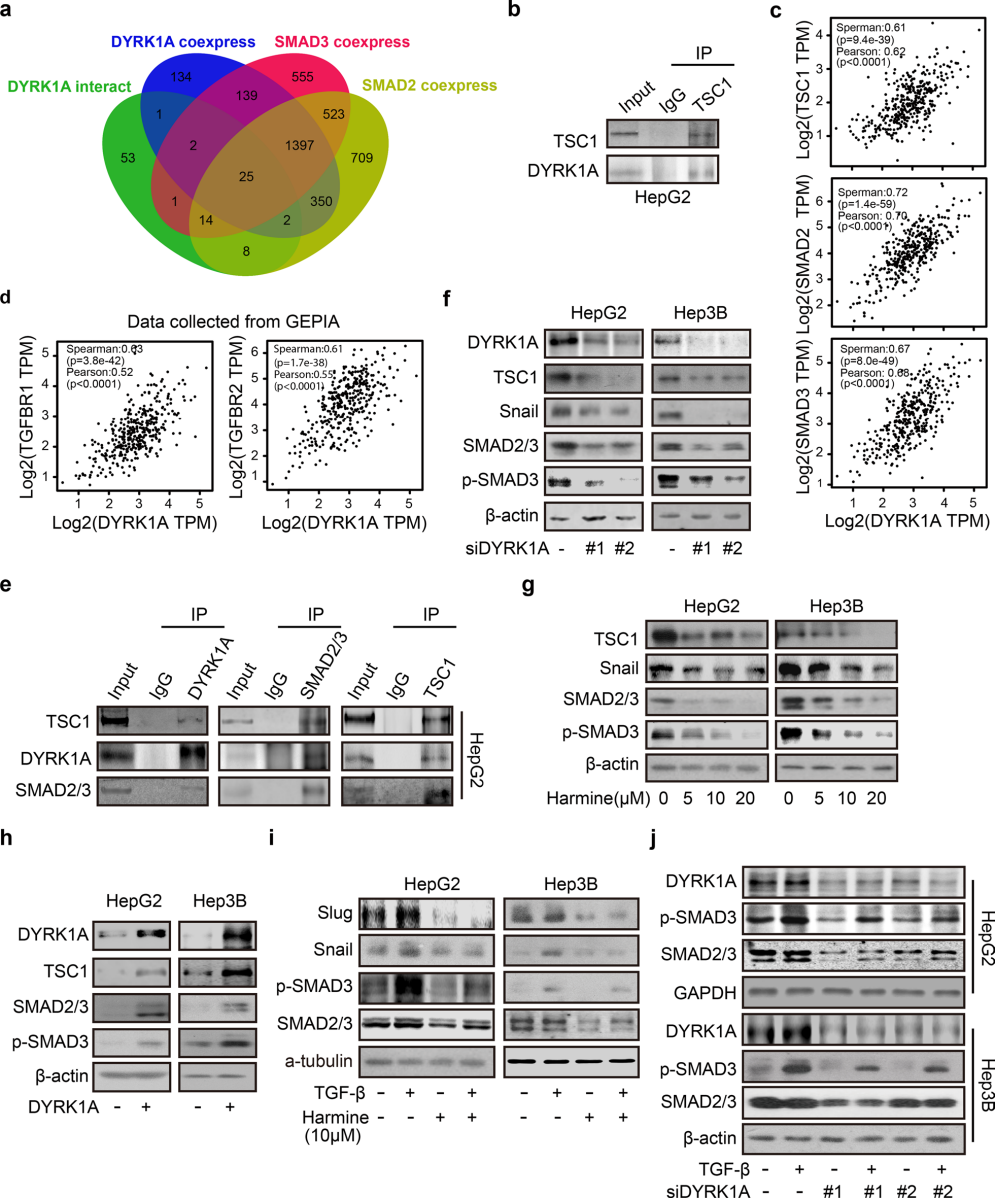
DYRK1A activates TGF-β/SMAD signalling by interacting with TSC1. **a** A diagram was generated using the online tool Venny (http://www.bioinformatics.com.cn/static/others/jvenn/example.html). **b** An immunoprecipitation assay was carried out to assess the interaction between DYRK1A and TSC1. **c** and **d** The results were acquired from the GEPIA database (gepia.cancer-pku.cn). Gene: DYRK1A; The Cancer Genome Atlas Tumour: Liver HCC. **e** An immunoprecipitation assay was performed to evaluate the interaction among DYRK1A, TSC1, and SMAD2/3. **f** DYRK1A was knocked down in HCC cells by transfection with the corresponding siRNA for 48 h. Total protein was extracted from HCC cells and analysed by western blotting. **g** Following treatment with harmine for 24 h, total protein was extracted from HCC cells and analysed. **h** HCC cells were incubated with the DYRK1A overexpression plasmid or empty vector for 48 h, and western blot analysis was performed. **i** HCC cells were serum-starved overnight and were then incubated with 10 μM harmine and/or 10 ng/mL TGF-β for 6 h. Subsequently, protein levels were measured. **j** HCC cells were incubated with DYRK1A siRNA or control siRNA for 24 h. Subsequently, the cells were first serum-starved overnight and then incubated with DMSO or 10 ng/mL TGF-β for 6 h. Western blot analysis was then performed

To gain mechanistic insights into the effects of DYRK1A on activating TGF-β/SMAD signalling and promoting metastasis, the proteins interacting with DYRK1A were analysed using mass spectrometry; in addition, genes coexpressed with DYRK1A, SMAD2 and SMAD3 in liver cancer were identified with the UALCAN platform as those with Pearson correlation coefficient (r) values greater than 0.5 (Additional file 10: Table S5) [19, 28]. To identify the key genes associated with DYRK1A-mediated promotion of TGF-β/SMAD signalling, we generated a Venn diagram, which showed that there were 25 overlapping genes among those coexpressed with DYRK1A, SMAD2 and SMAD3 in liver cancer and DYRK1A-interacting proteins (Fig. 5a) [29]. Furthermore, among the 25 overlapping genes, three genes (DCAF7, EPS15, and TSC1) were overexpressed both in liver cancer tissue compared with normal tissue and in primary HCC tissues of patients with metastasis compared with primary HCC tissues of patients without metastasis, as determined using the online analysis tool HCMDB, indicating that these genes might be involved in the development of HCC (Additional file 4: Fig. S4a). In addition, TSC1 overexpression resulted in poor outcomes in liver cancer patients (p < 0.05), but DCAF7 and EPS15 might not be associated with poor outcomes in liver cancer patients (both p > 0.05; Additional file 4: Fig. S4b) [23, 30]. More importantly, a previous study reported that TSC1 activates TGF-β-Smad2/3 signalling and promotes the EMT process [31]. Thus, we sought to investigate the role of the interaction between DYRK1A and TSC1 in promoting the activation of TGF-β/SMAD signalling and metastasis.

Immunoprecipitation assays verified that DYRK1A can interact with TSC1 in HCC cells (Fig. 5b). Furthermore, DYRK1A and TSC1 were coexpressed in clinical tumour samples from patients with HCC (Spearman correlation analysis; r = 0.61, p < 9.4e–39; Pearson correlation analysis, r = 0.62, p < 0.0001; Fig. 5c) [32]. Therefore, it was hypothesized that DYRK1A could activate TGF-β/SMAD signalling by interacting with TSC1. Thus, the associations of the expression of TGF-β/SMAD signalling-related genes and the expression of DYRK1A with tumour metastasis were first investigated. As shown in Fig. 5d, DYRK1A was coexpressed with TGF-β/SMAD signalling-related genes, such as TGF-β receptor 1, TGF-β receptor 2, SMAD2 and SMAD3, in clinical samples from patients with HCC [32]. Additionally, immunoprecipitation assays revealed that DYRK1A can interact with TSC1 and SMAD2/3, indicating that the DYRK1A-TSC1-SMAD2/3 complex might play a vital role in HCC tumorigenesis (Fig. 5e). Furthermore, DYRK1A knockdown in HCC cells by siRNA or inhibition with harmine suppressed the expression of TSC1/TGF-β/SMAD signalling-related genes, such as TSC1, snail, SMAD2 and SMAD3 (Fig. 5f and g). Meanwhile, TSC1 knockdown downregulated DYRK1A and SMAD2/3 (Additional file 5: Fig. S5). In contrast, DYRK1A overexpression increased the expression levels of TSC1/TGF-β/SMAD signalling-related genes (Fig. 5h). Finally, DYRK1A silencing or cell treatment with harmine attenuated the ability of TGF-β to activate the SMAD signalling pathway (Fig. 5i and j). Overall, these results suggested that DYRK1A could activate the TGF-β/SMAD pathway by interacting with TSC1 and SMAD2/3.

### DYRK1A promotes EMT and enhances the metastatic potential of HCC cells through the TSC1/TGF-β/SMAD pathway

**Fig. 6:**
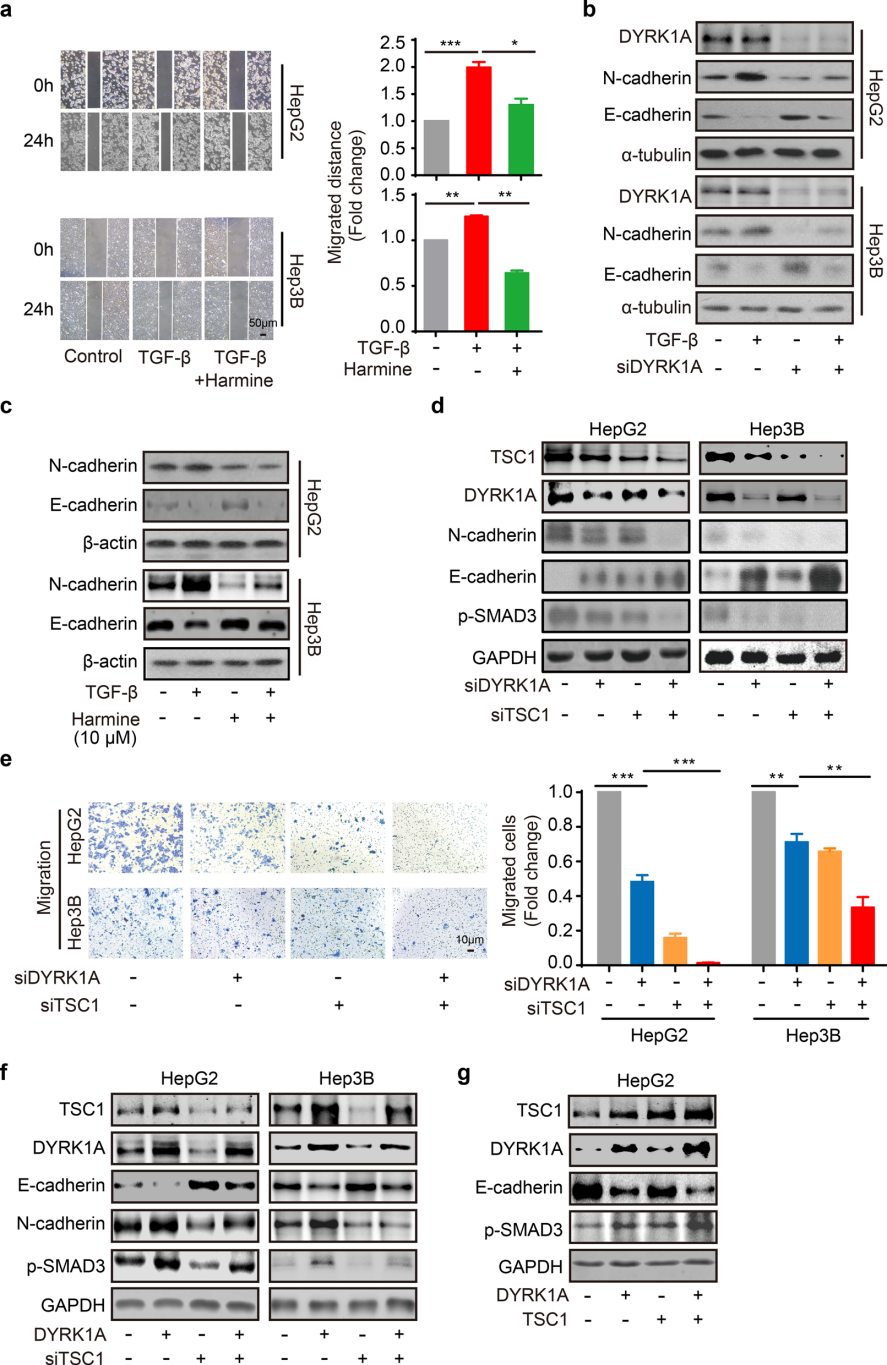
DYRK1A enhances EMT and the metastatic potential of HCC cells via TSC1/TGF-β/SMAD signalling. **a** HCC cells were serum-starved overnight, a scratch was created, and the cells were then incubated with 10 μM harmine and/or 10 ng/mL TGF-β for 6 h. Images were acquired under a microscope. **b** HCC cells were transfected with DYRK1A siRNA or control small interfering RNA for 24 h. HCC cells were serum-starved overnight and were then treated with DMSO or 10 ng/mL TGF-β for 6 h. Total protein was extracted from HCC cells, and western blot analysis was then performed. **c** Cells were serum-starved overnight and were then treated with harmine or 10 ng/mL TGF-β for 6 h. Western blot analysis was then performed. **d** and **e** The levels of the indicated proteins and the cell migration abilities were assessed in DYRK1A- or TSC1-depleted HCC cells. **f** The levels of the indicated proteins were assessed in DYRK1A overexpressed and/or TSC1-depleted HCC cells. **g** The levels of the indicated proteins were assessed in DYRK1A and/or TSC1 overexpressed HCC cells

HCC cell treatment with harmine significantly attenuated the TGF-β-triggered migration of HepG2 and Hep3B cells (Fig. 6a). Subsequently, our results demonstrated that DYRK1A silencing or cell treatment with harmine inhibited TGF-β-induced EMT (Fig. 6b and c). In addition, TSC1 silencing restored the suppressive effect of DYRK1A knockdown on the EMT process in HCC cells (Fig. 6d). Furthermore, TSC1 knockdown enhanced the inhibitory effect of DYRK1A depletion on the migration ability of HCC cells (Fig. 6e). Moreover, TSC1 silencing reversed the enhancement of the EMT process and SMAD3 activation induced by DYRK1A overexpression in HCC cells (Fig. 6f). In addition, TSC1 and DYRK1A overexpression could enhance the activation of EMT process and SMAD3 compared with either TSC1 or DYRK1A overexpression (Fig. 6g). Therefore, these results indicated that DYRK1A could enhance EMT and the metastatic potential of HCC cells via the TSC1/TGF-β/SMAD signalling pathway.

### DYRK1A promotes EMT and the metastatic potential of HCC cells by activating STAT3

**Fig. 7:**
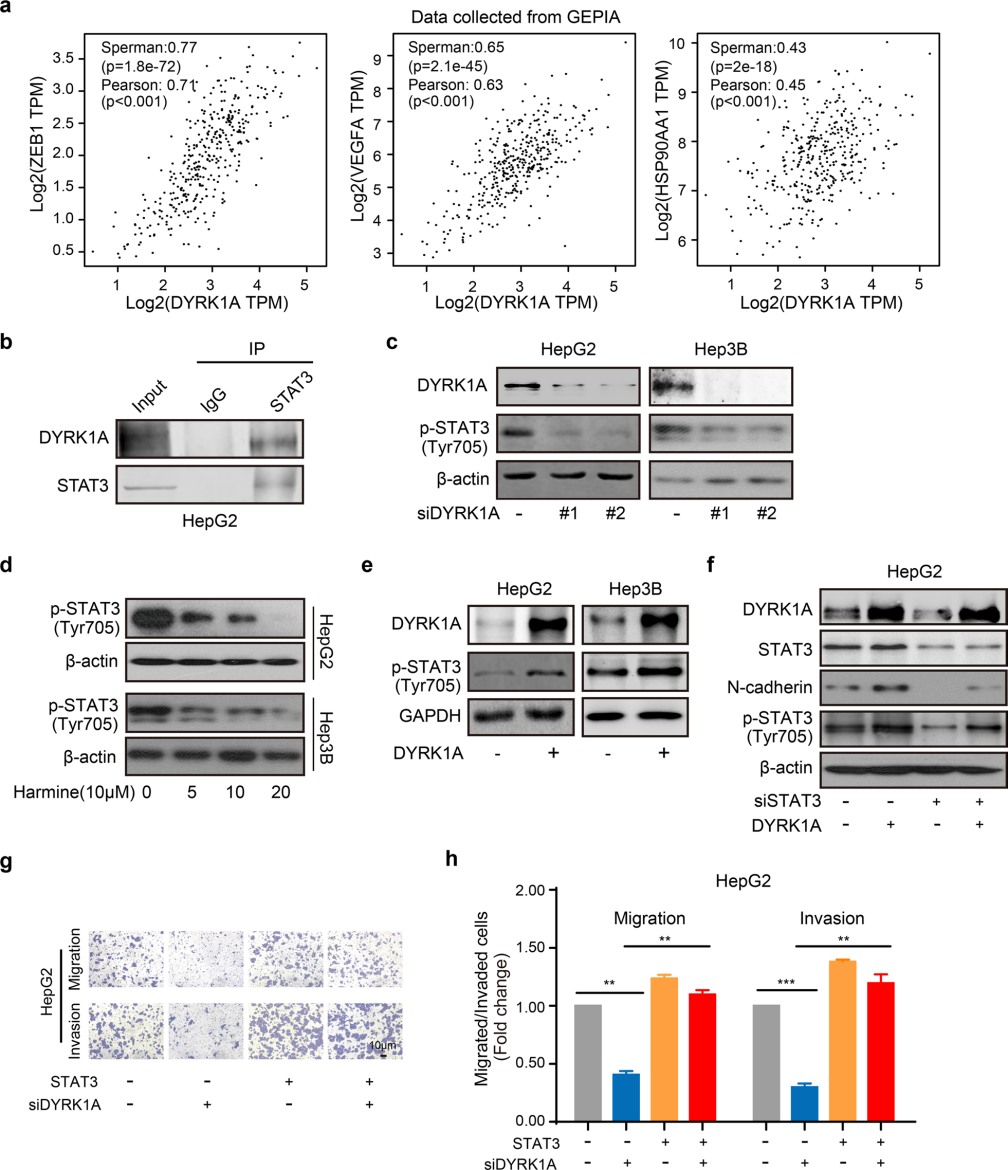
DYRK1A enhances EMT and the metastatic potential of HCC cells by activating STAT3. **a** The coexpression data were acquired from GEPIA (gepia.cancer-pku.cn). Gene: DYRK1A; The Cancer Genome Atlas Tumour: liver HCC. **b** The interaction between DYRK1A and STAT3 was verified by immunoprecipitation. **c** The levels of p-STAT3 were determined by western blot analysis in HCC cells transfected with DYRK1A siRNA. **d** HCC cells were incubated with harmine for 24 h, and western blot analysis was performed. **e** The levels of p-STAT3 in HCC cells with or without DYRK1A overexpression are shown. **f** The levels of the indicated proteins were determined in cells transfected with the DYRK1A overexpression plasmid or STAT3 siRNA for 48 h. **g** and **h** HCC cells were incubated with DYRK1A siRNA or the STAT3 overexpression plasmid for 48 h, and the cell migration and invasion abilities were assessed

A previous study by our laboratory showed that DYRK1A could affect the antitumor activity of AZD9291 by regulating STAT3 signalling in NSCLC [27]. Therefore, it was hypothesized that DYRK1A could enhance HCC cell metastasis by regulating STAT3. DYRK1A was found to be coexpressed with several STAT3 target genes, including zinc finger E-box-binding homeobox 1, VEGFA and heat shock protein 90, in clinical tumour samples from patients with HCC (Fig. 7a). Moreover, the interaction between DYRK1A and STAT3 in HCC cells was verified by immunoprecipitation, as shown in Fig. 7b. Furthermore, DYRK1A deletion by siRNA or harmine significantly inhibited the activation of STAT3 in HCC cells (Fig. 7c and d). In contrast, DYRK1A overexpression notably upregulated STAT3 (Tyr705) phosphorylation (Fig. 7e), while STAT3 depletion attenuated DYRK1A-induced EMT in HCC cells (Fig. 7f). Furthermore, STAT3 overexpression abrogated the inhibitory effect of DYRK1A depletion on the migration of HCC cells (Fig. 7g and h). These findings indicated that DYRK1A could promote EMT and the metastatic potential of HCC cells by activating STAT3.

### DYRK1A promotes EMT by cooperatively activating the STAT3/SMAD axis

**Fig. 8:**
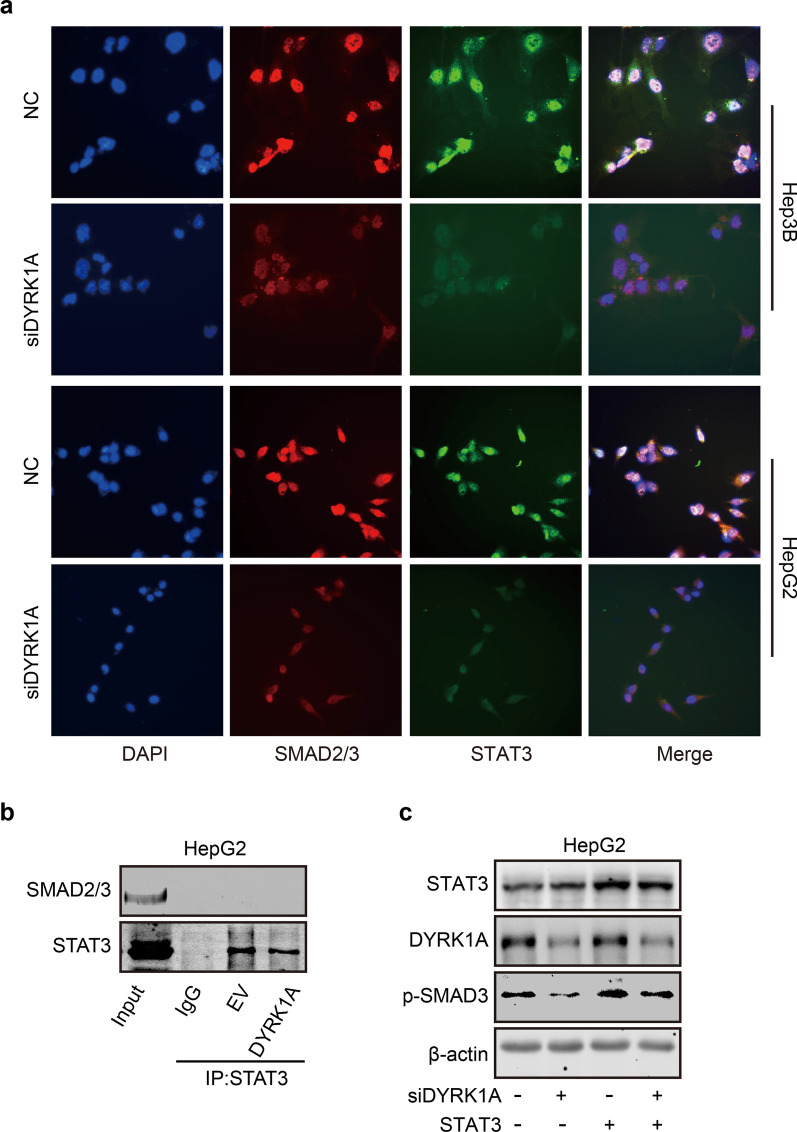
DYRK1A promotes EMT by activating a cooperative STAT3/SMAD gene transcription program. **a** HCC cells were incubated with DYRK1A siRNA or negative control siRNA for 48 h, and an immunofluorescence assay was then performed. **b** The interaction of STAT3 and SMAD2/3 in empty vector and DYRK1A overexpression group. **c** HCC cells were transfected with DYRK1A siRNA or the STAT3 overexpression plasmid for 48 h, and western blot analysis was then performed

Here, we hypothesized that DYRK1A-induced EMT was mediated not only by STAT3 but also potentially by TSC1/TGF-β/SMAD signalling. To assess whether DYRK1A can promote the interaction between STAT3 and SMAD2/3, an immunofluorescence assay was performed. DYRK1A depletion not only decreased the nuclear translocation of STAT3 and SMAD2/3 but also inhibited the colocalization of STAT3 and SMAD3 in the nucleus in HCC cells (Fig. 8a). However, as shown in Fig. 8b, the direct interaction of STAT3 and SMAD2/3 was not observed both on empty vector and DYRK1A overexpression groups in HCC cells. These data indicated that DYRK1A promoted the activation of STAT3 and SMAD2/3 independently, and the interaction of STAT3 and SMAD2/3 could not be observed in DYRK1A overexpression group. However, STAT3 overexpression restored DYRK1A depletion-induced SMAD3 downregulation in HCC cells (Fig. 8c). These results indicated that DYRK1A could promote EMT by activating a cooperative STAT3/SMAD gene transcription program.

## Discussion

DYRK1A has been reported to play a multifaceted role in tumorigenesis. Several studies have indicated that DYRK1A acts as a tumour suppressor gene, while others have highlighted its pro-oncogenic activity [8]. For instance, DYRK1A could activate NFATC1 to increase the migration ability of glioblastoma cells, while it could play an important role in maintaining cancer stemness in oral squamous cell carcinoma [15, 33]. In contrast, another study showed that DYRK1A could act as a tumour suppressor by downregulating c-Myc in acute myeloid leukaemia cells [34]. Therefore, it was hypothesized that the effect of DYRK1A on tumorigenesis was highly dependent on the cellular context [35]. Previous studies from our laboratory demonstrated that DYRK1A could act as an oncogene in lung tumorigenesis [27, 36, 37]. Therefore, exploring the role of DYRK1A in HCC tumorigenesis is of great importance. Selective inhibitors of DYRK1A have been extensively explored and are considered potential candidates for treating multiple diseases, including Down’s syndrome, Alzheimer’s disease, Parkinson’s disease and cancer [38]. Therefore, uncovering the effect of DYRK1A on HCC tumorigenesis could help identify cancer patients who are most likely to benefit from treatment with DYRK1A inhibitors. The current study first demonstrated that DYRK1A was upregulated in HCC tissues compared with normal liver tissues. Furthermore, DYRK1A overexpression enhanced the metastatic ability of HCC cells both in vitro and in vivo. These findings indicated that DYRK1A could function as an oncogene in HCC tumorigenesis. A recent study by Li et al. [39] showed that DYRK1A could directly bind to trophinin-associated protein to promote cell cycle progression in HCC cells by activating the Akt/GSK-3β signalling pathway. The results of the current study highlighting the role of DYRK1A in promoting HCC tumorigenesis could also be supported by the above study.

The JAK/STAT3 and TGF-β/SMAD signalling pathways play critical roles in regulating cancer cell metastasis and can synergistically enhance EMT and the metastatic ability of cancer cells [4]. Therefore, it is important to develop molecular drugs that can regulate both the JAK/STAT3 and TGF-β/SMAD signalling pathways to treat patients with metastatic cancer. Herein, DYRK1A was found to be coexpressed with members of the TGF-β/SMAD and STAT3 signalling pathways in clinical tumour samples from patients with HCC. Furthermore, DYRK1A activated TGF-β/SMAD signalling by interacting with TSC1 and enhanced the metastatic ability of HCC cells by interacting and activating STAT3. Recently, Bhansali et al. [40] demonstrated that DYRK1A could regulate the development of B-cell acute lymphoblastic leukaemia by binding to and phosphorylating STAT3. This work was consistent with and supported our findings. In addition, the results of the current study suggested that DYRK1A could induce EMT by promoting a cooperative STAT3/SMAD gene transcription program. Although STAT3 and SMAD are attractive therapeutic targets for cancer, they have been shown to be notoriously difficult to target with small molecule inhibitors in clinical trials [41, 42]. In addition, the results of the present work suggested that DYRK1A depletion could simultaneously inhibit the activation of JAK/STAT3 and TGF-β/SMAD signalling. Therefore, DYRK1A could be considered a novel therapeutic target for metastatic HCC. When STAT3 is aberrantly activated in tumours, STAT3 selectively and directly interacts with Smad3, sequesters Smad3 from the Smad nucleoprotein complex and thus antagonizes TGF-β signalling [43]. Thus, the physically interaction of STAT3 and SMAD3 can attenuate the process of EMT, our data indicated that the interaction of STAT3 and SMAD3 could not be observed in HCC cells, and DYRK1A overexpression did not affect the interaction of STAT3 and SMAD3 in HCC cells. These data suggested that DYRK1A cooperatively activated STAT3 and SMAD signalling in an independent way. However, the crosstalk between STAT3 and SMAD signalling activated by DYRK1A could be observed in HCC cells, which is consistence with literature [6]. For example, STAT3 overexpression upregulated SMAD2/3 in HCC cells, while STAT3 silencing exhibited the opposite effect (Additional file 5: Fig. S5b). However, the mechanism of crosstalk between STAT3 and Smad signalling activated by DYRK1A may need further investigation.

It has been reported that the interaction of TSC1 with the TGF-β receptor complex and SMAD2/3 is required for TGF-β1-mediated SMAD2/3 phosphorylation and EMT [31]. Furthermore, PI3K activation and phosphorylation of Akt at Thr308 can result in phosphorylation and inhibition of the TSC1 complex. Therefore, Akt was identified as the first kinase to directly phosphorylate and negatively regulate the TSC1 complex [44, 45]. Mature DYRK1A, a dual-specificity protein kinase, phosphorylates substrates on serine or threonine (S/T) residues [46]. Several kinases have been reported to phosphorylate TSC1 on S/T residues. For example, polo-like kinase 1 can phosphorylate TSC1 at S467 and S578 to destabilize TSC1 [47]. The current study revealed that DYRK1A can directly bind to TSC1 and positively regulate its expression, thus suggesting that TSC1 could be a novel substrate of DYRK1A in HCC cells. Additionally, DYRK1A can bind to and regulate SMAD2/3 by directly interacting with TSC1 in HCC cells. However, the binding sites of between DYRK1A and TSC1 should be further determined. It has been reported that DYRK1A increases the stability of its substrates, such as NFATC1, by phosphorylating and interfering with their ubiquitin-mediated proteasomal degradation [15]. Our data indicated that DYRK1A enhanced the expression of TSC1 and that TSC1 enhanced the expression of DYRK1A. Thus, we hypothesized that DYRK1A phosphorylated and inhibited the ubiquitin-mediated proteasomal degradation of TSC1, subsequently increasing the stability of TSC1. However, further investigation might be needed to verify this hypothesis.

## Conclusions

**Fig. 9:**
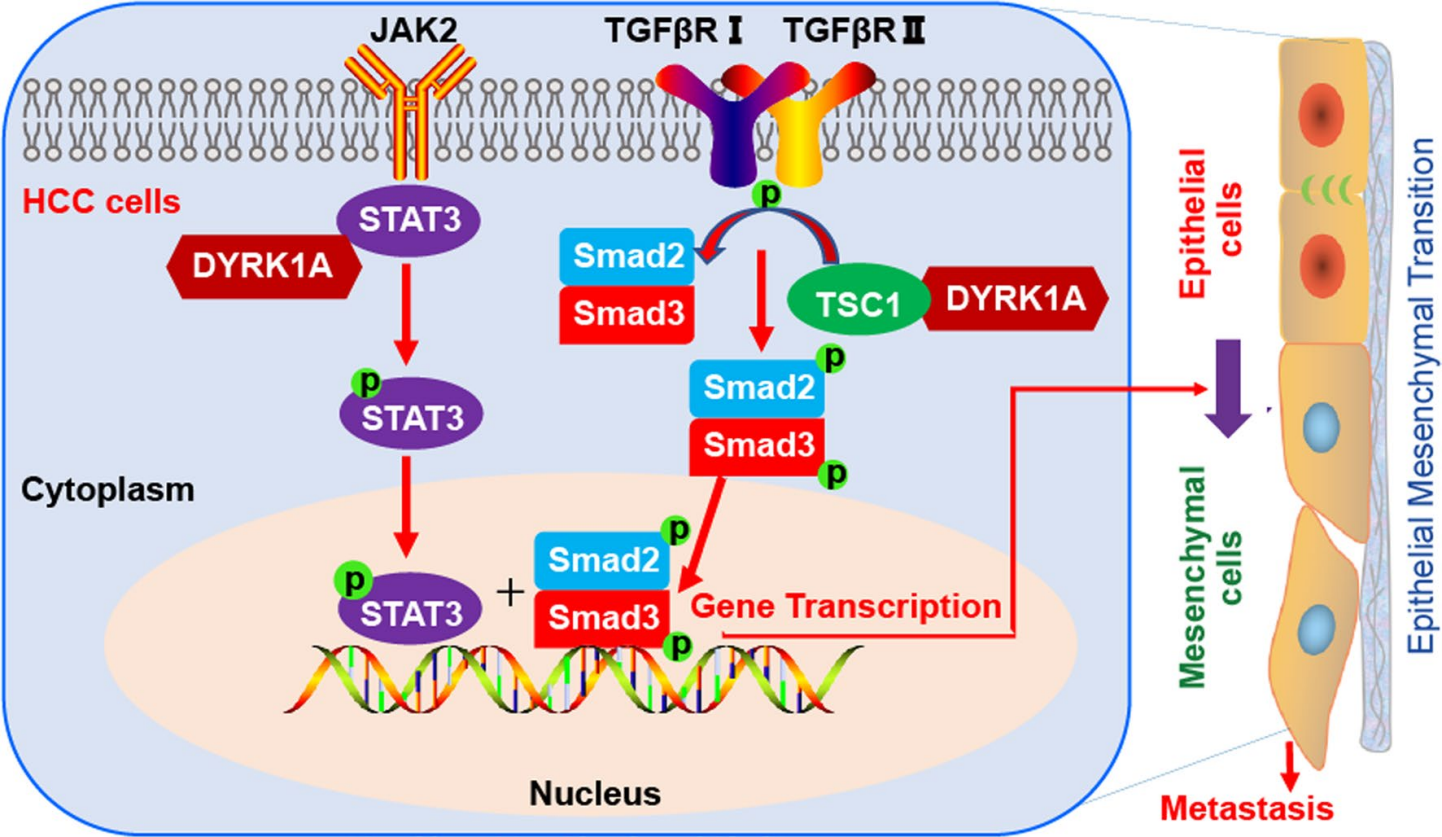
DYRK1A promotes EMT and metastasis of hepatocellular carcinoma cells by cooperatively activating STAT3 and SMAD. DYRK1A interacted with TSC1 and activated SMAD2/3 and also interacted with and activated STAT3 in HCC cells. Thus, DYRK1A promoted EMT and metastasis of HCC cells by activating STAT3 and SMAD gene transcription program in an independent way

Collectively, this work suggested that DYRK1A could be a potential biomarker for the diagnosis and prognosis of patients with HCC. In addition, DYRK1A could promote HCC tumorigenesis by enhancing the metastatic ability of HCC cells. Furthermore, DYRK1A promoted HCC cell EMT and metastasis by activating a cooperative STAT3/SMAD gene transcription program (Fig. 9). Therefore, this study not only uncovered the effect of DYRK1A on promoting HCC metastasis and revealed the underlying mechanism but also provided a novel therapeutic approach for improving the prognosis and treatment of patients with metastatic HCC.

## Supplementary Information

**Additional file 1: Figure S1.** DYRK1A might be involved in the metastasis of HCC cells. (a) Highly invasive HepG2 cells were established by serial selection via a Transwell assay. (b) The effect of DYRK1A on the progression-free survival and progression/metastasis-free survival of liver cancer patients. The data were collected from Kaplan–Meier Plotter (http://kmplot.com/analysis/index.php?p=background). Gene: DYRK1A; Survival: PFS, n = 370 (left panel); Survival: PFS/RFS, Vascular invasion: none, n = 205 (right panel) (c) Data collected from the LinkedOmics platform (www.linkedomics.org/admin.php) are shown. Sample cohort: TCGA_LIHC; Institute: UNC; Data type: RNAseq; Platform: HiSeq RNA; Attribute: DYRK1A; Statistical methods: Pearson correlation test; Patients: 371; Tool: overrepresentation enrichment analysis; Functional database: Gene ontology analysis (biological process, cellular component and molecular function).

**Additional file 2: Figure S2.** Expression of DYRK1A in HCC samples. Compared with that in (a) normal liver tissues, DYRK1A expression was upregulated in (b) HCC tissues.

**Additional file 3: Figure S3.** DYRK1A suppression failed to suppress cell proliferation. (a) HCC cells were incubated with the DYRK1A overexpression plasmid or empty vector for 48 h, and the expression of DYRK1A was detected (upper panel). HCC cells were transfected with DYRK1A plasmid or empty vector for 24 h at 6-well plates, then transferred to 96-well plated for the indicated times, and finally SRB assay was performed (lower panel). (b) HCC cells were incubated with DYRK1A siRNA or control siRNA for 24 h at 6-well plates, then transferred to 96-well plated for the indicated times, and finally SRB assay was performed. (c) HCC cells were incubated with harmine, and an SRB assay was performed.

**Additional file 4: Figure S4.** TSC1 was involved in DYRK1A-mediated promotion of metastasis. (a) The online analysis tool HCMDB was used to investigate the expression of 25 overlapping genes in the indicated tissues (http://hcmdb.i-sanger.com/index). (b) Prognostic value of TSC1, DCAF7 and EPS15 in patients with liver cancer (http://kmplot.com/analysis/index.php?p=background).

**Additional file 5: Figure S5.** TSC1 knockdown downregulated SMAD2/3 in HCC cells. (a) HCC cells were transfected with TSC1 siRNA or control small interfering RNA for 48 h, and the expression levels of the indicated proteins were assessed by western blotting. (b) HCC cells were incubated with the STAT3 overexpression plasmid or STAT3 siRNA for 48 h, and western blot analysis was then performed.

**Additional file 6: Table S1.** RNA sequencing analysis of gene expression in highly invasive and weakly invasive HepG2 cells. RNA sequencing was performed to compare gene transcription levels between P5 and P0.

**Additional file 7: Table S2.** Overrepresentation enrichment analysis of DYRK1A in clinical samples obtained from patients with hepatocellular carcinoma.

**Additional file 8: Table S3.** GSEA of hepatocellular carcinoma samples with low and high DYRK1A expression using the LinkedOmics platform by WikiPathways enrichment.

**Additional file 9: Table S4.** GSEA of hepatocellular carcinoma samples with low and high DYRK1A expression using the LinkedOmics platform by PANTHER pathways enrichment.

**Additional file 10: Table S5.** The gene lists of DYRK1A interacting and co-expressed proteins and SMAD2/3 positively co-expressed proteins.

## Data Availability

The data presented in the study are included in the article and additional material.

## Abbreviations

HCC: Hepatocellular carcinoma
DYRK1A: Dual-specificity tyrosine phosphorylation-regulated kinase 1A
c-MET: C-mesenchymal-epithelial transition receptor
EMT: Epithelial-mesenchymal transition
TGF-β: Transforming growth factor-β
TSC1: Tuberous sclerosis 1
FBS: Fetal bovine serum
H&E: Hematoxylin and eosin
siRNA: Small interfering RNA
GSEA: Gene set enrichment analysis
S/T: Serine or threonine
DMEM: Dulbecco’s modified Eagle medium
PVDF: Polyvinylidene fluoride
STAT3: Signal transducer and activator of transcription 3
JAK: Janus kinase
