# Supplementary figures and images for: DYRK1A reinforces epithelial-mesenchymal transition and metastasis of hepatocellular carcinoma via cooperatively activating STAT3 and SMAD
Source: J Biomed Sci. 2022 Jun 2;29:34. doi: 10.1186/s12929-022-00817-y (PMC9164892; doi:10.1186/s12929-022-00817-y)

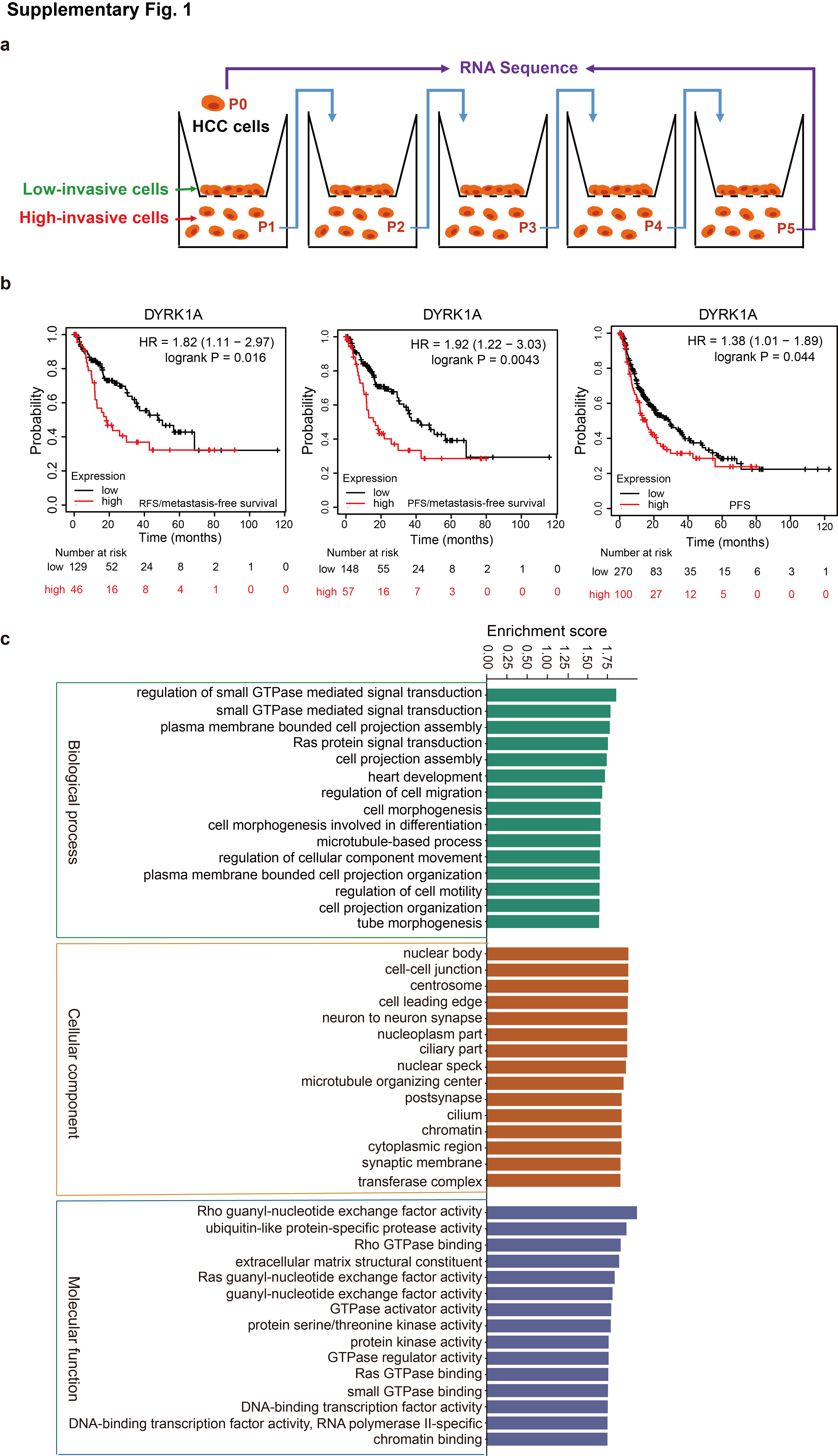

Supplement: Supplementary file 1 — Additional file 1: Figure S1. DYRK1A might be involved in the metastasis of HCC cells. (a) Highly invasive HepG2 cells were established by serial selection via a Transwell assay. (b) The effect of DYRK1A on the progression-free survival and progression/metastasis-free survival of liver cancer patients. The data were collected from Kaplan–Meier Plotter (http://kmplot.com/analysis/index.php?p=background). Gene: DYRK1A; Survival: PFS, n = 370 (left panel); Survival: PFS/RFS, Vascular invasion: none, n = 205 (right panel) (c) Data collected from the LinkedOmics platform (www.linkedomics.org/admin.php) are shown. Sample cohort: TCGA_LIHC; Institute: UNC; Data type: RNAseq; Platform: HiSeq RNA; Attribute: DYRK1A; Statistical methods: Pearson correlation test; Patients: 371; Tool: overrepresentation enrichment analysis; Functional database: Gene ontology analysis (biological process, cellular component and molecular function). [file 12929_2022_817_MOESM1_ESM.tif]

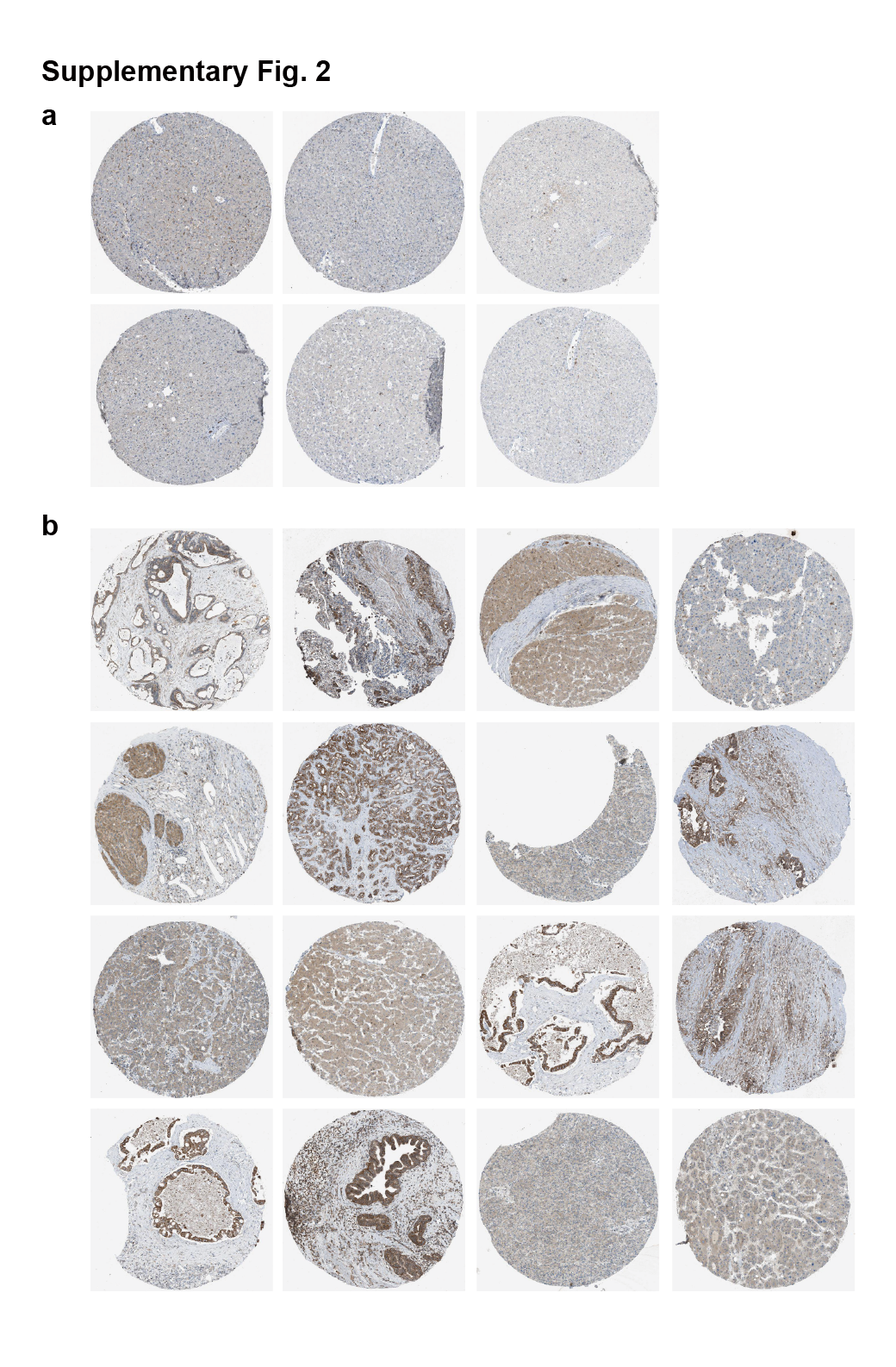

Supplement: Supplementary file 2 — Additional file 2: Figure S2. Expression of DYRK1A in HCC samples. Compared with that in (a) normal liver tissues, DYRK1A expression was upregulated in (b) HCC tissues. [file 12929_2022_817_MOESM2_ESM.tif]

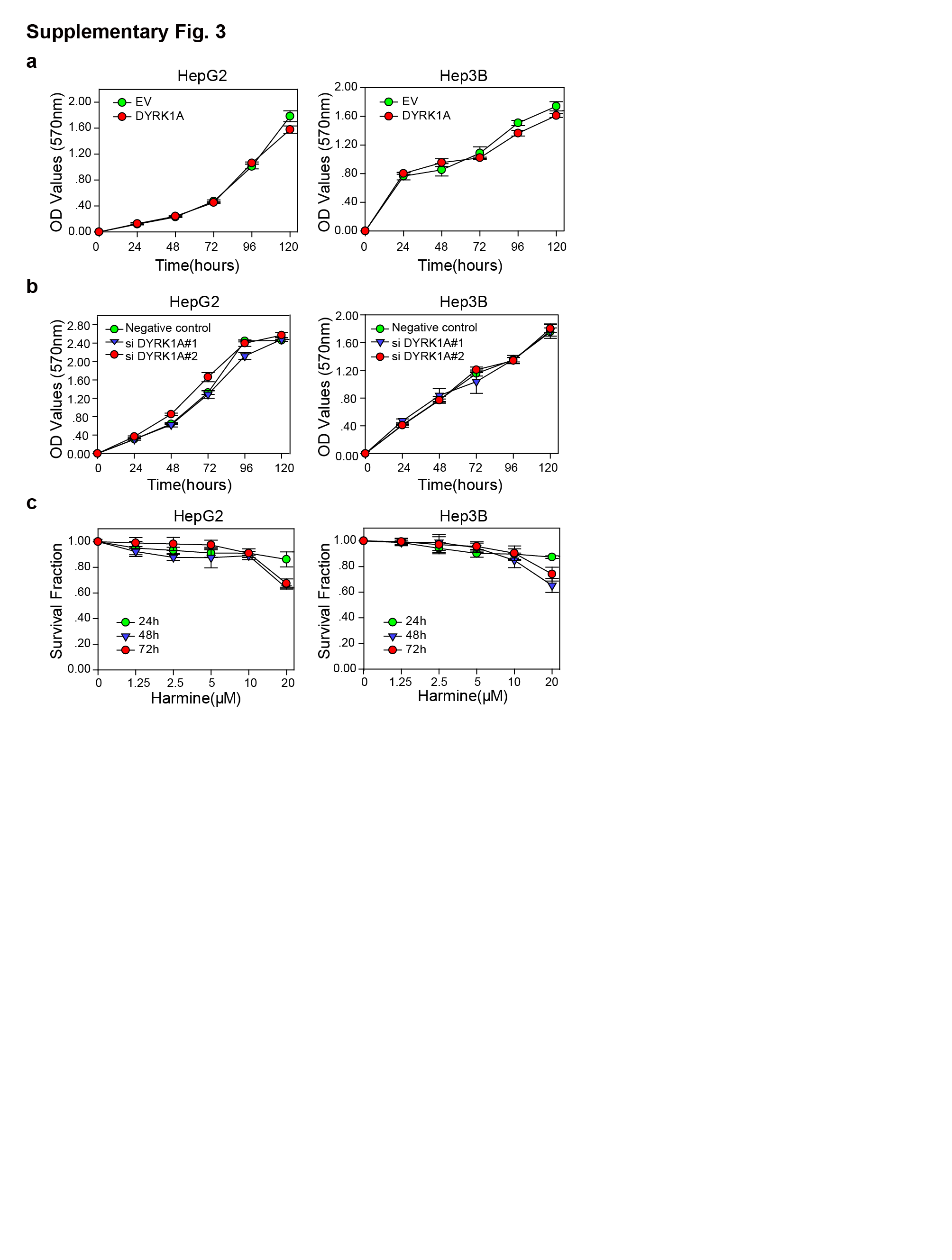

Supplement: Supplementary file 3 — Additional file 3: Figure S3. DYRK1A suppression failed to suppress cell proliferation. (a) HCC cells were incubated with the DYRK1A overexpression plasmid or empty vector for 48 h, and the expression of DYRK1A was detected (upper panel). HCC cells were transfected with DYRK1A plasmid or empty vector for 24 h at 6-well plates, then transferred to 96-well plated for the indicated times, and finally SRB assay was performed (lower panel). (b) HCC cells were incubated with DYRK1A siRNA or control siRNA for 24 h at 6-well plates, then transferred to 96-well plated for the indicated times, and finally SRB assay was performed. (c) HCC cells were incubated with harmine, and an SRB assay was performed. [file 12929_2022_817_MOESM3_ESM.tif]

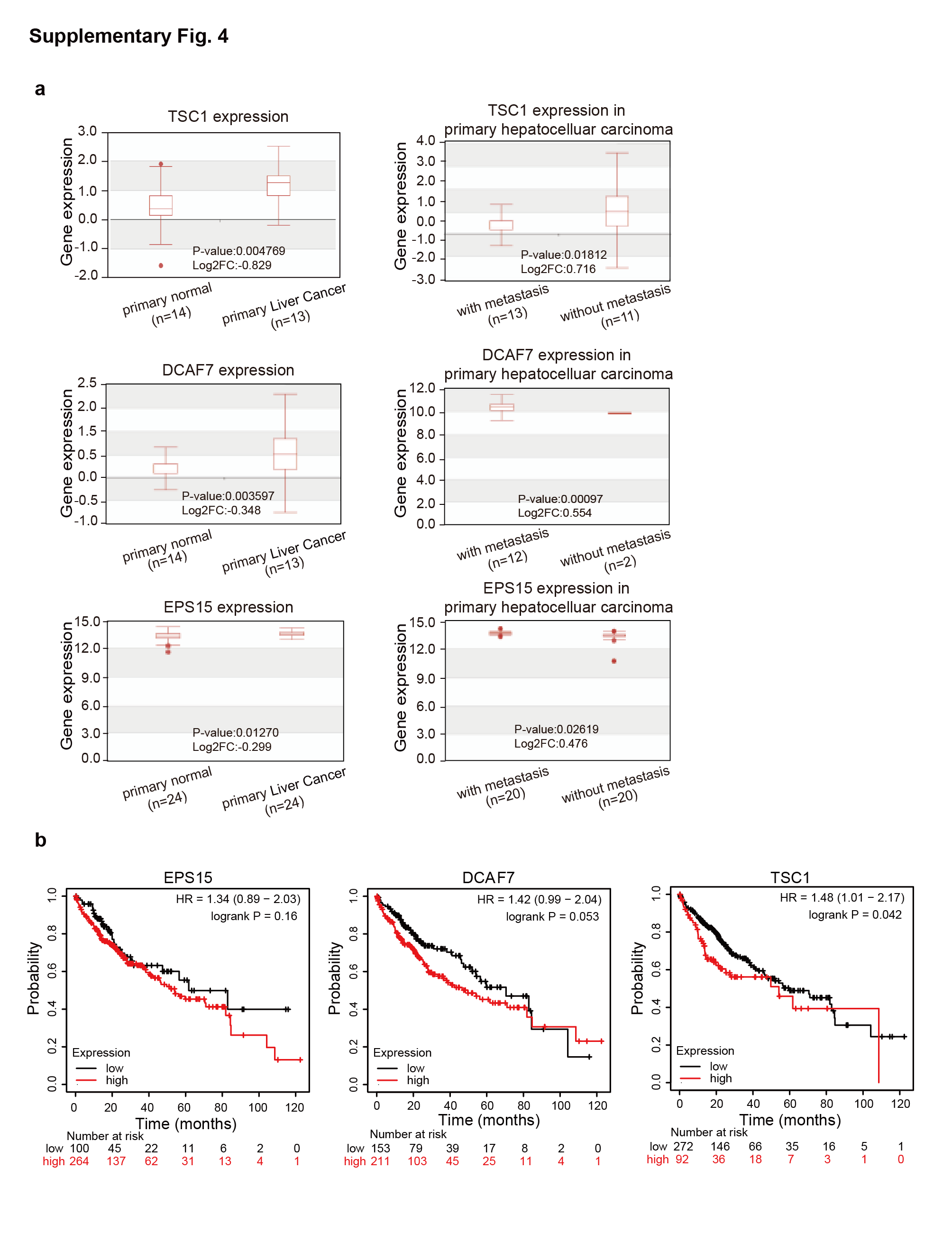

Supplement: Supplementary file 4 — Additional file 4: Figure S4. TSC1 was involved in DYRK1A-mediated promotion of metastasis. (a) The online analysis tool HCMDB was used to investigate the expression of 25 overlapping genes in the indicated tissues (http://hcmdb.i-sanger.com/index). (b) Prognostic value of TSC1, DCAF7 and EPS15 in patients with liver cancer (http://kmplot.com/analysis/index.php?p=background). [file 12929_2022_817_MOESM4_ESM.tif]

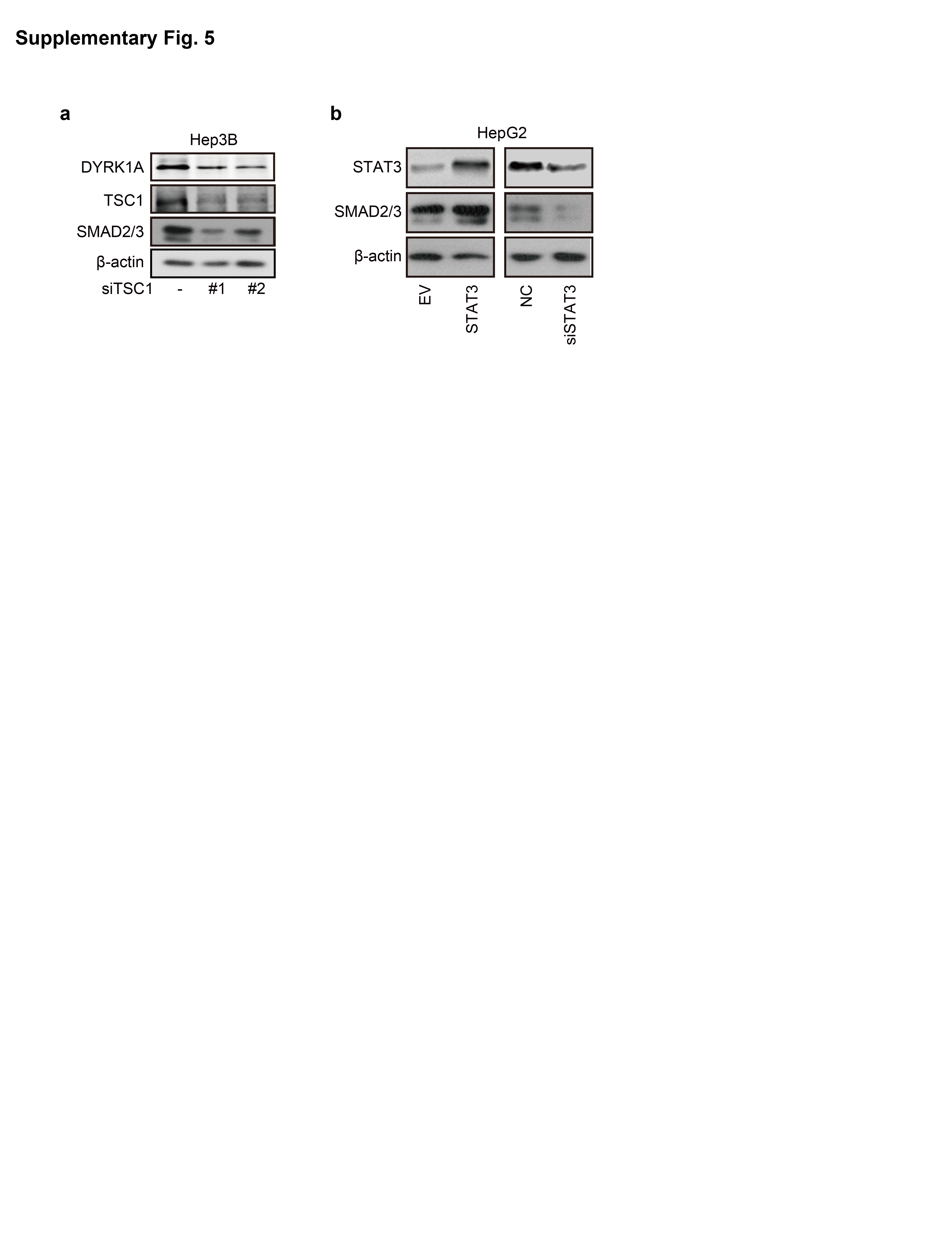

Supplement: Supplementary file 5 — Additional file 5: Figure S5. TSC1 knockdown downregulated SMAD2/3 in HCC cells. (a) HCC cells were transfected with TSC1 siRNA or control small interfering RNA for 48 h, and the expression levels of the indicated proteins were assessed by western blotting. (b) HCC cells were incubated with the STAT3 overexpression plasmid or STAT3 siRNA for 48 h, and western blot analysis was then performed. [file 12929_2022_817_MOESM5_ESM.tif]
