# Supplementary material for: DYRK1A reinforces epithelial-mesenchymal transition and metastasis of hepatocellular carcinoma via cooperatively activating STAT3 and SMAD
Source: J Biomed Sci. 2022 Jun 2;29:34. doi: 10.1186/s12929-022-00817-y (PMC9164892; doi:10.1186/s12929-022-00817-y)
Supplement: Supplementary file 6 — Additional file 6: Table S1. RNA sequencing analysis of gene expression in highly invasive and weakly invasive HepG2 cells. RNA sequencing was performed to compare gene transcription levels between P5 and P0. [file 12929_2022_817_MOESM6_ESM.pdf]

Supplementary table 1: RNA-seq analysis of gene expression in high-invasive and low-invasive HepG2 cells

TOP 20 up regulation genes					TOP 20 down regulation genes				
Gene	FPKM.High	FPKM.Low	FC	LOG2FC	Gene	FPKM.High	FPKM.Low	FC	LOG2FC
TPI1	707.50	2.62	270.13	8.08	DSG2	0.19	36.66	0.01	-7.58
DYRK1A	15.23	0.09	173.77	7.44	HIST1H2E	6.99	1261.04	0.01	-7.50
SELENOI	16.03	0.10	157.66	7.30	LASP1	0.30	52.79	0.01	-7.45
APLP2	51.51	0.33	156.22	7.29	HIST1H2A	15.97	1263.82	0.01	-6.31
CTSD	175.46	1.73	101.60	6.67	MYOF	0.20	16.14	0.01	-6.30
PAICS	108.62	1.13	95.75	6.58	TGOLN2	0.25	19.92	0.01	-6.30
ABCC4	27.69	0.33	83.59	6.39	MGAT1	0.34	25.13	0.01	-6.20
ACTB	286.22	3.62	78.96	6.30	NCAPG2	0.31	20.03	0.02	-6.02
CLUH	19.20	0.26	72.95	6.19	NUP214	0.21	13.74	0.02	-6.00
LASP1	191.45	2.71	70.67	6.14	COPS7A	0.76	48.21	0.02	-5.99
TUBB3	36.55	0.53	68.41	6.10	PDXDC1	0.25	13.97	0.02	-5.79
HDLBP	23.82	0.36	66.50	6.06	NUTF2	0.86	45.95	0.02	-5.74
NCLN	35.10	0.54	65.22	6.03	DBNDD1	0.43	21.99	0.02	-5.68
USP19	13.38	0.22	60.69	5.92	LIF	0.24	12.28	0.02	-5.65
FGFRL1	56.73	0.98	57.81	5.85	PSMG2	0.90	43.63	0.02	-5.60
ECE1	15.90	0.30	53.40	5.74	ARID1A	0.21	10.14	0.02	-5.57
SPECC1L	8.59	0.16	53.12	5.73	GSPT1	0.95	44.19	0.02	-5.54
FDFT1	21.81	0.43	50.22	5.65	SCRN1	0.76	34.12	0.02	-5.49
MDM4	6.13	0.12	49.50	5.63	DDX17	1.57	70.78	0.02	-5.49
EIF3L	33.22	0.67	49.49	5.63	HNRNPH1	0.75	33.06	0.02	-5.46
