# Supplementary material for: DYRK1A reinforces epithelial-mesenchymal transition and metastasis of hepatocellular carcinoma via cooperatively activating STAT3 and SMAD
Source: J Biomed Sci. 2022 Jun 2;29:34. doi: 10.1186/s12929-022-00817-y (PMC9164892; doi:10.1186/s12929-022-00817-y)
Supplement: Supplementary file 7 — Additional file 7: Table S2. Overrepresentation enrichment analysis of DYRK1A in clinical samples obtained from patients with hepatocellular carcinoma. [file 12929_2022_817_MOESM7_ESM.pdf]

Supplementary Table 2：Overrepresentation Enrichment Analysis of DYRK1A in HCC clinical samples

Biological process(BP)

geneSet	description	size	overlap	expect	enrichment Ratio	pValue	FDR
GO:0051056	regulation of small GTPase mediated signal transduction	316	198	123.6	1.60	0	0
GO:0007264	small GTPase mediated signal transduction	526	315	205.8	1.53	0	0
GO:0007265	Ras protein signal transduction	413	246	161.6	1.52	0	0
GO:0120031	plasma membrane bounded cell projection assembly	508	298	198.7	1.50	0	0
GO:0030031	cell projection assembly	520	302	203.4	1.48	0	0
GO:0007507	heart development	512	293	200.3	1.46	0	0
GO:0030334	regulation of cell migration	741	414	289.9	1.43	0	0
GO:0000902	cell morphogenesis	939	517	367.3	1.41	0	0
GO:0000904	cell morphogenesis involved in differentiation	683	376	267.2	1.41	0	0
GO:0007017	microtubule-based process	640	352	250.4	1.41	0	0
GO:0051270	regulation of cellular component movement	868	475	339.5	1.40	0	0
GO:0120036	plasma membrane bounded cell projection organization	1355	741	530.1	1.40	0	0
GO:2000145	regulation of cell motility	793	433	310.2	1.40	0	0
GO:0030030	cell projection organization	1387	757	542.6	1.40	0	0
GO:0035239	tube morphogenesis	774	421	302.8	1.39	0	0
GO:0032989	cellular component morphogenesis	1033	560	404.1	1.39	0	0
GO:0040012	regulation of locomotion	866	469	338.8	1.38	0	0
GO:0072359	circulatory system development	972	526	380.2	1.38	0	0
GO:0031175	neuron projection development	885	475	346.2	1.37	0	0
GO:0051960	regulation of nervous system development	821	440	321.2	1.37	0	0
GO:0035295	tube development	949	508	371.2	1.37	0	0
GO:0060284	regulation of cell development	838	445	327.8	1.36	0	0
GO:0044087	regulation of cellular component biogenesis	862	455	337.2	1.35	0	0
GO:0048666	neuron development	1005	528	393.1	1.34	0	0
GO:0051726	regulation of cell cycle	1023	537	400.2	1.34	0	0
GO:0022008	neurogenesis	1455	758	569.2	1.33	0	0
GO:0022610	biological adhesion	1300	676	508.5	1.33	0	0
GO:0007010	cytoskeleton organization	1181	614	462.0	1.33	0	0
GO:0007155	cell adhesion	1293	672	505.8	1.33	0	0
GO:0048699	generation of neurons	1364	706	533.6	1.32	0	0
GO:0016477	cell migration	1271	654	497.2	1.32	0	0
GO:0030182	neuron differentiation	1233	634	482.3	1.31	0	0
GO:0022402	cell cycle process	1137	581	444.8	1.31	0	0
GO:0006928	movement of cell or subcellular component	1815	924	710.0	1.30	0	0
GO:0007049	cell cycle	1577	802	616.9	1.30	0	0
GO:0048468	cell development	1937	984	757.7	1.30	0	0
GO:0071495	cellular response to endogenous stimulus	1271	644	497.2	1.30	0	0
GO:0031327	negative regulation of cellular biosynthetic process	1400	708	547.7	1.29	0	0
GO:0045595	regulation of cell differentiation	1575	793	616.1	1.29	0	0
GO:2000026	regulation of multicellular organismal development	1774	893	694.0	1.29	0	0

GO:0009890	negative regulation of biosynthetic process	1423	715	556.7	1.28	0	0
GO:0045893	positive regulation of transcription, DNA-templated	1430	716	559.4	1.28	0	0
GO:0048870	cell motility	1394	696	545.3	1.28	0	0
GO:0051674	localization of cell	1394	696	545.3	1.28	0	0
GO:0040011	locomotion	1603	796	627.1	1.27	0	0
GO:0045935	positive regulation of nucleobase-containing compound metabolic process	1746	859	683.0	1.26	0	0
GO:0006468	protein phosphorylation	1746	856	683.0	1.25	0	0
GO:0010628	positive regulation of gene expression	1812	883	708.8	1.25	0	0
GO:0010557	positive regulation of macromolecule biosynthetic process	1737	846	679.5	1.25	0	0
GO:1902531	regulation of intracellular signal transduction	1709	832	668.5	1.24	0	0

	Molecular function(MF)						
geneSet	description	size	overlap	expect	enrichment Ratio	pValue	FDR
GO:0005089	Rho guanyl-nucleotide exchange factor activity	75	53	28.5	1.86	9E-09	6E-07
GO:0019783	ubiquitin-like protein-specific protease activity	105	69	39.9	1.73	7E-09	5E-07
GO:0017048	Rho GTPase binding	162	102	61.6	1.66	9E-11	8E-09
GO:0005201	extracellular matrix structural constituent	151	94	57.4	1.64	1E-09	8E-08
GO:0005088	Ras guanyl-nucleotide exchange factor activity	241	145	91.6	1.58	2E-12	2E-10
GO:0005085	guanyl-nucleotide exchange factor activity	306	181	116.3	1.56	3E-14	4E-12
GO:0005096	GTPase activator activity	258	151	98.1	1.54	1E-11	1E-09
GO:0004674	protein serine/threonine kinase activity	427	249	162.3	1.53	0	0
GO:0004672	protein kinase activity	616	353	234.1	1.51	0	0
GO:0030695	GTPase regulator activity	288	165	109.5	1.51	2E-11	2E-09
GO:0017016	Ras GTPase binding	497	284	188.9	1.50	0	0
GO:0031267	small GTPase binding	514	293	195.4	1.50	0	0
GO:0003700	DNA-binding transcription factor activity	1620	922	615.7	1.50	0	0
GO:0000981	DNA-binding transcription factor activity, RNA polymerase II-specific	1525	866	579.6	1.49	0	0
GO:0003682	chromatin binding	495	281	188.1	1.49	0	0
GO:0016773	phosphotransferase activity, alcohol group as acceptor	742	414	282.0	1.47	0	0
GO:0051020	GTPase binding	599	333	227.7	1.46	0	0
GO:0140110	transcription regulator activity	1923	1065	730.9	1.46	0	0
GO:0060589	nucleoside-triphosphatase regulator activity	330	180	125.4	1.44	5E-10	4E-08
GO:0016301	kinase activity	800	436	304.1	1.43	0	0
GO:0004842	ubiquitin-protein transferase activity	380	206	144.4	1.43	6E-11	6E-09
GO:0019787	ubiquitin-like protein transferase activity	404	217	153.5	1.41	6E-11	6E-09
GO:0016772	transferase activity, transferring phosphorus-containing groups	931	484	353.8	1.37	0E+00	0E+00
GO:0016887	ATPase activity	406	211	154.3	1.37	5E-09	3E-07
GO:0019901	protein kinase binding	603	308	229.2	1.34	2E-11	2E-09
GO:0008092	cytoskeletal protein binding	887	452	337.1	1.34	4E-16	6E-14

GO:0005524	ATP binding	1380	700	524.5	1.33	0E+00	0E+00
GO:0019900	kinase binding	676	342	256.9	1.33	7E-12	7E-10
GO:0032559	adenyl ribonucleotide binding	1432	722	544.3	1.33	0E+00	0E+00
GO:0008134	transcription factor binding	622	313	236.4	1.32	1E-10	1E-08
GO:0003712	transcription coregulator activity	523	263	198.8	1.32	4E-09	3E-07
GO:0030554	adenyl nucleotide binding	1445	724	549.2	1.32	0E+00	0E+00
GO:0019904	protein domain specific binding	663	328	252.0	1.30	5E-10	4E-08
GO:0001067	regulatory region nucleic acid binding	868	429	329.9	1.30	1E-12	1E-10
GO:0044212	transcription regulatory region DNA binding	866	427	329.1	1.30	2E-12	2E-10
GO:0000976	transcription regulatory region sequence-specific DNA binding	758	373	288.1	1.29	8E-11	7E-09
GO:0003690	double-stranded DNA binding	872	428	331.4	1.29	5E-12	5E-10
GO:0035639	purine ribonucleoside triphosphate binding	1691	829	642.7	1.29	0E+00	0E+00
GO:1990837	sequence-specific double-stranded DNA binding	791	387	300.6	1.29	9E-11	7E-09
GO:0001012	RNA polymerase II regulatory region DNA binding	715	349	271.8	1.28	1E-09	8E-08
GO:0008270	zinc ion binding	759	369	288.5	1.28	7E-10	5E-08
GO:0032555	purine ribonucleotide binding	1751	851	665.5	1.28	0E+00	0E+00
GO:0032553	ribonucleotide binding	1766	857	671.2	1.28	0E+00	0E+00
GO:0017076	purine nucleotide binding	1766	855	671.2	1.27	0E+00	0E+00
GO:0000977	RNA polymerase II regulatory region sequence-specific DNA	709	343	269.5	1.27	5E-09	4E-07
GO:0008144	drug binding	1610	772	611.9	1.26	0E+00	0E+00
GO:0017111	nucleoside-triphosphatase activity	788	376	299.5	1.26	7E-09	5E-07
GO:0043565	sequence-specific DNA binding	1044	495	396.8	1.25	8E-11	7E-09
GO:0000166	nucleotide binding	1985	924	754.4	1.22	0	0
GO:1901265	nucleoside phosphate binding	1986	924	754.8	1.22	0	0

Cellular component (CC)

geneSet	description	size	overlap	expect	enrichment Ratio	pValue	FDR
GO:0005911	cell-cell junction	418	229	130.6	1.75	0	0
GO:0016604	nuclear body	665	364	207.8	1.75	0	0
GO:0005813	centrosome	444	243	138.8	1.75	0	0
GO:0031252	cell leading edge	372	203	116.3	1.75	0	0
GO:0098984	neuron to neuron synapse	320	174	100.0	1.74	0	0
GO:0044451	nucleoplasm part	970	527	303.2	1.74	0	0
GO:0044441	ciliary part	370	201	115.6	1.74	0	0
GO:0016607	nuclear speck	340	183	106.3	1.72	0	0
GO:0005815	microtubule organizing center	622	329	194.4	1.69	0	0
GO:0098794	postsynapse	579	302	181.0	1.67	0	0
GO:0005929	cilium	491	256	153.5	1.67	0	0
GO:0000785	chromatin	470	245	146.9	1.67	0	0
GO:0099568	cytoplasmic region	440	229	137.5	1.67	0	0
GO:0097060	synaptic membrane	410	212	128.1	1.65	0	0
GO:1990234	transferase complex	708	366	221.3	1.65	0	0
GO:0005694	chromosome	904	466	282.5	1.65	0	0
GO:0015630	microtubule cytoskeleton	1030	526	321.9	1.63	0	0
GO:0044427	chromosomal part	796	405	248.8	1.63	0	0
GO:0097447	dendritic tree	574	285	179.4	1.59	0	0
GO:0045202	synapse	1108	550	346.3	1.59	0	0
GO:0000228	nuclear chromosome	514	255	160.6	1.59	0	0
GO:0044463	cell projection part	1299	643	406.0	1.58	0	0
GO:0120038	plasma membrane bounded cell projection part	1299	643	406.0	1.58	0	0

GO:0030054	cell junction	1196	592	373.8	1.58	0	0
GO:0044454	nuclear chromosome part	481	238	150.3	1.58	0	0
GO:0030425	dendrite	572	283	178.8	1.58	0	0
GO:0005912	adherens junction	514	253	160.6	1.57	0	0
GO:0070161	anchoring junction	529	260	165.3	1.57	0	0
GO:0042995	cell projection	1948	957	608.8	1.57	0	0
GO:0120025	plasma membrane bounded cell projection	1880	921	587.6	1.57	0	0
GO:0044456	synapse part	885	432	276.6	1.56	0	0
GO:0030424	axon	566	276	176.9	1.56	0	0
GO:0098590	plasma membrane region	1114	542	348.2	1.56	0	0
GO:0005856	cytoskeleton	1892	913	591.3	1.54	0	0
GO:0005794	Golgi apparatus	1394	663	435.7	1.52	0	0
GO:0043005	neuron projection	1217	577	380.4	1.52	0	0
GO:0097458	neuron part	1604	758	501.3	1.51	0	0
GO:0098791	Golgi subcompartment	766	361	239.4	1.51	0	0
GO:0036477	somatodendritic compartment	781	367	244.1	1.50	0	0
GO:0000139	Golgi membrane	677	318	211.6	1.50	0	0
GO:0005887	integral component of plasma membrane	1500	703	468.8	1.50	0	0
GO:0031226	intrinsic component of plasma membrane	1572	732	491.3	1.49	0	0
GO:0044430	cytoskeletal part	1421	660	444.1	1.49	0	0
GO:0044431	Golgi apparatus part	868	402	271.3	1.48	0	0
GO:0005768	endosome	802	366	250.7	1.46	0	0
GO:1902494	catalytic complex	1248	558	390.1	1.43	0	0
GO:0099081	supramolecular polymer	831	368	259.7	1.42	3E-16	1E-14
GO:0098588	bounding membrane of organelle	1878	810	587.0	1.38	0	0
GO:0098805	whole membrane	1526	646	476.9	1.35	0	0
GO:0031984	organelle subcompartment	1514	634	473.2	1.34	0	0
