# Supplementary material for: DYRK1A reinforces epithelial-mesenchymal transition and metastasis of hepatocellular carcinoma via cooperatively activating STAT3 and SMAD
Source: J Biomed Sci. 2022 Jun 2;29:34. doi: 10.1186/s12929-022-00817-y (PMC9164892; doi:10.1186/s12929-022-00817-y)
Supplement: Supplementary file 8 — Additional file 8: Table S3. GSEA of hepatocellular carcinoma samples with low and high DYRK1A expression using the LinkedOmics platform by WikiPathways enrichment. [file 12929_2022_817_MOESM8_ESM.pdf]

Supplementary Table 3: GSEA between low- and high- DYRK1A expression HCC samples using LinkedOmics by enriching on WikiPathway

geneSet	description	enrichment Score	normalized EnrichmentScore	pValue	FDR	size	leading Edge Num
WP366	TGF-beta Signaling	0.6533513	1.8188485	0	0.003997	132	50
WP2857	Mesodermal Commitment Pathway	0.640528	1.7838839	0	0.005996	144	64
WP4331	Neovascularisation	0.7410834	1.7614212	0.002558	0.009327	37	23
WP3651	Pathways Affected in Adenoid Cystic Carcinoma	0.6871385	1.7415463	0	0.009993	60	20
WP3584	MECP2 and Associated Rett Syndrome	0.6639486	1.7121361	0	0.016389	68	27
WP3657	Hematopoietic Stem Cell Gene Regulation by GABP alpha/beta Complex	0.8103555	1.7110509	0	0.014324	20	9
WP2038	Regulation of Microtubule Cytoskeleton	0.6850123	1.6896294	0.002433	0.021986	46	22
WP4533	Transcription co-factors SKI and SKIL protein partners	0.8198331	1.6829121	0	0.022235	18	7
WP364	IL-6 signaling pathway	0.6897165	1.682824	0	0.019765	43	14
WP437	EGF/EGFR Signaling	0.6040596	1.6686122	0	0.021786	161	68
WP2261	Signaling Pathways in Glioblastoma	0.6268575	1.6628105	0	0.021804	82	36
WP710	DNA Damage Response (only ATM dependent)	0.6143269	1.6621262	0	0.019987	110	39
WP363	Wnt Signaling Pathway (Netpath)	0.6668647	1.654693	0	0.021986	51	23
WP4337	ncRNAs involved in STAT3 signaling in hepatocellular carcinoma	0.8274906	1.6474063	0	0.023984	14	6
WP2853	Endoderm Differentiation	0.5953693	1.6412635	0	0.025317	139	56
WP3298	Melatonin metabolism and effects	0.6869549	1.6407981	0	0.023984	36	11
WP4016	DNA IR-damage and cellular response via ATR	0.6241997	1.636676	0.002299	0.024572	77	25
WP3680	Association Between Physico-Chemical Features and Toxicity Associated	0.6409993	1.6300439	0	0.027204	65	21
WP313	Signaling of Hepatocyte Growth Factor Receptor	0.702724	1.6290907	0.002571	0.026299	34	13
WP1539	Angiogenesis	0.7284788	1.6284453	0	0.025383	24	14
WP2324	AGE/RAGE pathway	0.6245354	1.6276518	0	0.02465	66	27
WP51	Regulation of Actin Cytoskeleton	0.5781763	1.6259658	0	0.024348	144	37
WP4540	Pathways Regulating Hippo Signaling	0.6015148	1.6249045	0	0.023463	98	40
WP1541	Energy Metabolism	0.65798	1.6236196	0	0.023318	47	12
WP2446	Retinoblastoma Gene in Cancer	0.6153319	1.6193992	0	0.024784	86	31
WP4655	Cytosolic DNA-sensing pathway	-0.489778	-1.571137	0	0.095313	65	17
WP3963	Mevalonate pathway	-0.837901	-1.585452	0.041916	0.087093	7	5
WP134	Pentose Phosphate Metabolism	-0.830309	-1.586067	0.023952	0.090532	7	5
WP545	Complement Activation	-0.631033	-1.588629	0.020408	0.092096	21	13
WP357	Fatty Acid Biosynthesis	-0.628343	-1.597326	0.021898	0.087988	22	10

WP4224	Purine metabolism and related disorders	-0.619534	-1.608489	0.012987	0.083169	22	5
WP2359	Parkin-Ubiquitin Proteasomal System	-0.520236	-1.634646	0	0.065396	67	19
WP4290	Metabolic reprogramming in colon cancer	-0.605586	-1.690905	0	0.037298	40	14
WP4718	Cholesterol metabolism (includes both Bloch and Kandutsch-Russell	-0.572965	-1.703154	0	0.033598	44	15
WP4752	Base Excision Repair	-0.623264	-1.71359	0	0.030437	31	12
WP4792	Purine metabolism	-0.80468	-1.72358	0	0.029527	13	5
WP197	Cholesterol Biosynthesis Pathway	-0.7919	-1.742016	0.006211	0.026053	13	6
WP4313	Ferroptosis	-0.609871	-1.746297	0	0.026361	40	7
WP15	Selenium Micronutrient Network	-0.552895	-1.749062	0	0.027055	74	29
WP107	Translation Factors	-0.603975	-1.805682	0	0.012753	50	21
WP4521	Glycosylation and related congenital defects	-0.687513	-1.808643	0.008065	0.013427	25	7
WP4022	Pyrimidine metabolism	-0.577003	-1.827483	0	0.011356	83	28
WP1946	Cori Cycle	-0.812488	-1.949934	0	0.001754	17	7
WP100	Glutathione metabolism	-0.771747	-1.96583	0	0.001431	21	7
WP4396	Nonalcoholic fatty liver disease	-0.620388	-2.164443	0	0	148	57
WP183	Proteasome Degradation	-0.787261	-2.367398	0	0	62	36
WP623	Oxidative phosphorylation	-0.885722	-2.437942	0	0	37	29
WP4324	Mitochondrial complex I assembly model OXPHOS system	-0.887087	-2.649891	0	0	44	35
WP111	Electron Transport Chain (OXPHOS system in mitochondria)	-0.869946	-2.798443	0	0	73	48
WP477	Cytoplasmic Ribosomal Proteins	-0.884494	-2.806662	0	0	88	73
