# Supplementary material for: DYRK1A reinforces epithelial-mesenchymal transition and metastasis of hepatocellular carcinoma via cooperatively activating STAT3 and SMAD
Source: J Biomed Sci. 2022 Jun 2;29:34. doi: 10.1186/s12929-022-00817-y (PMC9164892; doi:10.1186/s12929-022-00817-y)
Supplement: Supplementary file 9 — Additional file 9: Table S4. GSEA of hepatocellular carcinoma samples with low and high DYRK1A expression using the LinkedOmics platform by PANTHER pathways enrichment. [file 12929_2022_817_MOESM9_ESM.pdf]

Supplementary Table 4: GSEA between low- and high- DYRK1A expression HCC samples using LinkedOmics by enriching on PantherPathway

geneSet	description	enrichment Score	normalized Enrichment Score	pValue	FDR	size	leading Edge Num
P00047	PDGF signaling pathway	0.6660469	1.8071147	0	3.16228E-16	123	52
P00018	EGF receptor signaling pathway	0.6206637	1.6877549	0	0.016623498	114	44
P00033	Insulin/IGF pathway-protein kinase B signaling	0.7059087	1.6790763	0	0.011698017	35	18
P00035	Interferon-gamma signaling pathway	0.7329686	1.6511049	0	0.019855845	27	13
P00057	Wnt signaling pathway	0.5703342	1.6369135	0	0.020317609	289	114
P00036	Interleukin signaling	0.6137191	1.6252277	0	0.021241136	77	27
P00059	p53 pathway	0.6277772	1.6241646	0	0.018998283	75	25
P00019	Endothelin signaling	0.6223175	1.6195016	0	0.018239671	75	36
P00005	Angiogenesis	0.5713996	1.6009708	0	0.020317609	156	57
P00006	Apoptosis signaling	0.5782324	1.5708006	0.002247	0.028259946	108	38
P04398	p53 pathway feedback loops 2	0.6343703	1.5604291	0.0075	0.031064112	46	20
P00021	FGF signaling pathway	0.5686484	1.5489971	0.004484	0.035863658	102	34
P00009	Axon guidance mediated by netrin	0.6806663	1.5395838	0.007812	0.038504	30	13
P00015	Circadian clock system	0.8338329	1.5341278	0.014925	0.039843622	9	5
P00045	Notch signaling pathway	0.6535572	1.5327303	0.002545	0.037803066	39	18
P00048	PI3 kinase pathway	0.6225691	1.5303725	0.010152	0.036710225	47	22
P00012	Cadherin signaling	0.5532303	1.5249667	0.00216	0.037049757	152	63
P00052	TGF-beta signaling	0.5584162	1.4910284	0.007246	0.056129959	90	34
P00025	Hedgehog signaling	0.7058694	1.4633454	0.027397	0.077770751	19	13
P04393	Ras Pathway	0.5667473	1.4581695	0.012346	0.07877691	69	26
P00053	T cell activation	0.5583836	1.4541644	0.00995	0.078455874	75	20
P00038	JAK/STAT signaling	0.7210919	1.4525526	0.04507	0.076652796	15	7
P00034	Integrin signalling	0.516717	1.4463499	0	0.078861232	166	68
P00003	Alzheimer disease-amyloid secretase pathway	0.5670847	1.4449035	0.004796	0.077268481	63	27
P06959	CCKR signaling map	0.5104554	1.4245817	0.00655	0.096637935	167	50
P02730	Asparagine and aspartate b	-0.5466	-0.914395	0.551351	0.936019625	4	3
P02755	Methylmalonyl pathway	-0.597724	-0.926547	0.57971	0.940369858	3	2
P00051	TCA cycle	-0.438272	-0.953292	0.465409	0.905421519	10	2
P02772	Pyruvate metabolism	-0.50487	-1.008843	0.445055	0.783220302	9	4
P02774	Salvage pyrimidine deoxyril	-0.685747	-1.067528	0.456522	0.664800604	3	2
P02727	Androgen/estrogene/proge	-0.55175	-1.075181	0.348837	0.678538181	9	4
P02722	Acetate utilization	-0.686355	-1.084761	0.438503	0.686620628	3	1
P00024	Glycolysis	-0.553013	-1.242446	0.143791	0.377374513	16	5
P02785	Valine biosynthesis	-0.786509	-1.244469	0.268421	0.395532549	3	2
P02748	Isoleucine biosynthesis	-0.786509	-1.244469	0.268421	0.395532549	3	2
P02776	Serine glycine biosynthesis	-0.744307	-1.263015	0.191257	0.409347088	5	1
P00049	Parkinson disease	-0.404049	-1.30018	0.028571	0.364291106	84	17
P02787	Vitamin B6 metabolism	-0.840744	-1.32126	0.131148	0.348911813	3	1
P02746	Heme biosynthesis	-0.624253	-1.349551	0.100629	0.31993018	12	6
P00058	mRNA splicing	-0.80834	-1.428764	0.101695	0.204502568	5	2
P02723	Adenine and hypoxanthine	-0.756747	-1.434715	0.131148	0.215385583	6	1
P02744	Fructose galactose metabol	-0.706411	-1.479436	0.051724	0.161634587	11	4
P02752	Mannose metabolism	-0.847521	-1.496762	0.030864	0.150868034	6	5
P00013	Cell cycle	-0.715785	-1.618858	0.012739	0.05011412	16	5
P02775	Salvage pyrimidine ribonuc	-0.773915	-1.650449	0.006849	0.044156498	12	7
P02762	Pentose phosphate pathwa	-0.868718	-1.651395	0.00578	0.052006542	8	4
P00014	Cholesterol biosynthesis	-0.829726	-1.760886	0	0.017785256	12	5

P02739	De novo pyrimidine deoxyr	-0.796932	-1.769439	0.007353	0.022895962	13	6
P02738	De novo purine biosynthesi	-0.659192	-1.784905	0	0.029437665	26	12
P02740	De novo pyrimidine ribonu	-0.844143	-1.791651	0.01227	0.056422191	10	6
