# Supplementary material for: DYRK1A reinforces epithelial-mesenchymal transition and metastasis of hepatocellular carcinoma via cooperatively activating STAT3 and SMAD
Source: J Biomed Sci. 2022 Jun 2;29:34. doi: 10.1186/s12929-022-00817-y (PMC9164892; doi:10.1186/s12929-022-00817-y)
Supplement: Supplementary file 10 — Additional file 10: Table S5. The gene lists of DYRK1A interacting and co-expressed proteins and SMAD2/3 positively co-expressed proteins. [file 12929_2022_817_MOESM10_ESM.pdf]

Supplementary Table 5: The gene lists of DYRK1A interacting and co-expressed proteins and SMAD2/3 positively co-expressed proteins.

DYRK1A interacting proteins	DYRK1A coexpressed proteins	SMAD3 coexpressed proteins	SMAD2 coexpressed proteins	25 overlapping proteins
From Literature	From UALCAN			
AARSD1	SON	SIN3A	C18orf25	AFF1
ABCF2	GABPA	RNF111	MFN1	CAMSAP1
AFF1	SYNJ1	SP1	RNF4	GBF1
AKR1A1	RNF160	ACVR1	TRIP12	NOTCH2
ALDH9A1	EPC2	YTHDC1	SMARCA5	SUPT7L
ANAPC1	BOD1L	BPTF	ROCK1	ANAPC1
ANAPC16	CTDSPL2	RYK	PIK3C3	CDC27
ANAPC2	DPP8	ARIH1	NARS	HAUS2
ANAPC4	SP3	SLTM	MOBKL1B	PHF6
ANAPC5	STAG1	HMG20A	DNAJC13	TRIM56
ANKHD1	GPATCH8	INO80	ATR	AP3B1
AP3B1	ZBTB11	BOD1L	TNPO1	DCAF7
BCAP31	YTHDC1	TCF12	PAFAH1B2	HUWE1
BDP1	PIKFYVE	TTC30B	LUZP6	PSMD5
BICD2	REV1	KDM3B	UEVLD	TRIP12
BRE	ROCK1	SMARCAL1	PRPF40A	BDP1
BUB1B	ZFP91	CLASP1	CPSF2	EPS15
C1orf226	BIRC6	ADNP	HUWE1	LRBA
CAMKK2	RBM16	DNAJC14	YWHAG	RFX7
CAMSAP1	CREB1	TTC30A	ZNF397	TSC1
CAMSAP2	REST	CDK13	KIAA1468	BICD2
CAPN2	NPAT	KIAA1267	BAZ1B	ERC1
CAT	MGA	GPATCH8	VPS4B	NF1
CCDC124	NARG2	ARID2	NAA15	RNF169
CCDC97	MORC3	MATR3	DNAJB14	WDR47
CDC23	RIF1	MAP3K1	MBD1	
CDC27	SFRS2IP	SCAND2	NCKAP1	
CFL2	CHD8	BCL2L1	FAM98B	
CNBP	AQR	ZNF646	HNRPLL	
CTTNBP2NL	LEMD3	GIGYF2	ZFP91	
DCAF7	ZBTB6	VEZF1	ATP2C1	
DCPS	DCK	EPC2	USP46	
DDHD2	KLHL28	SMARCC2	EIF4G2	
DNAJC7	EP300	ZNF142	ACTR2	
DPYSL2	ARID1B	MLLT6	TNPO3	
DYRK1A	CASP8AP2	RNF44	KIAA1632	
EAF1	EXOC5	SRCAP	CCDC132	
ELP2	SFRS15	ZC3H4	KPNA4	
EPS15	MED13L	VPS39	C14orf118	
ERC1	ZNF236	MFAP1	TRIM44	
ERCC5	HMGXB4	ERCC3	YLPM1	
EXOSC1	RSF1	NARG2	RAB10	
FAM117B	ATAD2B	C15orf44	PIK3CA	
ABRAXAS1 (FAM	SP1	WDR73	STRN	
FAM53C	C18orf25	RNF38	ACAP2	
FHL2	SETX	ATF1	ARMC8	
FN1	CREBBP	PTPN9	HIAT1	
FZR1	ARID2	MAML1	C10orf18	
GBF1	PUM2	ZNF236	SS18	
GLCCI1	RNF169	WIPF2	RRAGC	
HAUS2	INTS2	PUM2	BOD1L	
HUWE1	KIDINS220	ZFP161	GNL3L	

KMT2D	EPC1	CHD2	PPP2R5E
KRI1	ELF2	RNMT	KIF2A
LIG1	ZNF791	RAB3GAP1	DHX15
LNPEP	TSHZ1	CRAMP1L	PIAS2
LRBA	ADNP	ZBTB44	RPE
LRRC47	TRAPPC10	PUM1	SAMD8
MED18	PRPF40A	CSNK1G1	ADNP2
MLLT3	MLL2	YAP1	DPY19L1
NF1	USP25	CASC3	POLR2A
NOS1AP	PRPF4B	DDX42	RSPRY1
NOTCH2	ANKRD12	GTF3C2	QSER1
NSUN2	KIAA1267	ROD1	KPNB1
NTMT1	GIGYF2	CHST14	FOXJ3
NUP35	RALGAPB	NCOA6	G3BP2
PARN	FAM179B	C2orf67	ZNF207
PCYT1A	VEZF1	ATXN1L	WIPF2
PEX19	C22orf30	EZH1	DR1
PHB2	PHF12	FNBP1L	SLC39A6
PHF6	RNF111	BBS1	SEPT7
PLBD2	RBM12	CCDC93	CREB1
PPM1G	BCLAF1	ELF2	AQR
PPP1R2	ZNF24	ARPP19	RQCD1
PPP4R2	ZNF407	RTF1	CAPZA1
PPP6R1	ZNF510	PIP4K2B	FUBP1
PSMD5	KIAA1012	RFX1	GNA13
RAD18	ZNF136	CHTF8	BBX
RALGAPA1	NCOA6	ANAPC1	CAB39
RFX7	SOCS5	MLL5	RBM27
RNF169	USP16	CMIP	TOP1
RRM1	IREB2	CIAO1	HCFC1
RRM2	ZNF41	PHF12	C5orf51
SCRIB	KIAA1632	LOC90110	AAK1
SLC38A10	SMG1	HIAT1	GRSF1
SPATA5	C14orf106	RBM12	GART
SRPK1	ZBTB26	BAZ2A	NAA35
SUPT20H	PPP1R12A	WAC	JAK1
SUPT7L	ATXN7	SNRNP200	MEX3C
TANC1	YLPM1	GRINL1A	KIAA0494
TGM2	SMAD4	CSNK1A1	SPTY2D1
TIMELESS	PIK3CA	THUMPD1	RAB3GAP1
TOMM34	QSER1	TBC1D9	RBBP4
TONSL	NR2C2	MSL1	CLTC
TRIM56	MLL5	SMURF2	TAX1BP1
TRIP12	ZNF148	ABLIM1	ARPP19
TRMT61B	SIN3A	ZFP64	CAND1
TROAP	MSL2	TRAF3IP1	APPBP2
TSC1	ACAP2	RAB5B	PPP1R12A
TSC2	HELZ	TRIP12	ME2
TTK	ARID1A	SPG11	USP25
VPS29	ARMC8	TBC1D8	RSBN1L
WDR47	CCNT1	ZNF740	SRPK2
WDR62	GRINL1A	UBE2Z	TRRAP
WDR70	C11orf30	CD2AP	ZMYM4
XPO7	TET2	HNRNPK	TRIO
	CAB39	PIAS1	RIF1
	ZNF654	MBTD1	KIAA0226
	ALMS1	CSNK2A1	GPATCH8
	ZNF776	ZNF512	BTBD10

DCAF12	DDX6	EPC2
ZNF718	USP40	PPP1CB
UBXN7	AMMECR1L	RSF1
ASXL2	UBTF	MTF1
RNF168	ZNF500	KRR1
HLCS	REV1	ZNF24
PRDM10	DYNC1I2	ATP6V1A
PHC3	GPR107	MAPK1
DDX6	CCNT2	SF3A1
MIB1	MED1	ZNF407
LOC284441	SUPT7L	SSH1
SETD1B	TET3	NAF1
RBM25	RALGAPB	LRCH3
THRAP3	G3BP2	KIAA1012
ZDHHC17	SETD1A	AVL9
PIK3C2A	ADAM10	RNMT
CGGBP1	CTCF	TMEM209
CCNT2	C11orf57	ABCE1
CUL5	ZNF830	COPB1
RNF38	PARD6B	WDR7
SRRM1	AQR	CNOT4
PHF2	MARCH7	CLOCK
MYST4	MTMR3	ZKSCAN5
CENPC1	SMEK2	UBE3C
CDK12	INO80D	ZMYM6
WDFY1	ASXL1	VRK2
SMCHD1	ZNF407	HNRNPK
MAPK1	TP53BP1	CAP1
LRCH3	TET2	GTF3C4
ZFP161	USP34	NUP155
SF3B1	PTPN12	LOC284441
UBR1	PDCD7	HIPK3
SENP1	JAK1	DDX23
RAB3GAP1	LOC647979	THRAP3
SYNRG	MYST2	USP14
NUP54	USP3	MKLN1
SLK	C2CD3	C18orf55
TTC3	C15orf17	HNRNPU
SMARCAD1	POLI	CHD4
C12orf51	RNF41	ZNF800
RTF1	VPS33B	SDAD1
HIPK3	PRPF40A	DYNC1H1
ADNP2	ATG16L1	ATP7A
LOC144438	ANKS1A	VAPA
RBM12B	SMPD4	CAPRIN1
UBA6	LOC144438	SPTLC2
RANBP2	FAM168B	RAD50
GOLGB1	RBM12B	TBC1D15
FCHSD2	HNRPDL	RAPGEF1
KIAA0430	SART3	RBM12
G3BP2	MAP4K3	SAP130
ITSN2	RABGAP1	FBXW11
STRN	NCKAP1	HNRNPUL2
SOCS4	PRR12	SLC25A40
PCNP	NR2C2	GSPT1
MPHOSPH9	BMS1	AGFG1
RQCD1	LCMT2	TP53BP1
ZBTB1	METAP1	CSNK1A1

NUP160	STK36	CBL
RSRC2	PURB	FYTTD1
ROD1	ZNF207	RNF169
KDM5A	NPEPPS	GSTCD
C9orf102	GATAD2B	TXLNG
ASH1L	PHF20	PAPOLG
HNRNPUL2	ZNF212	TGFBRAP1
ANKRD17	ANKRD40	ESYT2
USP46	STARD7	KBTBD2
ATF2	MOCS3	ALMS1
BPTF	ZBTB6	RECQL
ZNF192	CP110	GNAI3
TOP1	HMGN4	IPO7
DIP2A	CREBBP	DPP8
CHD2	CTNND1	ASXL2
ARHGAP5	LRRC57	CDC27
ACTR2	FANCF	KIDINS220
SOS2	PRKCA	SNAPC3
ZNF641	HCFC1	CCNI
MON2	LUZP6	C2CD3
PPP2R5E	TNRC18	CDK8
MEX3C	KIAA0753	IPP
TMF1	TGFBRAP1	ACTR3
TRIP12	CSNK2A1P	UBLCP1
MLL3	CTDSPL2	VPS8
RCOR1	KCTD18	DDX6
SPTY2D1	AEBP2	CDC73
MED13	MAP4K5	EPS15
ZC3H4	IFT140	SLC30A7
RLF	ARL6IP6	C9orf129
ZNF397	SP2	SLAIN2
CLOCK	SYNRG	CAMSAP1L1
KIAA1468	NUP54	BAG5
HAUS2	TULP3	NFKB1
BAZ1B	CXorf23	CDYL
FBXO11	NFAT5	RNF141
ETV3	KIAA0240	DYM
ZDHHC20	WDFY3	FAF2
ZMYM2	RASA1	ZMYND11
RBAK	TCF20	DYRK1A
RNF41	SMARCAD1	RANBP2
LATS1	TMEM127	WHSC1L1
GTF3C4	ZNF609	LUC7L2
C9orf80	MED17	C1orf58
G2E3	ZFR	FAM120A
C14orf118	ARID4B	STAM2
BAG5	SON	ADAT1
UBE3A	AP2B1	SMAD4
DCAF17	NFKB1	CUL5
DDX46	UBE3A	TAF13
SPIN1	NPAT	KPNA6
LUC7L2	RNF214	KIAA1267
BDP1	THRAP3	NSD1
SKIL	XRN2	GIGYF2
TSTD2	TSN	ANAPC1
NCKAP1	NF1	RCHY1
PATL1	CLK3	SWAP70
TET3	PAN3	TWF1

FAM98B	ZMYM4	ZNF236
NUPL1	BCL9L	SFRS2IP
ZBTB43	WDR35	PHF6
SLC30A6	QSER1	SLC30A6
FAM168A	RUNDC1	KIAA0947
TPR	FOXJ3	PHF21A
HIVEP1	FAM179B	MIER1
KRR1	CNOT8	PTP4A2
CDKN2AIP	BAT2L1	USP42
SMEK2	SFRS2IP	TRIM56
SUZ12	RPAP1	ANKRD40
INO80	PCNP	MBD2
MATR3	CWC22	TRAF6
SSH1	UNC50	PCNP
GNAQ	UPF2	WDR47
RBM27	ARMC8	RGP1
GSTCD	RAB11A	ROD1
HCFC1	DLG1	ANKRD17
CUL3	RHOT1	TRIP11
SMURF2	KDM5A	ZNF271
ZNF317	DMTF1	ATF2
SFRS13A	RERE	SBNO1
TCF12	AFTPH	INO80
SENP6	TUG1	MATR3
MAP4K5	BBS10	ZFP161
DCAF5	JKAMP	ZBTB11
FAM126B	CAB39	CUL3
ZNF627	ZNF282	EIF2AK2
TRUB1	PARD3	NCK1
USP28	WAPAL	SOCS5
PDS5B	RNF169	GEMIN5
VPS13C	ZSWIM1	CSNK2A1P
UEVLD	ZNF384	PTPN12
DNAJC13	CUL3	SH3GLB1
SLMAP	CDON	YTHDC1
C2CD3	SEPT10	YWHAQ
ZNF264	SF3B1	UBXN7
HNRNPK	RABL2A	PWWP2A
ATP11B	KHDRBS1	COPB2
CLASP1	SMARCE1	ARFIP1
MBTD1	SPPL3	RC3H2
CRAMP1L	KIAA1632	TBK1
XRN1	DHX15	SYNRG
ATRX	WDR37	CCDC93
ARPP19	RALBP1	HAUS2
CAND1	ZNF397	POLR2B
TRA2B	NXF1	CAMSAP1
APPBP2	CAMSAP1	BICD1
ZNF616	EXOC1	NIPA2
TOPBP1	FBRS	FKTN
MFAP3	RPE	RASA1
SPEN	UBN1	SMARCAD1
RSBN1L	CTNNA1	ATRX
ZNF646	ATRX	RNF41
FAM193A	ZSCAN29	C7orf70
NF1	TAF1	AKAP10
GSK3B	GTF3C4	PIGN
ZNF304	ZNF638	ZFR

TRIO	OTUD4	MIB1
ZNF765	PPP1R12A	USP9X
STX17	ARHGAP12	DDX46
ATF1	XYLT2	UBA6
NSD1	UBE3B	FIP1L1
HNRPLL	SP3	TAOK1
C10orf119	MLL2	TWISTNB
ESCO1	RBM15B	SAMD4B
PURB	NUDT21	AP3B1
PRKD3	CDYL	BMS1
WIPF2	SPTLC1	EYA3
CAPRIN1	RGPD4	MLL5
DENND4C	YLPM1	ADAM17
GNA11	DYRK1A	CASP8
TRAF6	KDM3A	VEZF1
HAUS3	SPTBN1	MAP3K2
KLF3	ATR	ANKRD13C
SMC1A	QRICH1	PSME3
SECISBP2L	NRF1	KIAA0368
RHOT1	FAM193A	BIRC6
ZNF417	GOLGB1	RNF111
MYST3	STAM2	EXD2
SLC25A40	HNRNPU	LSG1
MAPK8	GOSR1	SLC4A1AP
KBTBD4	ZFC3H1	SMEK2
NUP153	KIAA0649	PAK2
THUMPD1	NSD1	DLG1
PDS5A	FAM200A	GTF3C3
EIF4G2	EDC3	TAF3
WAPAL	COPS2	NUP205
HP1BP3	MSL2	TMEM184C
PAPOLG	C22orf30	ZC3H15
TGFBRAP1	UBAP2L	DCK
CEP192	GGNBP2	CSDE1
RNMT	GCC2	RNASEN
CSNK2A1P	POLG	PLDN
MOBKL1B	C19orf55	KIAA1033
DHX15	KIAA0831	LUZP1
RBM26	SEPT2	CCAR1
SRCAP	TPR	DUSP18
SP2	MALT1	HMBOX1
SNAPC3	PIK3C3	API5
ZNF440	EFCAB7	INTS2
SPOPL	ZNF766	C7orf42
NCBP1	NIPBL	MYNN
ZNF180	MBD5	AEBP2
MAP3K3	HNRNPUL2	LARP1
UBN2	C7orf49	QTRTD1
VPS4B	ZNF362	TMF1
SLTM	MAPK1	UTP20
CHD6	SMNDC1	PDCD6IP
ZSCAN29	KIF5B	UBR1
DNAJC14	MAPKSP1	RCOR1
UVRAG	BTBD7	RBM45
ZFR	GSTCD	IARS
ZNF12	ATF7	CCDC55
FRYL	MTMR12	PREPL
HMG20A	SOCS5	USP28

C10orf118	C7orf42	RLF
SRRM2	IREB2	SENP1
PKNOX1	TCTN2	REST
ANKRD36BP1	RFX7	KDM3B
TRIM44	MEX3C	DENND5A
CDC42BPB	CNOT6	STX12
SART3	ASXL2	VPS13C
WHSC1L1	ATXN2	SNX6
ATR	ZNF33B	CLASP1
CDK17	UBR1	BTBD1
RBBP6	ARFIP1	FUT11
VPRBP	RSBN1	TTF2
WDR82	B4GALT5	ZCCHC4
TUBGCP4	KIAA1468	PHC3
PAN3	C4orf42	OTUD4
ZDHHC21	BAZ1B	DHX8
HNRNPU	DDX18	EIF3A
USP8	UBN2	SP3
CPSF2	CS	MFAP1
PRR12	AKAP10	EXOC5
ZNF37A	RBMX	MFAP3
CELF1	CUEDC1	NPAT
PPM1D	ZNF592	RAB21
GMCL1	ZNF12	RBM12B
ADAM10	DARS	PDCD10
ZBTB8A	DCP1A	CALU
PAFAH1B2	VPS54	RAB5C
TNKS2	BAT2	RNF214
BRMS1L	PAXIP1	SRP72
TBC1D15	PLEKHA7	USP32
RGPD3	TJP1	ABCF2
PIK3C3	WHSC2	NF1
TOPORS	TRRAP	GSK3B
C10orf18	ZMYND11	ZDHHC21
APPL1	TNS3	RPAP2
WAC	LOC220930	ARHGEF12
DLG1	CHMP1B	RNF145
CP110	LUC7L2	PIK3C2A
KIAA0586	RBBP6	PUS7L
BBX	CBX3	ENTPD4
AASDH	MAPKBP1	MED1
CPSF7	SAMD4B	MSI2
MRE11A	MBD1	YIPF5
PLDN	FAM40A	TTC37
TXLNG	PRPF4B	CEBPZ
ZNF721	C3orf17	SRRM1
CSTF2T	DHX9	DNM1L
RNF138	ZNF319	GMCL1
HIVEP2	RELA	ITSN2
HNRNPF	TGIF1	SIN3A
RFX7	DYNC2LI1	C22orf30
SMC3	RABL2B	GNA11
UBXN4	UBFD1	SOCS4
PWWP2A	RUFY2	HMGXB4
USP24	ZNF704	UBE2H
POLR2B	VPS41	MALT1
FKTN	ARF3	CDK12
FBXO34	DBR1	ZNF267

FUT11	SLC39A6	SCYL2
ZNF609	CHD8	GDAP2
ZNF543	RICTOR	SP1
AKAP10	UIMC1	MARCH6
SAMD8	CASC4	ETAA1
PTAR1	ATXN7L3	VANGL1
SFRS1	RSF1	CKAP5
OTUD4	WWC2	PHTF2
C9orf129	CDK12	APPL1
CDYL	DYNC1LI2	KDM5A
RC3H1	ZNF397OS	ANKRD52
UHRF2	C17orf71	CCDC6
ATF7IP	LEMD3	MYST3
GAPVD1	NUP160	CCNT1
TRRAP	SLC4A1AP	KBTBD4
TAOK1	ETAA1	MRE11A
ZNF33A	C18orf25	YY1
FRS2	TMEM128	AGAP1
STRN3	RSRC2	SMCR8
TM9SF3	C10orf18	MED14
C14orf43	ASH1L	ZNF317
DNAJB14	KIAA1109	CTDSPL2
C15orf29	USP46	DCAF12
RABGAP1	MARK2	SFRS13A
SMEK1	TAF3	TCF12
PUS7L	PCBP2	ZNF641
BMPR2	MORF4L1	DYRK2
ZNF800	GIGYF1	PLAA
FOXJ3	RSL24D1	SF3B1
SR140	AASDHPPT	ZNF41
NHLRC2	TAF4	CNOT6
REV3L	SSH1	SNX13
MAP3K2	ZNFX1	FAM118B
DR1	ZNF623	EIF4H
GGNBP2	CCAR1	ZMPSTE24
KIAA0947	HEATR5B	YEATS2
GCC2	TCERG1	USP24
HEATR5A	SLC24A1	PACRGL
ABL1	GRSF1	NCBP1
NAB1	SF1	SNRNP27
FBXL3	HNRNPF	PNPT1
ZNF17	ERBB3	SLK
HUWE1	DPP8	DDX18
WDR47	BAHCC1	HDHD2
PIK3R4	KLHL9	ETV3
FAM116A	ZNF627	TARDBP
ETAA1	PWWP2A	ZMYM2
INO80D	CCDC55	HARBI1
ARFGEF2	PREPL	UVRAG
KIAA1109	RBM39	TTC9C
ALG10B	MKRN1	TMEM106B
TAF3	MLL	YME1L1
ZCCHC11	YEATS2	PEX26
AFTPH	C11orf58	STIM2
SBNO1	DCAF16	DCAF17
SLC30A9	ZKSCAN5	ZC3HAV1
GTF2A1	CAD	EPRS
KIF5B	HIF1AN	ATF7IP

YY1	ZC3H11A	NSF
PHIP	FOXP1	ZFYVE26
CRTC3	PPWD1	RBBP5
BTBD7	DNAJC13	SART3
HIPK1	EP300	THAP1
MSL1	FBXO11	FAM193A
CCAR1	DNAL4	GOLGB1
ARL13B	ZNF264	STAG1
MED14	ITGA2	GPR107
LUZP6	NIPA2	OSMR
MTMR12	ARL5A	C3orf17
API5	PHC3	BMPR2
NUFIP2	CHD6	ATG4C
MYNN	ZNF543	SYNJ1
DYRK2	DDX52	SUPT7L
ATM	LRRC37A	NR2C2
NAA35	IWS1	TMEM185B
TRIM33	SFRS1	IMPACT
ARIH1	SETD1B	RUNDC1
KIAA0494	TMEM87A	NECAP1
SNX13	ADNP2	ZNF148
RSBN1	SRRM2	ADAM10
RC3H2	C17orf63	NEDD1
S100PBP	PDPK1	NPEPPS
TAF1B	DDX46	ARF3
ZNF92	UBA6	DBR1
MYST2	SRPK2	ZNF562
GAB1	BFAR	CHD8
SNX6	TRIM32	DDB1
ZNF689	RANBP2	SEC23IP
CREBZF	ZNF33A	KIAA0232
TAF1	LMBR1	SKIV2L2
CDC73	SNX33	CCDC75
GTF2H3	ATXN7L3B	FAM168A
TNRC6B	ANP32A	TMOD3
IKZF4	GSK3B	NSUN4
NFKB1	ZDHHC17	ZFP106
BAT2	POLR2A	DYNC1LI2
FEM1B	MACF1	C17orf71
PBRM1	SETD5	BAZ2A
BCOR	DCAF7	RIT1
HSPA13	PKN2	TSR1
STK4	TMEM109	SETX
ZMYND11	HNRNPL	DHX36
HIATL1	BTN2A1	RAB35
LOC100271836	YTHDF1	YTHDF2
MAPK1IP1L	TMEM185B	LRRC57
CDK13	CPSF6	GRINL1A
STAM2	PHF2	PIAS3
MAPKBP1	ADAM17	RP2
SLC12A6	GGA3	BPTF
PCNX	KIAA0174	JKAMP
C3orf17	CNNM4	HIPK1
ZNF134	ZNF337	DDX5
MDM1	DENND4C	NCOA6
GOSR1	CCDC121	C4orf41
ZMYM1	CLUAP1	CLCN3
MED1	KIAA1310	POM121C

ERCC6	RNF4	BRAF
USP47	USP42	KIAA1279
ARHGAP21	PIGG	DCLRE1B
ANAPC1	HMGXB4	UHRF1BP1
NEDD1	FAM134A	MRPL3
HNRNPH3	CREB1	KHDRBS1
ZNF292	DSP	TRAF3IP1
PTPRA	ATP2C1	MLL3
DBR1	HUWE1	WBP11
MIER1	ZNF516	SP2
USP42	ARHGEF18	UBE2Z
SEC23IP	FBXL19	GTF2I
ANKRD40	FXR1	AKAP11
SEPT7	WDR47	RALBP1
PPP1CB	DZIP3	C1orf109
UPF2	ZNHIT6	ELF2
EEA1	BCLAF1	ZNF791
WWC2	ZNF561	MYST2
SMARCC2	SPATA2	FBXO11
TBC1D12	HN1L	EXOC1
TOX4	NUDCD3	NDE1
BAZ2A	LDB1	FCF1
KIAA1328	MRE11A	DHX29
DIS3L	KIAA0406	ARL5A
GTF3C3	PTCH1	RBAK
TTC21B	TADA2A	LPP
ZNF107	CBL	YARS2
ZNF561	ADCY6	PPP1R8
DMTF1	NPTN	RPAP3
GNA13	SUPT6H	PSMD1
TRIP11	DGCR2	INCENP
CTNND1	SMCR8	SFRS1
TMEM184C	TOP1	SON
SMNDC1	ELK4	AGGF1
TMTC3	MKL2	ENOPH1
ZNF443	KBTBD2	TOPBP1
SMCR8	ZNF776	VPS26A
C4orf41	NUFIP2	CYFIP1
KBTBD2	DCAF12	MOSPD2
NEK1	IPO7	LOC646762
USP37	QTRTD1	SNAP23
WDR11	SUFU	ANKRD36BP1
C3orf63	STK38	TRIM32
SUDS3	WBP11	LCORL
KLHL9	CWC25	MED13L
PDCL	PDCL	XRN2
RBM14	EXOC7	RSRC1
LOC647979	TAF15	EIF2S1
MEF2A	PRPF40B	WDR82
RBBP4	SENP1	MTA2
RPRD2	CNOT4	BCL9L
CCNI	GON4L	ZNF765
PPM1A	EPC1	DCAF7
NDE1	RPRD2	ZMYM1
FANCM	COPS7B	DUSP11
MIER3	C3orf19	WDR35
PRKAA1	VPS13C	SRFBP1
LRIG2	TM9SF4	RLIM

PUM1	ARID1B	DDX3X
DCP1A	CNOT2	TMED4
ZNF484	FBXO34	ARHGAP21
EPS15	NEO1	RSPH3
BRD2	PRDM10	RALGAPB
MPHOSPH8	TARDBP	ESCO1
TJP1	TFCP2	PURB
RGPD4	TLK2	R3HDM1
NAA30	SDCCAG1	TAF2
ARID4A	C12orf51	NUPL1
RBBP5	LRIG2	COPS2
RSRC1	MED12	SCRN3
C1orf58	ITGB1	RNF6
QRICH1	FAM107B	EIF2C4
PARG	RPL32P3	CCDC52
GCFC1	SRC	HUS1
GK5	BAG5	AKIRIN1
POLR2A	DCAF17	FAM199X
FAM13B	RC3H1	ABL1
ARHGEF12	MFAP3	MED21
PKN2	DHX40	HMGXB3
CHD4	MGA	TPR
USP1	TGOLN2	BMP2K
CNOT6L	DDX23	ARID1A
PTPN9	VPS24	IPO8
RCHY1	GPBP1L1	EFCAB7
MAML1	LOC100271836	ZNF516
DCLRE1A	BDP1	MARCH7
CPSF6	CCDC103	ATL3
SIRT1	USP8	ZBTB1
RUFY2	ZNF304	NUP160
ZBTB34	LRRC37A2	FAM116A
ATP7A	MBTPS1	WAC
CTCF	ZNF134	HNRNPR
EIF2AK3	SMAD4	C9orf102
PHF6	CHD4	OPA1
ZNF845	KIAA0430	LIMS1
RNF6	KPNB1	PUM2
CEP350	SDAD1	AFTPH
CCDC52	ABI2	KIAA1430
SMC6	MNT	CSGALNACT2
KIAA0831	HEATR2	FAM38B
EXD2	GMCL1	EIF4G3
IPO8	ROCK1	ARL13B
LASS6	PTPRA	ATXN1L
ATP2C1	C4orf14	NACC2
MARCH7	DCTN5	NAA50
HIAT1	MUTED	NEK1
SCYL2	PHF21A	YWHAB
SAP130	ERBB2IP	HNRNPF
DSCR3	ZNF146	ZDHHC5
AP4E1	ABL1	PSEN1
NIPBL	PTPRG	C18orf10
SLC35A5	NAB1	UBXN4
ATP6V1A	HELZ	CCNK
ZNF142	NEK9	SRCAP
BAZ2B	RGPD3	DNAJC21
LRRC57	JRKL	BCL10

CSGALNACT2	ARID1A	C14orf106
CBL	ZNF280D	TAF1B
USP34	KLF3	FKBP14
FYTTD1	MAP4	ZNF410
KIAA0528	BMPR1A	ZNF92
RBM7	WDR5B	NUP54
ZNF550	ORC4L	ARID1B
ZNF567	SLU7	ZDHHC20
CWF19L2	SAP130	ADNP
AEBP2	MOBKL1A	TFCP2
SMARCA5	KIAA0495	ARL6
NAA50	KRR1	SLTM
UHRF1BP1	G3BP1	PAFAH1B1
ZNF587	GTF3C3	KIAA0196
SPG11	FUBP1	DDX52
CDC27	ANKRD17	VPS11
WDR7	ZC3H12C	C9orf80
AFF1	BBX	ZNF12
PTPN11	BAT2L2	HIP1
KIAA2026	MAGI3	ATG16L1
NXF1	SMAD7	DARS
ZNF799	CSNK1D	RTF1
CDK8	PRPF38B	SLC35E1
CBLL1	PHIP	RNF160
CNOT2	TRIP4	FKBP15
TARDBP	CRTC3	SNX12
TCF20	NAF1	DNAL1
DEK	LRCH3	NUDT21
ARL5A	MYO5C	DHX40
FUBP3	NFE2L2	TMEM165
ZCCHC4	FBXL20	ETF1
ZNF318	GPBP1	BTF3L4
POLA1	BBS4	MGA
CCDC82	ARHGAP5	CDC42BPB
PIGN	SPIN3	LMBR1
ARID4B	POM121C	NARG2
ZNF326	ATXN2L	LRRC41
ZNF592	GEMIN5	PARG
TMEM106B	GNAI3	BDP1
INCENP	LAPTM4A	PARP4
PAPD4	TMEM209	SP4
SPAST	WDR60	TUBGCP4
DHX8	ZDHHC5	DOCK7
SLAIN2	NIPA1	GMEB1
SOCS6	YWHAQ	RNF34
USP9X	KCTD10	C9orf41
CLDND1	NFRKB	PALB2
FIP1L1	FASTKD2	PDZD8
TRIM32	BIRC2	KIAA0430
USP48	SMG1	PATL1
DDX23	NAA40	RNF38
THAP6	ZNF548	PAPOLA
PCNT	RBM14	NHLRC2
SP4	TRIM52	FAM179B
LRRC58	GOLGA2	CNOT8
SETD5	LUC7L3	SOS1
DCAF7	GTF2I	LEPROT
BRD1	WDR48	CTCF

PALB2	FAM178A	AAGAB
DENND1B	C5orf15	ZNF845
ZBTB5	USP24	VPS13B
SUPT7L	ZBTB26	LARP7
NOTCH1	PDS5B	FPGT
TMEM39A	C14orf135	PIGG
EYA3	RAI1	PHF12
CNOT8	LARP4B	CRLF3
ZNF207	VPS4B	EIF3J
LIN54	TMEM43	MPHOSPH9
BAT2L1	TBL1XR1	AP2A2
PTPRK	ZCCHC4	ZBTB38
GATAD2B	MIER3	LEMD3
ZNF562	RFWD3	ZBTB6
RNF4	USP21	MOBKL1A
ERBB2IP	PIGN	DIP2B
KIAA0368	GLYR1	COPS8
IRF2	NFATC2IP	DSCR3
STAG2	GTF2IRD1	CDC42SE2
RAB14	PMS2	ZNHIT6
C17orf71	APPBP2	RHOT1
ZNF619	DPF2	NIPBL
DIP2B	ZC3H6	SLC35A5
PTPN21	HINFP	KIAA0586
MARCH6	XRCC5	UBE2D3
RGP1	DNAL1	ARID2
MTMR3	RNF20	ZNF142
DHX36	CCDC45	MARK2
KIAA2018	FAF2	RNF11
ZFX	ORC2L	ANKMY2
BAT2L2	HIATL1	KDSR
LDB1	NUMA1	GTF2A1
TUG1	CCDC97	WDR43
BBS10	TEAD1	KIF5B
KIAA0406	PARG	GNAQ
SCARB2	WDR82	TET2
C5orf51	TUBGCP4	THUMPD1
MKL2	TM9SF3	PDS5A
POLH	BICD2	BTBD7
AAK1	USP10	GIT2
RYK	RIF1	ZNF623
RECQL	BRD1	COPS4
ZNF175	LATS2	MTMR6
ZDHHC5	CUL5	NUB1
IPO7	RASAL2	XPO6
COPB1	RLIM	TRAK2
TRAF3IP1	FAM98B	TCERG1
KBTBD7	RIC8A	RHBDD1
ARF6	DOCK1	NUFIP2
LIN52	ARHGAP32	AP1G1
VEZT	SR140	UBA3
CWC25	PTPRF	LRPPRC
SHOC2	HNRNPA1L2	HIVEP2
PPP6C	PKD2	PPAT
GTF2I	TBC1D22B	TMEM48
AKAP11	ILF3	E2F3
ZNF836	CAPRIN1	TLN1
SUN1	JARID2	SPG11

ZW10	PIGS	SSRP1
VPS39	TNKS2	PRKDC
CCDC93	MBD2	PDCL
KDM3B	BIRC6	C1orf83
CAMSAP1	SKIV2L2	S100PBP
ANKRD26	MINK1	CFLAR
ELF1	GRB7	MKRN1
NIPA2	TTC5	KDELC2
KIAA0240	ANKRD11	AFG3L2
UBN1	WDFY1	ZW10
TFCP2	MBNL1	RNF168
MADD	FBXW11	EPC1
SDCCAG1	TBCK	PLEKHA8
ZBTB44	CDC42SE2	SLMAP
ZNF420	MORC2	CBLL1
ATG2B	C20orf4	ATP11B
YME1L1	ZNF24	WDFY3
CBX1	CCDC6	THUMPD3
KPNA4	CCNDBP1	BLZF1
BRPF1	ZBTB4	MCPH1
PPP3R1	HNRNPD	TMEM127
AMMECR1L	U2AF2	PLEKHA3
UBTF	SEC11A	DNAJC14
MOSPD2	SMG7	RFWD3
PAXIP1	N4BP1	MED17
TULP4	CSDE1	IWS1
FAF2	ZNF234	PUM1
SPATA13	RNASEN	PIKFYVE
ZZZ3	ABI1	SPAST
UBR7	RBM27	HNRNPA2B1
FAM73A	ROCK2	MLL2
SGK269	ZBTB11	UBTF
HERC3	BCL7A	NOL10
SAMD4B	CSRP2BP	SEC23B
RFX1	C16orf63	CCDC50
SACM1L	CEP290	LOC144438
SFRS12	PAPOLG	RNF20
MAP2K1	EIF4ENIF1	SPRED1
RBM43	INTS2	MPHOSPH8
SENP7	NCK1	USP48
SPAG9	CWF19L2	HIATL1
LCMT2	UBA3	ZZZ3
YES1	ADO	FAM73A
DOCK1	ZBTB7A	GMFB
PTTG1IP	YWHAB	LOC100271836
VKORC1L1	MLH3	CTDP1
SETD1A	CASP2	C14orf43
DNM1L	FGD6	CBX3
ZNF197	UBXN7	MAPKBP1
FBXW2	CEP68	GNB1
ANKRD13C	KIDINS220	FAM102B
ZEB1	ZNF507	TRIM37
RICTOR	ZNF143	CCNT2
NPHP3	KDM1B	GOSR1
C11orf57	INADL	GPN1
CCDC75	SGPL1	HIF1A
BRD3	TRIM13	SELT
MALT1	SNRNP27	ANKRD12

ZNF397OS	POLR2B	ZNF37A
RAPGEF1	MEX3D	GTF3C1
IPMK	MAST4	MFHAS1
PELI1	STAU1	HNRNPL
SMU1	ZMYM2	USP1
CKAP5	TNPO2	USP47
SGPP1	C5orf41	KIAA0317
HNRNPR	PGBD4	ATF1
OPA1	ZNF248	AHCYL1
SF3A1	CHMP5	RIC8A
AGFG1	ZNF260	PTPN9
CAMK2D	RAD51L3	YES1
PPIG	WWTR1	TFAM
FOXJ2	ACVR2A	GTF2H1
TAF4	ZNF805	MAML1
ALG10	PAPD4	CPSF6
ZNF557	HNRNPA2B1	KCMF1
GIT2	DHX8	MYST4
LUZP1	CAMSAP1L1	MYH9
MYO5C	RNPS1	PRKD3
NCK1	RBM25	VPS41
POM121C	KDM5C	ZNF639
PTPN12	CYFIP1	M6PR
ZBTB2	USP25	RALA
MOBKL3	EIF4B	GBF1
GART	BTF3L4	CUL2
MTX3	GAPVD1	FKBP9
YWHAB	SLC11A2	GATAD2B
ZNF354B	SNAP23	GCC2
KHDRBS1	CDC42BPB	RICTOR
PSEN1	ZZZ3	UIMC1
METTL14	LRRC41	ZFYVE9
PRMT10	CCDC43	USP15
ZNF143	GPATCH2	CEP63
WDR37	TRIM4	KLF3
RBM39	PKP4	HIVEP1
KDELC2	ZMYM1	SMARCC2
CNOT4	FBXO21	ZNF434
BTRC	BMPR2	MINK1
CD2AP	PALB2	EFTUD2
ANUBL1	PIK3CA	LASS6
GOPC	SFRS12	ZNF397OS
CLTC	RBM43	RAB23
NUP98	CEP170	MTMR9
RMI1	LOC550112	UTP3
DOCK9	HNRPLL	WRN
ANKRD50	NR2F2	TMX1
ZC3H11A	SRRM1	KCTD7
ALKBH8	TFAP4	STXBP3
SNRNP27	TRA2A	POLK
EXOC1	RDH14	PAIP1
TMX3	AP1AR	BCLAF1
ZFYVE16	ZBTB34	FAM114A2
SPRED2	ZNF148	CREBBP
C5orf41	LSM14A	ZBTB4
TUBGCP3	LEPROT	MAPK8
MED17	DIS3L2	ACVR1
NUP155	PTPRK	USO1

SERTAD2	ZFP91	GCN1L1
NAA15	MED28	SMNDC1
HECTD1	JMJD1C	NOM1
UBE3B	AAGAB	C10orf46
EIF3A	KIAA0947	SPATS2
DCAF10	DDB1	C11orf30
BAG4	STK25	LOC441208
XRCC5	CTDSP2	PIP4K2C
TIA1	SAFB2	CYLD
ZNF500	UBE2H	SUPT6H
ZNF441	RAD50	ETV6
RAB21	HCG18	MTMR12
FAM168B	TMOD3	KLHL28
UBE4A	EXD2	MAN1A2
HNRPDL	MTF1	WDR3
CYP20A1	PYGO2	LARS
ZFYVE26	PPP1R2	CSTF2T
ZNF708	DIP2B	MOBKL3
BACH1	TOX4	ZBTB7A
ORC2L	PUS7	MAP4K5
WHSC1	NCBP2	MTX3
SRP72	PATZ1	CHCHD3
GMFB	SMU1	ARAP2
MYSM1	CDKN2AIP	RNF44
TNPO1	ZFHX3	MMGT1
ZMYM4	CRKL	TRIM33
MBD1	DHX36	VTI1A
BCL9L	STXBP3	TTC30B
ZFC3H1	LRBA	VEZT
MYCBP2	LOC100129550	NAA40
BAZ1A	INTS12	ZNF664
KPNB1	ALG10B	RBM14
TMED4	SF3A1	DNM2
SDAD1	BRD7	PPP6C
FAM111A	ATF2	ZC3H4
ALS2CR8	C9orf82	MRPL42
APC	AGFG1	PTPN11
RASSF3	ZC3H7A	PCYT1A
ZNF639	NUP153	STARD3NL
COPS2	COIL	CD2AP
MED23	RAB11FIP2	ZBTB26
SPTLC2	PRDM4	PDS5B
GBF1	YY1	LMTK2
DDB1	PLDN	NUP98
ZFYVE9	PDS5A	SCFD2
USP15	AGAP1	C3orf38
CWC22	EIF4G2	EP300
RAB28	CASK	C7orf60
JRKL	CCDC115	TSHZ1
BMPR1A	SEC22A	OR2A7
TTC5	GTF2IP1	SLC35F5
MOBKL1A	RBM7	CTNNA1
SLC4A1AP	ZNF136	TMX3
FBXW11	TBC1D3	TTL
TMX1	ZSWIM4	HEATR6
POLK	C4orf41	TCF20
FUBP1	ZNF317	ZNF689
MARK2	ZNF2	DDX20

FAM175B	ALMS1	CCT6A
SMG7	ZBTB22	C5orf41
USO1	ZNF574	TMEM206
SNRNP200	COMMD2	POLA1
TADA2A	TEX261	ZSCAN29
PRPF38B	CSTF2T	MADD
CSNK1A1	LARP1	SNX27
SS18	LRPPRC	PHF14
MPZL3	MTX3	PHF20L1
ADCY6	CTDSP1	PNO1
CDK19	UTP20	GTF2H3
NPTN	DHX35	TRA2B
FRG1B	RAB3IP	C12orf4
DDX5	ASAP2	DPF2
HEATR5B	MLL3	FRYL
FBXL20	RBM26	AP2B1
MAN1A2	TRUB1	PAPD4
ERCC4	TSC1	PARP8
NCOA1	CFLAR	IQCE
ATXN1L	ZNF749	IKZF4
CLCN3	BTRC	ZNF616
ESYT2	SPOPL	SETD1B
ZNF2	ASB3	SLC7A1
ZNF225	ZFYVE20	XRCC5
KIAA1279	LOC400657	RBM25
SRBD1	INTS3	FBXO28
APAF1	C1orf109	ANKS1A
UBA3	DENND5A	CRYBG3
CCDC111	METTL9	NSUN3
DCUN1D1	C5orf43	DYNC1I2
QTRTD1	HAUS2	UBE4A
METTL4	SNX6	GAPVD1
ZNF324B	KIN	SLC36A1
SMARCE1	ANKRD26	MTMR2
YWHAQ	ZNF3	UBR7
KIF2A	DGCR8	MON1B
COPB2	POLR1B	TEAD1
ZNF664	FCF1	WHSC1
SMAD2	C20orf194	GLG1
GOLGA2	ZMIZ2	PTPN4
CCDC55	UBAP2	MAPK1IP1L
C1orf124	C7orf70	CDK13
MLL	PTAR1	KIF3A
YEATS2	KIAA0247	TBC1D5
ZNF527	RBM18	KIAA1797
ZNF281	TTC9C	COL4A3BP
ZFYVE20	SLC7A6	FCHSD2
ISG20L2	EPS8	XPR1
C14orf135	YME1L1	PKN2
C3orf38	FRYL	DENND1B
ZMYM6	CCDC132	ZNF319
KDM6A	TRAPPC2	SLC30A5
RPE	LRRC37B	CORO1C
FCF1	RSL1D1	FAM104A
RASA1	PBX2	VKORC1L1
TLK2	ZNF235	TRNT1
ZNF445	ZNF224	APC
MLLT4	TIA1	PACSIN2

POLI	VPS26A	BAT2L1
VPS8	RCOR3	JOSD1
RBM18	LOC646762	RPA1
RAD51L3	TECPR2	NIPAL3
MCM3AP	BCOR	UBTD2
WDR20	RSBN1L	NAT10
DIDO1	MTMR2	DIMT1L
CCDC132	TCF3	PHF20
HNRNPA2B1	MED13L	UBE2W
USP12	NOL8	C11orf57
MFAP1	TJAP1	RBM16
ZNF224	SET	SCLT1
ANKS1A	TAOK2	BRMS1L
SEC23B	BTBD9	PTGFRN
SPTLC1	VPRBP	HAUS3
EVI5	ZNF84	GTF2E1
NEK7	STAG1	URB1
ZKSCAN4	PFDN1	ORC4L
ZCCHC8	MKS1	WDFY1
RNF214	DOCK7	FXR1
VHL	TRIO	FOSL2
SFRS14	ABL2	PTPN1
USP32	SLC12A6	SLU7
C17orf85	ZMYND8	CHM
ZNF84	TMUB2	PUS7
ADAT1	ZRANB2	RAB11A
USP39	ISY1	WNK1
GMEB1	TTF1	AP4E1
MYO6	GPN1	BBS7
C9orf41	ZBTB5	METTL2B
PMS2CL	POLDIP3	CP110
PEX13	AP3B1	ASH1L
RRP1B	C17orf80	DPY19L3
PDZD8	IMPACT	PIGK
PNN	CEBPZ	INTS12
DDX3X	LOC728643	KIAA1109
MUDENG	ALS2CR8	SUZ12
UTRN	PHF15	ZCCHC11
KIAA0317	VGLL4	CTNND1
RG9MTD3	ZBED5	SNRNP200
CEP110	ITSN2	RAB18
GGA3	SFRS3	RBM28
DHX33	CIR1	C8orf37
SFRS3	SPTLC2	UHMK1
CLASP2	PAFAH1B2	AASDHPPT
PRKRIR	GBF1	KIAA0406
NIN	CNP	SCARB2
ZNF436	POMT2	COPG
EIF2C4	PSME3	MSH2
TAB2	PDGFC	ZNFX1
BTBD10	FAM49B	ZNF384
TBCCD1	MIER1	ATP6V0A2
CRLF3	ZNF687	ATG3
ZC3H14	ZHX2	PPP4R1
RBMXL1	LOC493754	PPP5C
EIF4E	NCL	RYK
CEP63	IRF2	COG6
SKI	KTN1	NAPG

KLHL20	C5orf54	TSGA14
AP2A2	TOPORS	COMMD2
ZNF434	ZNF461	TEX261
SPIN4	SFRS2	ADO
ZNF267	UTP3	SNX2
KCTD20	RHBDF2	ATM
C11orf46	BCDIN3D	MFSD1
CRKL	FAM48A	KLHL12
PIGK	RBM15	ANKRD5
ZNF493	PCGF3	NIPA1
RANBP9	TTC21B	METTL4
KIAA1430	PRPF39	CASP2
ZC3H7A	BAZ2B	FAM161B
UHMK1	ARL6IP5	FNBP1L
NR3C1	GSPT1	ARF6
DIS3	KBTBD4	ARIH1
UBE2G2	HKR1	TMEM170A
FNBP4	ZCCHC11	FTSJD1
NAF1	RGPD6	ZNF627
PVRL3	DCK	TRUB1
ETNK1	PIAS3	COPG2
PRKCA	ZNF343	GOLGA2
CCDC66	SPATS2	RSBN1
MTMR6	ZNF618	ZNF143
RAB2B	SS18	GDE1
APPL2	PCF11	WDR37
TCERG1	ZNF526	MEF2A
TTC33	MGEA5	KIAA2026
PKD1	ZNF398	BRAP
CRYZL1	ATF6B	DVL3
C7orf42	LOC642852	FGFR1OP2
TMEM209	SNX25	TM2D1
ADO	KIAA1012	ATE1
KHSRP	SVIL	LARP4B
ZFP64	CEP164	SMARCAL1
CASP2	PPP4R1	USP3
RAB5B	SLC22A5	MAP3K3
WBP11	SCAMP4	ACBD3
POFUT2	BAHD1	DDHD2
BIRC2	ESYT2	UBN1
NAA40	ACTR2	POLR1B
ARFIP1	C17orf103	C20orf194
ZNF782	RBM33	STAMBP
PPIP5K2	ZBTB45	FLII
TBK1	USP37	CSNK2A1
GON4L	WDR11	POLI
DENND5A	C3orf63	TRAPPC10
ZKSCAN5	CIC	LATS1
TADA2B	CBX5	PRMT3
IPP	FAM161B	CUEDC1
HDHD2	IFT52	ZNF326
CXorf23	TRIM33	DPAGT1
ZMYM5	ARF6	PRKAG1
FBXO3	SMC3	RAB7A
PIAS1	POGZ	EPS8
MFN1	KIAA0494	MED12
CSNK2A1	VEZT	CBX1
THAP9	ZNF514	TNRC6B

LPP	RCOR1	BRPF1
SERINC3	TMED8	HMG20A
ZNF260	BCL10	C10orf118
NFATC2IP	PPIP5K1	DDX1
SFRS18	ZNF836	WDR45L
AGGF1	CCNG2	RUFY3
PEX26	ZMYM6	BAT2
STIM2	PARN	KDM5C
BUD13	SLK	PDPR
MAP3K7	OFD1	PKNOX1
NUDT21	FAM175A	ERCC3
B3GNT2	FKTN	HSPA13
RNF141	C12orf41	XPNPEP1
VPS26A	WDR92	STK4
KDM5C	TTL	OXSR1
PLEKHA7	HEATR6	VPS24
LOC651250	STAMBP	THAP6
SRPK2	RBAK	CAST
NUMA1	SMAD6	DNAJC10
NAA25	YPEL2	ZNF646
KDM3A	SPATA5L1	CCDC43
LMBR1	NGRN	PSMD11
TEAD1	PAFAH1B1	C17orf85
MKLN1	PPP1R8	EEF2K
C15orf44	ANKRD42	POLR3A
GPR107	VPS11	OGFOD1
TBC1D5	C16orf88	BICD2
BCL2L2	CREBZF	USP39
LATS2	VPS8	NRD1
MSI2	CCDC142	UTP15
HNRNPL	THAP2	USP8
BTN2A1	UBE2R2	LRRC58
ZNF182	SNX27	SLC12A6
C17orf80	SFT2D3	MDM1
RSPH3	DUT	ARMC10
SPOP	CDC73	SEH1L
ABHD13	MIB1	CXorf38
KIAA0226	WDR20	DHX9
AP1AR	CBX1	ZFC3H1
ZNF585B	ATXN7	CYB5RL
SNX4	NCOA5	UBQLN1
PIP5K1A	BRD2	PRR12
TINF2	AGBL5	C13orf23
BRWD3	NKIRAS2	CEP170
TMEM194A	SPRED1	CELF1
AKAP13	SPIN1	PRKACB
CCDC121	FAM131A	TRA2A
ZNF828	OGT	PHTF1
KIAA1310	FAM108C1	TMEM167B
PHF21A	STK35	HEATR2
ELMO2	OXSR1	RUFY2
TOB2	MKL1	NRAS
ZDHHC6	WHSC1L1	ZBTB8A
ZNF146	COG1	EIF2AK3
RAB5A	C1orf58	PLEKHM1
KIAA0232	GLG1	PTPRA
SKIV2L2	NFYA	BRWD3
MED21	FAM120A	DENND4C

DOPEY1	PTPN4	LMAN1
NEK4	POLR3A	ZEB1
EFCAB7	RSPRY1	SLC25A46
MTMR9	ZNF765	CIAO1
MBNL1	FAM171A1	STK17A
KCTD7	KIAA1217	SEC14L1
FAM48A	CGGBP1	CXorf56
LIMS1	C15orf29	ZNF146
ZC3H12C	DALRD3	CASC4
POLD3	KLF7	TNKS2
GCN1L1	FRMD8	C9orf30
NOM1	ZNF37A	TBCCD1
CHUK	YEATS4	EIF4E
ZNF234	ZNF182	SNX18
RNASEN	TASP1	SEPT2
JKAMP	MAPRE1	NCL
PCF11	NBR1	ADAR
C14orf4	BRD8	IRF2
TMEM30A	RG9MTD3	RAB28
TIAL1	SPOP	MSN
LOC642852	TMEM167B	SMC1A
EXOC2	MARCH8	PPHLN1
ZNF384	CASP8	RNF19B
PDCD7	PIP5K1A	ANKRD11
NAA16	FAM53C	KCTD20
CDC42SE1	C1orf77	SMU1
PLAA	HNRNPH3	KIAA1328
SF1	ANKRD13C	RSRC2
GNL3L	SLC30A6	ATMIN
TNPO3	UBTD2	SGPP1
JAK1	CENPC1	G3BP1
DHX35	VPS13B	HMGN4
SLC33A1	CCNY	ANKHD1-EIF4EBP3
PPIL4	CCDC52	SMCHD1
POGZ	BTBD10	EIF2C3
ARL6IP6	RBMXL1	CCNDBP1
VTI1A	EIF4E	POT1
TTC30B	TACC1	U2AF2
ZNF548	PPP1CB	RNF216
ZNF268	MED15	BAZ2B
EHMT1	KDM2B	RBBP8
ZNF507	HAUS3	NUP107
H2AFV	AP2A2	DNTTIP2
EIF4H	ZNF17	PCBP2
ZNF749	NSUN4	NBN
CCNG2	GMPR2	FOXJ2
SMYD4	AMBRA1	TMTC3
LNX2	SMARCD1	STRAP
UBE3C	ATAD2B	FANCF
FCHO2	VIPAR	CBFB
PPWD1	CD58	ABI1
USP3	TNKS	MAPKSP1
SCYL3	KIF3B	PGAM4
C4orf42	ZMYM3	WAPAL
ACBD3	RBM22	HP1BP3
PSMD5	KCTD7	KLHL7
MAGI1	KIAA1328	HEATR5B
TCHP	STRN4	RBM7

WDR92	HDGF	POLR1A
PURA	SETX	NOTCH2
TMEM50B	INPP5E	ELK4
NUMB	TMEM87B	SMURF2
PMS1	FRS3	C13orf31
TNPO2	COBRA1	APPL2
KIAA1712	DAZAP2	C12orf5
DDX52	FAM175B	PRPF8
VPS11	WHAMM	RPRD1A
SMC2	RAB18	TRMT61B
IWS1	TTLL5	SEPT10
PIP4K2B	GEMIN8	USP16
CAMSAP1L1	CPSF7	CDC42SE1
SFRS4	PPIG	SRBD1
ZNF772	BTBD2	RNF138
ELAVL1	HNRNPM	HEATR1
FBXO38	LOC441208	SF1
DNAL1	CTDSPL	GPD2
CCDC50	ZNF443	SUDS3
PHF3	DDX5	RFX7
HAUS6	EXOC3	PDE12
RNF20	DUSP18	NOC3L
ERCC3	TXLNG	KLHL9
OXSR1	APPL2	KCTD10
RAD23B	TRAK2	RBM26
NRF1	TGS1	VPS53
FAM120A	CEP192	BIRC2
EEF2K	SOX4	SMG1
POLR3A	UBE2I	PIP5K1C
CDK2	LARS	BPGM
RSPRY1	MOBKL3	PPIP5K2
RAB10	PTBP1	KDM1B
RNF34	HEATR1	C1orf124
TRIM37	GNL3L	IDE
UACA	UHRF1BP1	SPOPL
DHX9	SUMO2	CCNG2
C21orf91	TNPO3	ISG20L2
KIAA0649	ZNF587	SEC22B
YTHDF1	ZNF324B	IFT81
FAM76B	DCAF5	TULP3
DYNC1H1	TAB1	PSMD12
PLEKHM1P	NIF3L1	MCART1
IMPACT	MAP2K7	WDR92
BMS1	PPP6C	TBL1XR1
ALS2	REST	PRDM10
TFAM	PACRGL	ELOVL1
TBCEL	RBBP4	TLK2
ZNF615	C10orf88	CHMP5
ADAM17	DVL3	ZNF609
ZNF704	MBD6	TCP11L1
SWAP70	ACTR1B	DENND4A
NRIP1	PAPSS1	AP3M1
CASC3	ZNF791	GLYR1
CASD1	UBE2Q1	NFATC2IP
GZF1	SLMAP	RBM18
NPEPPS	MAP3K3	TNFAIP2
SBF2	TSHZ1	LRIG2
CLINT1	NUP133	G2E3

RPA1	TMX3	ZNF484
UBTD2	MFN1	ATXN7
IPO9	ZBTB10	PPP3R1
PHF20	RAB22A	RAB3GAP2
SAFB2	CANT1	CSNK1G1
SNX18	C2orf60	MGC2752
NEK9	YARS2	BAG4
KDM2B	UBLCP1	UBE3A
AMBRA1	ZNF20	HSPA4L
ZFP106	MADD	PAXIP1
DYNC1LI2	GABPA	ARPC2
HACE1	HNRNPC	ANKFY1
MTF1	NFIX	HNRNPH2
ZNF516	TSG101	TTC30A
ZNF461	RARG	FAM98A
RIT1	DTWD1	FRS2
PAK2	SMAD5	SGK269
TRAPPC6B	PIKFYVE	CDK17
PRPF39	CAND1	QRICH1
HNRNPD	TRA2B	SET
ATXN1	DIDO1	TWSG1
PIAS3	ZNF767	KDM4A
RALGAPA2	ENOPH1	TM9SF3
DENND4B	BRPF1	SKIL
RP2	PPP3R1	FAM13B
ABI1	IQCE	SLC37A3
ZNF674	KIAA0182	EXT2
LOC441208	DNAJC27	SNIP1
SUPT6H	C14orf118	PRPF4B
CAPN7	BUD13	CGGBP1
EIF4G3	METTL13	C15orf29
ROCK2	CTTNBP2NL	ZNF134
C4orf29	SF3B3	ELMOD2
AKAP2	ERC1	GTPBP8
ATP6V0A2	NRP1	GRB10
RRAGC	CCDC50	GCC1
CDC40	CYTH2	FRMD8
CEP290	PKNOX1	GNPDA2
SPIN3	ZNF597	RELA
ZNF675	ZNF451	IER3IP1
KIAA1530	STK4	MAP2K1
PRKCI	RAB5C	ECD
DCLRE1B	THAP6	TET3
GRSF1	VHL	PPP3CA
INVS	LZTFL1	ABI2
TNS1	SP4	SLC7A6OS
EZH1	MAPK1IP1L	SF3B2
CIC	ARHGAP29	ECT2
TLN1	JUP	C10orf119
CNOT6	DNAJB14	DDX31
PDCD6IP	ARHGEF12	PJA1
LINS1	TBC1D5	SETD1A
PTPLB	MASTL	TECPR1
RAPGEF2	POGK	RASSF3
TRAK1	SELT	ACTL6A
RALBP1	MLLT10	ZNF704
KIAA1958	YIPF5	CMIP
C11orf58	RNF19A	DHX33

ZNF410	TMED4	CASD1
LOC400657	PISD	ITGAV
ZNF558	CNOT6L	CWC27
BRAP	MTMR1	TXNL1
RIPK1	CELF1	UBAP2L
GXYLT1	HPS4	GGNBP2
ATE1	DSTYK	BECN1
QKI	PTTG1IP	ZNF436
TANK	TFAM	DCTN5
CCDC14	DDX31	FAM119B
SLC35F5	ESCO1	ERBB2IP
WDFY3	NUAK2	C19orf55
RNF146	ZBTB3	AGPS
HEATR6	SLC39A1	CUL4B
YPEL2	MRPL30	GRPEL2
ZNF248	PRKD3	RBBP7
PAFAH1B1	ITGAV	DDX21
ZNF8	HNRNPUL1	UNC50
PPP1R8	ZNF621	MBP
RPAP3	ZNF292	RGPD3
ZNF518A	LAMC1	MTX2
RFWD3	MTPAP	ZNF17
AP3M1	SPATA5	SMARCD1
GLYR1	PRPF38A	STAG2
RBMX	SCAMP2	CMTM1
ATG16L1	RALGAPA1	ERMP1
TMEM168	BECN1	ARHGEF18
FXC1	STK17A	GTF3C2
CBFA2T2	SEC23IP	MBNL1
MED12	SFRS6	ZNF766
ZC3H6	RAB5A	ANKIB1
RBM9	TRIM56	MBD5
LOC653501	SEPT7	PPP1R2P3
SLC5A3	HNRNPH1	LRBA
ME2	EXOC4	C7orf49
RAB3GAP2	TRAF6	ZNF561
DNAJC27	IPPK	IKBKB
ZNF606	RBM16	PHACTR2
FBXO28	RABL5	ARFGAP3
ERC1	STX6	SMG7
CYFIP1	BRD3	SYPL1
PDPK1	SAFB	SLC30A9
YAP1	LBR	REEP3
DPY19L1	SMYD5	MAP3K1
ING3	YY1AP1	CRTC3
GPATCH2	TCTN1	ZNF654
NR1D2	MPZL1	IRAK4
PTPN4	DENND5B	C2orf49
SETBP1	PELI1	EXOC2
SNRNP48	RQCD1	SNW1
C16orf72	PTPN1	URB2
BICD2	DHX57	DCTN4
BMI1	FAM116A	MKL2
WRB	SECISBP2L	NCOA1
TPP2	C15orf57	ARHGAP5
BCL9	ASB6	PPP2CA
YIPF5	STX16	KCTD18
RLIM	ATMIN	IREB2

KPNA6	WDSUB1	EZH1
C13orf23	USP7	DCUN1D1
PAPOLA	EFHC1	RAB12
EP400	PPP1R2P3	CDV3
SIKE1	MYST3	CBX5
ARHGAP32	DNMT3A	SMARCE1
FAM200A	DNTTIP2	CLCC1
TRA2A	NUP214	SMC3
MPP5	NOM1	CCT8
MTMR15	KIAA1430	ZZEF1
PIAS4	CDK6	RAB5B
ZBTB3	TUFT1	BRCC3
TAF2	DENND4B	EIF2B2
TROVE2	MLLT1	FASTKD2
GOLIM4	FNBP4	SYNCRIP
CUL2	RNF121	DDX10
MUTED	ZBTB49	MTIF2
ZNF383	PTCD3	INADL
VPS13B	DIP2A	PPIP5K1
LMAN1	ZNF510	DDX47
CHD9	MFF	RAI1
TAOK3	COPS4	CAD
LARP7	RHBDF1	ESF1
POLG	MED14	ANKRD50
CASC4	TLK1	FBXO18
TRIM56	KLHL28	CAPN1
UBE2J1	ZNF710	PSMD5
MBTPS2	NOTCH2	CHMP2B
TMOD3	ANKMY1	ELF1
KTN1	UNC119B	ZNF480
STARD7	ZNF263	KIF1B
SMAD3	PAK4	CNOT2
PRKAR2A	ZNF567	FBXO34
TMPO	WDR3	TCHP
TNKS	TBC1D23	SEC22C
DENND5B	ZNF416	USP33
NUP50	SMARCC1	CRAMP1L
KIF3B	NAPG	RAB22A
SFRS2	SFRS13A	MIER3
CHM	ZNF721	ZNF8
GDAP2	MYNN	ZNF543
KIAA0495	DYNLL2	RNF2
ZNF766	SMARCA5	WDR36
FIGNL1	PLS1	C16orf88
CEP76	IFNGR2	GABPA
TMEM128	SFRS8	KIAA0247
ZFHX3	CCDC111	TAF1
KLHL24	SUDS3	NOL9
RBM15	ASB7	SLC38A1
PCGF3	TMF1	IFT57
UBR3	ZNF785	WASF2
PPP1R2P3	ALCAM	TNFRSF10A
ANKRD52	KIF2A	WDR20
CCDC6	HEXIM1	HSPA8
EIF2C3	PDCD6IP	CNOT7
DPY19L3	C11orf61	C12orf32
KIAA1841	C1orf83	VPS35
RERE	RGL2	GOSR2

ANKHD1	PIGC	ATP10D
N4BP1	AKAP11	LOC441089
C10orf46	C14orf106	SRRM2
COIL	RLF	SF3B3
PER2	TEAD3	SPTLC1
ZNF618	ZNF92	SOCS6
C5orf44	RAD52	FAM168B
MSH2	ZNF644	REV1
ZNF770	ZNF329	TECPR2
ZBTB49	RNF220	MAN2A1
WDR89	UEVLD	TJP1
ZNF623	PIK3CB	SPIN1
MFSD8	VRK2	ZNF597
ATF7	TANK	TGOLN2
AHNAK	FBXO3	ORC2L
BCL7A	THUMPD3	NAA25
KAT5	NLK	KDM3A
AKAP8	C2orf68	LOC285033
GNAI3	SH3RF1	PCNT
SEPT10	PMS1	ING3
ATP11A	THAP9	SFRS14
COMMD2	ZNF445	VPRBP
KCTD18	COG7	RALB
MARCH5	ACTR3	TRIM4
DYNLL2	LNPEP	PAN3
ZBTB39	RCC2	PPM1G
ARAP2	PLEKHA3	BMI1
ANKRD5	ATN1	TSTD2
ZNF613	OSGEPL1	MAEA
FAM161B	MTA3	ZNF304
UTP20	MDM4	RAP1A
FNBP1L	CBFA2T2	ABL2
KIAA1009	C12orf32	MSL3
ZZEF1	FLJ45340	POGK
PROX1	KPNA4	CDC42
TEP1	C9orf129	SETD5
ZNF564	C2orf43	CREB3L2
KDM1B	IKZF4	PRPF4
PREPL	ZNF616	TYW3
BCL10	C10orf118	CHTF8
SSH2	RIC8B	SLC9A6
ZFP30	FBXO38	BTN2A1
C1orf109	FBXO28	ZNF182
SMCR7L	TRMT5	WDR12
DVL3	ZNF652	LCMT2
PIAS2	MED20	METAP1
C5orf43	C22orf46	SIKE1
LARP4B	UHRF2	GFPT1
FRMD4B	LOC651250	NOP14
PRDM2	PDCD10	INPP4A
DDX18	EIF2C1	ARL8B
MCART1	EDC4	AP1AR
BTBD1	TRIM44	THOC2
USP33	RUSC2	CASC3
RAB22A	LIG3	ZNF138
DHX29	LOC285033	PPP1CC
C2orf60	DNAJC10	GZF1
ASB8	COX15	CCDC121

ZNF20	WHSC1	GMPS
PHRF1	SNRNP48	GRB2
KIAA0247	MUS81	RAB5A
ZNF512	CTDP1	SAFB2
SNX27	MTF2	ERCC8
USP21	TNPO1	ALS2CR4
TTC9C	RBM5	C12orf11
HIP1	ZDHHC21	PPP2R2A
ENOPH1	SCAF1	LOC493754
SLC35E1	POLR3F	ZNF121
FKBP15	USP39	NAB1
CSNK1G1	FBXW8	HELZ
MCM8	SNIP1	KLHL20
DICER1	DBNDD2	UPF2
DHX40	ARMC10	KTN1
TECPR2	DUSP11	WWC2
ZNF451	PDZD8	AMBRA1
WDHD1	MSI2	ATAD2B
UBXN2A	FAM172A	PRKAR2A
USP14	ERCC6	ARHGEF35
XRN2	ANGEL2	FAM59A
THAP1	EIF4A2	IPMK
KDM2A	PIGW	RIOK3
NFYA	KPNA6	IQGAP1
PARP4	CEP250	C15orf57
C20orf117	LLGL2	CDKN2AIP
TTC14	SENP7	INO80D
DOCK7	KIAA0892	ARFGEF2
ABL2	ZXDC	DMTF1
RBM23	FAM120AOS	BAT2L2
POGK	FAM104A	DAZAP2
MAPK9	ANAPC10	BRD7
ZSWIM6	MTAP	ARL6IP5
ZRANB2	MYST4	RTCD1
CREB3L2	OSBPL2	ZNF746
ENTPD4	HNF1B	N4BP1
GNPDA2	PIAS4	CD47
SLC30A5	DNM1L	LDB1
NFX1	EIF1AD	PRDM4
ZNF28	SCMH1	PPIG
UBR2	SETD2	PRPF38B
FOXO3	SYNE2	DIS3
VTA1	MRRF	RAP1B
TTC37	SIPA1L3	C5orf44
C1orf55	FLNB	TMEM188
AHCYL1	LAMA5	INTS4
ASTE1	GMEB2	NPTN
CEBPZ	KIAA1804	B3GALNT2
NR2F2	SUMO1	CASK
NUCKS1	ZEB1	SNX17
TYK2	NAT10	ATF7
AZI2	USP6NL	PGBD3
R3HDM1	LARP7	SLC37A1
SETD2	GRPEL2	RINT1
MYH9	KIAA0368	ERCC4
LOC440354	ARHGAP26	SHQ1
LEPROT	LCLAT1	RAB6A
SIPA1L3	OBFC1	RPS6KA4

MCM9	NCOR1	TGS1
JMJD1C	MED21	MCM4
SEPT8	WARS2	C1orf144
C19orf55	ANKZF1	ATXN2L
ZNF350	HIVEP1	ZNF776
ITSN1	ZNF434	ZNF721
ADAR	LRTOMT	DYNLL2
BMP2K	LOC148189	APAF1
SENP5	IPO8	BCAR1
URB1	EFTUD2	C3orf63
LSG1	ATG9A	GDI2
UTP3	ZNF267	ZFP64
VANGL1	PTMA	DOCK5
SFRS11	CAPZA1	MLH3
HMGN4	ZBTB1	ZNF324B
PJA2	RIT1	DCAF5
UTP14C	CSNK1G3	LIN52
UBE2D3	ZNF133	ASAP1
ASXL1	PAK2	AMMECR1
PCBP2	SGPP1	CWC25
NUP205	HNRNPR	TMED8
RAB18	ANKRD52	SHOC2
MORF4L1	UBE2D3	HAT1
GALNT1	DPY19L3	C5orf22
STT3B	LIMS1	STAM
CSDE1	ZFAND6	BTBD12
AASDHPPT	CALM2	TANC1
PLEKHA1	PIGB	C11orf58
FANCF	TRIP11	NBPF1
IQCB1	YWHAG	CD46
TOP1P1	SUZ12	PHF17
SCAPER	MZF1	NUP188
MYO19	IGBP1	PPM1B
MAPKSP1	FAM117B	PAPSS1
CYLD	ZC3H15	TADA2B
WTAP	GALNT1	C4orf42
HBS1L	ANKMY2	UBN2
AGAP1	AASDH	PAQR3
IRAK4	GABPB1	ZNF264
RAPGEF6	UBAP1	PIAS1
TLK1	NCRNA00094	KIAA0240
ELK4	CDK19	EXOSC10
AVL9	SNX17	PRKAA1
RAB6A	LUZP1	STAT3
TRAK2	CNOT1	C1orf163
TBRG1	TERF2	KCTD9
ZNF823	CHD7	VPS37C
KCTD3	PLEKHB2	RAD51L3
TBC1D23	TNFRSF21	ZBTB44
PASK	CLCN3	GOLT1B
NAPG	POLH	DCP1A
SH3GLB1	DLG5	C9orf25
LRPPRC	AP1G1	PRIM2
ZBTB7A	MOBKL1B	ITGB1
SLC35B3	ATP11A	FNTB
MLH3	SH3GLB1	UBE3B
CDV3	EIF2S3	DNAJC27
ASB7	LOC388152	BZW2

PDE12	ATM	SFRS4
BAHCC1	NAA35	DCAF10
TMEM170A	XPO1	MAP3K7
NFRKB	ZNF777	RAB9A
FBXO30	CDK5R1	CTTNBP2NL
KDM5B	KHSRP	UHRF2
SYNCRIP	METTL4	SLC9A7
MAP2K7	PSEN1	BCOR
BRWD1	UBXN4	SMURF1
FOXO3B	ZNF41	ZNF708
NUP188	SSRP1	WDR41
STX12	ASAP1	C7orf28B
CASC5	SMAD2	COG1
OSBPL8	SPTY2D1	TSN
ANKRD6	MED13	SKAP2
OSBP	DNM2	ZDHHC17
IFNAR1	WWC3	C16orf57
YTHDC2	SHOC2	METAP2
NUP133	RC3H2	ALKBH1
ZSCAN12	GORASP2	PRICKLE3
NFAT5	S100PBP	MYO6
AHCTF1	EIF4H	LETM1
THUMPD3	WASL	ARNTL2
LRCH1	STAM	RFX1
KLHL26	COG2	ADAM9
MCPH1	C17orf75	ISY1
SETD4	MTFMT	STIL
ACTR3	ISG20L2	HCCS
TMEM127	SMYD4	PPME1
CHMP5	BRAP	BAZ1A
FAM188A	AAMP	MIOS
SFRS7	ACIN1	RBM43
RBM8A	IFT81	MTMR1
FBXO45	FCHO2	MAPRE1
POM121	CCNI	CCDC47
DARS	CBLL1	FAM200A
HERC2	IPP	SR140
ZNF79	CAPN1	DCLRE1A
DPF2	TTC3	MTAP
ZNF805	MAGI1	RB1
SLC30A7	AMOTL2	MNT
INPPL1	PTPN23	MANBA
WDTC1	SLC35F5	MLXIP
SCAND2	BTBD1	PHLPP2
SSR1	SMARCA4	LIN54
TIRAP	STAT5B	GGA3
KIF16B	USP33	WDR1
PDPR	SETD4	GOLGA1
SPRED1	DDX20	DDX42
CRYBG3	FUBP3	RNF115
NSUN3	THAP11	MSL2
TXNDC16	SLC35B2	MAPKAPK5
C9orf64	SDHAP1	MED28
ZNF597	PRR14	HEATR3
SNAP23	ARHGEF11	CEP350
ANKFY1	VPS37C	AHCYL2
RAB5C	UVRAG	CHMP7
VPS24	PHF14	PLRG1

LRRC41	CTPS2	ZC3H14
PPP1R15B	ZNF19	RBMXL1
EIF1AX	ZNF326	CWC22
MAP4K3	ZNF250	EEA1
KIAA0776	TMEM106B	STARD7
MUS81	PRKAG1	SENP5
C18orf55	HELQ	SFPQ
LOC100129034	FXC1	TBC1D12
METAP2	SERTAD2	TTC5
ZBED4	PXN	MPZL1
ZNF559	EPS15	SPIN4
C18orf19	NAA15	POLR3E
PDIK1L	SPAST	TOPORS
ZNF333	TNRC6B	TOX4
FRMD8	RAB3GAP2	DHX57
CHTF8	PLSCR1	SECISBP2L
TOP2A	KCTD11	CEP76
SELT	FKBP15	BCDIN3D
SRFBP1	MGC2752	TMEM128
RASAL2	TOPBP1	ZFHX3
ZFP14	PHF3	MTMR3
CEP170	RAB21	TRIM25
MSH6	SIAH1	GOLGA3
WDR19	FIP1L1	TMEM87B
AP3B1	RBBP5	ZNF107
ARGLU1	HNRNPA3	HNRNPD
GFPT1	UBXN2A	FAM175B
MTAP	FAM73A	MORF4L1
RDH14	USP14	ARCN1
THOC2	SGK269	RNASEH1
TWF1	CDK17	AASDH
MARCH8	GCA	NUP153
CASP8	MGC57346	CAMK2D
VPS41	STRN3	GLT25D1
GOLGA1	SLC44A3	GABPB1
HNRNPUL1	METTL6	HEG1
ZNF621	EML4	TAF1A
JARID2	CDK2	MYO19
SCRN3	KIF3A	ZNF286A
ZNF100	RPAP2	USP34
GMPS	SKIL	MPZL3
DKFZP586I1420	C20orf72	TRIP4
POC1B	FAM13B	C14orf4
ERCC5	MAEA	ZNF557
ZNF687	SPAG16	ACTR8
LOC100132707	FGD4	ZNF510
ALS2CR4	RNF145	ROCK2
ZFP36L1	DSC2	SF3A3
ATXN7L3	RBMS2	NFE2L2
ZNF121	TRIM65	TERF2
UNC50	LASS5	ZNF136
MED26	ZFP36L2	VCP
ARHGEF18	MORC3	LOC728024
CASP3	COL4A3BP	AP1S3
PTPN1	PCGF2	CHD2
CAPZA1	LENG8	CEP192
METT5D1	GRB10	RAB11FIP5
STRN4	GNPDA2	ZNF2

PPP1R13B	ANKRD12	ZNF675
GOLGA3	SLC30A5	CWF19L2
METTL2B	SYNJ1	SCOC
CCNDBP1	ZNF800	COPA
ZBTB4	ZNF28	ZBTB2
POT1	PATL1	C3orf34
DAZAP2	USP47	STX7
GSPT1	TMEM39A	WDR60
YTHDF2	SYNGAP1	WDR11
NR2C1	PPP3CA	GTPBP4
RGPD6	EP400	CIC
IKZF5	AHCYL1	HTT
RAB11FIP2	CTNNB1	ZNF777
UBAP1	SLC7A6OS	SGMS2
MAP3K1	NUP35	ORC5L
MLLT1	VKORC1L1	ZNF718
CPEB2	RCHY1	PPIL4
TRIP4	PPP1R9B	UTP18
RRM1	SIRT1	ICMT
UBP1	VCPIP1	NIF3L1
ZNF398	TAF7	WWC3
ATF6B	ZNF202	TIPRL
KIAA1033	SWAP70	B4GALT5
EED	ATP7A	SSH2
COPS4	CASD1	ADAMTS9
DUSP18	CWC27	CNN3
NOTCH2	ZBTB8A	STXBP5
SUPT16H	ZNF629	ZFYVE20
LOC728024	SBF2	PNPLA8
PPP4R1	SEPT8	DCAF16
SETD3	EWSR1	PRRC1
EIF2AK2	SEMA4C	C5orf43
PRPF8	ZNF383	TMEM131
MIS12	CRNKL1	ZNF180
BRAF	CHD9	PXK
GEMIN5	CHD1	PPWD1
RHBDD1	FUS	KIAA0090
ADD3	ARAP1	CCDC41
LARP1	ZNF350	PIK3CB
PARP2	PLEKHM3	QKI
SETDB2	KIAA0232	CXorf23
ZNF295	SPATS2L	TANK
SUFU	NPHP3	AMPD3
CBX5	CEP63	LRRFIP1
FGD6	ADAR	GALNT10
RAB3IP	BRMS1L	HLCS
SPPL3	RAB28	GSR
CCT8	HMGXB3	XRN1
HINT3	MTX2	CANT1
RAD1	SFPQ	SEPT11
NCAPD3	STAG2	SERINC3
CCNK	RAB14	ASB8
ZNF468	ERMP1	C3orf64
ATXN2	STRADA	GOLPH3
C11orf61	PPHLN1	POC5
ZNF514	SAMD1	ANKRD42
C1orf83	CAMK2G	ATN1
DNAJC21	FOSL2	SDCCAG1

LUC7L3	CHM	DNAJB6
WDR48	MSH5	PTAR1
IDE	IQGAP1	THAP2
FAM178A	ZFAND3	PHRF1
ZNF274	RGP1	TSG101
BTBD12	TSR1	RBMX
AFG3L2	PGBD2	FBXO45
TANC1	AP4E1	ARID4B
ZNF644	ITPR3	POM121
DCAF16	PI4KB	GNS
PRRC1	ZNF417	XPOT
DOT1L	LMAN2L	ARHGAP1
LMTK2	ARFGEF2	C2orf43
ZNF329	ABCC1	AMMECR1L
MBD6	GCN1L1	ERC1
TRIM13	METTL8	TRMT5
TMEM131	PLCB3	C17orf63
IFT81	IQCB1	ZNF500
ANKRD49	TAF1A	KIAA0562
CHMP2B	GLTSCR1	ARMCX5
UBE2N	AIMP1	NEK7
VRK2	FRG1B	PDPK1
ZNF480	GSK3A	EIF2C1
DGCR8	MREG	RGPD4
SYNJ2BP	KIAA0528	CSTF2
BLZF1	PTPN2	DENR
PSPC1	FBF1	BFAR
LIN7C	KAT5	BACH1
UGCG	PGBD3	CYB561D1
ZRANB3	LIPT1	SPATA13
ATN1	VPS37B	MKL1
ZNF700	PPP5C	LIG3
SMAD5	DAXX	VHL
ZNF638	CDAN1	CCDC109A
ACTR10	CDC23	TFE3
C12orf4	AKAP8	NCSTN
PRIM2	HARS2	C2orf69
TOR1AIP1	UBE2V1	C15orf44
DMXL1	POLG2	MYSM1
KIAA0391	TRMT61B	PWP1
FAM76A	ANKRD10	CDK2
IGF2R	SOS2	PLEKHA2
RBM15B	SENP6	USP10
ZUFSP	ZBTB39	GATAD2A
SFRS17A	HIVEP2	MASTL
TAF5L	DCUN1D4	MAPK9
CYTSA	PPP2R5E	AGK
RAB8B	MMGT1	RABGAP1
DYM	KIAA1009	MYCBP2
JHDM1D	ZNF320	TXNDC9
MAN2A1	FAM126B	ZNF131
PHACTR4	MED25	MSH6
PNRC2	FTSJD1	C20orf177
SENP2	CCNK	RAD18
HNRNPA3	IFT74	YWHAH
SEMA5A	KDM5B	PCBP1
TTC30A	PPP1R13L	LRRC42
C5orf42	METTL14	SPAG9

GEN1	BMP1	PSMD2
ATXN7L3B	SNAPC3	DOCK1
SNX19	USP28	PHF2
CEP120	PANX1	PPP1R9B
SET	BTBD12	IL17RA
MTF2	METT10D	PIAS4
ZBTB40	ZNF558	C4orf46
MTA2	TAX1BP1	PPM1D
ALKBH1	SYS1	ERI1
SNIP1	ESF1	PKD2
FAM102B	NCBP1	SFRS3
COL4A3BP	LOC90784	LMBRD2
ZNF195	TM2D1	SGTB
C12orf26	FBXO18	GFM1
ATG4C	ANXA4	CNNM4
RELA	GAB1	HNRNPH3
UBQLN1	KIAA0802	PSMD14
UBA5	CDK14	MTPAP
PIGO	PSMD5	PTPRK
DAPK1	R3HDM2	TTLL12
CEP250	SCRN1	CLINT1
KIAA0355	ELF1	TROVE2
NKTR	CCDC14	PIGS
FAM120AOS	FUT11	PRPF38A
TRPM7	C20orf43	SEPT8
NECAP1	KIAA1712	AKAP13
OSBPL2	ASB8	C4orf14
ZBED5	POLA1	ELMO2
EHF	ZNF773	IPO9
ZNF202	DPY19L4	LOC100132707
CMIP	NUP62	FAM161A
CDKN1B	ACTN4	PPID
DDX42	HERC2	GPR157
ZNF546	AGGF1	SPATS2L
ZNF45	PEX26	HEATR5A
FAM83G	HDAC4	PRMT5
ARF3	SLAIN2	TACC1
RALGAPA1	SLC35E1	IPPK
SLC39A6	DDX1	KLC2
SLC10A7	WDR45L	VMA21
CHD1	USP36	NEK9
RAB33B	SFRS12IP1	STX6
CTDSP2	TCOF1	JRKL
PTP4A2	RNF141	TNKS
SFRS6	DSG2	NUP50
FAM199X	IFT80	SFRS2
FAM20B	PDPR	RBM22
IPPK	CHIC2	CASZ1
SCLT1	RAD17	CSNK1G3
PTPRG	LRRC16A	BTG3
UBE2H	HNRNPH2	CCDC88A
RAD50	FBXL14	PGGT1B
TM9SF2	EIF1AX	MRFAP1
TUBGCP5	HBP1	RERE
EXOC8	MKLN1	FAM21A
FBXO5	NCSTN	HTATSF1
PGBD2	NPNT	GATSL1
CDC42SE2	C16orf72	ELK3

CDC5L
ERN1
STXBP3
BTG3
PIGX
USP7
LRBA
ZFAND6
ARFGAP3
SYPL1
WHAMM
ASCC3
MAGI3
MCM3
PDE8A
NAPEPLD
TP53BP1
PTCH1
HNRNPM
ZNFX1
C6orf72
GGA2
ZSWIM1
PTCD3
KLHL7
SH3PXD2A
HMBOX1
SLC37A1
SNW1
STK24
DCTN4
PPP5C
RPRD1A
MCM4
CNNM3
RYBP
ZNF416
SCOC
KIAA1143
TEX261
STX7
DCUN1D4
XPO1
RAB12
ZMIZ1
E2F3
FTSJD1
ZNF740
FAM122A
ZNF33B
TSC1
IARS
CFLAR
ZMPSTE24
ZNF91
WASL
HLTF
TRIM23
ZBED4
C14orf43
PPM1G
BMI1
CNOT3
ZNF473
SLC37A3
KIAA1797
CCDC102A
BCL2L2
MAPK9
CSNK1G2
PEX13
ZSWIM6
FCHSD2
ZNF195
ZC3H18
SRCIN1
PRPF4
C12orf26
PLAGL2
SRFBP1
CIZ1
TCF7L2
PNN
ZNF673
ARHGAP21
WDR19
HNRNPA0
ZNF74
C10orf119
KCMF1
MTMR15
ZNF189
PACSIN2
ZNF639
ZBTB43
ZCCHC7
NHEJ1
MAPKAPK5
PHF6
MAP3K2
RPA1
CELSR1
TBC1D10B
STRN
ERCC5
SNAPC1
UBE2W
ALS2CR4
C14orf79
ANKRD36
TAF8
ACAP2
TBC1D15
LOC253039
C10orf84
NKRF
GALNT1
TTLL5
STT3B
TUG1
METTL8
ZC3H7A
DTX3L
ZNF234
MLLT1
HNRNPM
WTAP
ETNK1
ZNF398
MSL1
DIP2A
CNOT1
SNX25
FBXL20
KAT5
CEP290
PLEKHB2
MPP6
CIRH1A
RNF10
TBC1D23
PRKCI
TRAF3
SENP6
CCDC111
ZNF587
SERBP1
ANLN
ZNF280C
GUSBP1
RAD1
PRMT10
METTL2A
TMED10
GNA12
ARL1
GON4L
ZNF836
RHOA
SUN1
ZNF281
RPRD2
SMYD4
ATP6V1D
MAX
TPM3
UBE2N
YTHDC2
NEO1
WDR44
TNPO2
COG7
ZNF20
SOAT1

KLHL23	FAM135A	HNRNPC
NEU3	LSG1	ZNF174
STXBP5	MAPK14	C12orf51
KPNA1	C2orf24	MCM3AP
C10orf88	ZNF71	PRRG1
ESF1	C17orf42	ZC3H6
FGFR1OP2	PAQR5	FTO
LGR4	DIS3L	HSPA14
TM9SF4	C11orf46	TMEM87A
MDC1	SFRS11	ELAVL1
ZNF160	METTL2B	BNIP3L
STAMBP	RAB35	METTL13
SAV1	SMCHD1	RAB1A
ZADH2	GEMIN4	FBXO38
C5orf24	ZNF862	ZBTB25
UBLCP1	FAM102A	FNIP1
PLEKHA3	ATP9A	CLDND1
SDHAP1	RNF216	SPEN
DENND4A	CCNT1	TAF6
SOAT1	NUP107	PNRC2
ZNF184	KIF13A	RAD21
CCDC142	ATXN1	C11orf9
THAP2	NUP205	TNS3
VPS37C	TMEM184C	RELT
ACVR1B	ANKS3	NRF1
MDN1	HNRNPA1	MAP4K3
HELQ	RAC1	ELF4
ATAD1	C10orf46	HERC3
OTUD3	MAPKAP1	KIAA1429
ZNF432	WDR43	GRK4
ACVR2A	FOXJ2	C18orf19
ITGB1	C10orf76	HOOK1
PSIP1	NPRL3	NMT1
C12orf32	CXXC5	CNOT3
NCOA5	SCAPER	ZBTB40
C2orf43	CBFB	HDLBP
LRRC37B	RNF26	FAM65A
SRP54	C5orf44	MACF1
SLBP	SCARB2	RPL7L1
PDCD10	LOC400027	CCT2
ZNF140	PVRL3	BCL2L2
MTMR2	C15orf40	PCNX
STK35	RAB1B	PITPNA
SMC5	WDR89	ZSWIM6
SUCLA2	XPC	ZNF195
GPBP1L1	TRAF7	CUL1
BTBD9	POLR1A	PGAM5
ADRBK1	KIAA0319L	LATS2
TRIM4	SUPT16H	DBF4B
RBM5	AAAS	SLC35E3
ADSS	AP1S3	SAPS1
HECA	XPO6	MKNK1
MASTL	RAB2B	EP400
FAM190B	ABCA11P	IMMT
LOC729082	KIAA1731	DSTYK
TAF13	ZNF550	ANAPC10
SASS6	CNNM3	PTTG1IP
STIL	ST3GAL5	NAE1

WDR35
ZNF841
PLAGL2
ZNF319
ANGEL2
RNGTT
ZNF673
RIC8A
IPO5
PRKACB
ZXDC
RUNDC1
METAP1
ZBTB24
GTF2H1
TMEM167B
INPP4A
LYRM2
ARHGEF7
ATP13A3
ZWILCH
ZNF138
CRY1
ILF3
PRPF38A
ZSCAN22
TSSK6
ZNF225
SCOC
ZBTB2
WDR75
DCLRE1B
DYRK2
CDC42SE1
ABCE1
ZNF213
TMEM51
PABPC4
KLHL12
ANKRD5
MON2
PRKRA
ZNF718
LRRC37B2
MOBKL2B
TMEM79
ZNF780B
SNURF
IARS
EHMT1
PEX12
RCBTB1
AFF1
TRIM23
DDX47
CHMP4B
LMTK2
SLC35A4
NUP98
TSEN2
KLHDC5
DDR1
METTL3
ZNF799
TPM3
NCDN
ANKLE2
ZMYM5
TCFL5
NCK2
WDR26
NDE1
C6orf89
ETV3
LPCAT4
ZNF160
NUMB
HIP1R
RAB40C
SSH3
RFFL
ZNF689
FLII
RBMS1
RIOK2
CCPG1
PHF15
EIF1AD
VCPIP1
RPL13P5
ZBTB43
ZNF292
NOLC1
SEP15
CENPC1
POC1B
CHD9
DDX50
USP38
KIAA0831
MBTPS2
KIAA0895
ZNF532
SMAD3
DENND5B
MTUS1
KIF3B
PIK3R4
DEDD
ZNF619
NCBP2
RHBDF2
ZFAND3
RARS
DIS3L
STRN4
NPC1
UTP14C
TXNDC12
KIAA1841
ALG10B
C1orf26
TRIM34
YRDC
ATXN1
NUDCD3
ABCC1
MMADHC
POLD3
COIL
TUFT1
GMIP
CSNK1D
UBAP1
IQCB1
MYLIP
C12orf43
ALG10
C6orf72
UTP11L
RAPGEF6
TNRC18

DDX27	TLK1
ZNF8	UNC119B
TTLL1	RHOQ
ZNF318	ATP6AP2
NDNL2	CDC23
ZNF566	TRIM62
CAP1	RBM33
BCL2L13	PDCD7
SFRS7	C1orf52
RBM8A	TDG
E2F6	MON2
GTF2H3	MARS
TMEM168	FGD6
CLCF1	KBTBD7
ZNF420	RAB3IP
MRPL10	SPPL3
BET1L	ARL6IP6
DLGAP4	SNN
PRRG1	NCAPD3
ZNF7	TBC1D8
RBM9	ATXN2
FNTB	FBXO30
EIF3A	MED13
ZNF606	KIAA1967
DCAF10	GORASP2
RAB9A	LIMK1
RNF114	ZNF440
SEC23B	WASL
ARMCX5	ZNF749
SOCS6	TARS
KCTD5	C17orf75
USP9X	CARD6
PRUNE	VPS39
ZKSCAN4	TMEM184B
ARHGAP19	DNAJC6
UBE4A	C14orf135
ANKFY1	METTL9
ZFYVE26	SP100
RAD21	TMEM17
RNF13	ALKBH8
ING3	FCHO2
CDC42BPA	TTC3
KDM2A	ANKRD26
GCFC1	ZMYM5
COG8	FBXO3
UBOX5	FBRS
UBE2O	PLBD2
DDX17	NLK
MAFG	PURA
ADAT1	TMEM43
TMEM50A	LRCH1
FTSJD2	SPRED2
ZBTB40	SESTD1
RPL7L1	ZADH2
PCNX	UGCG
MRFAP1L1	PSMA1
KIF21A	ZNF778
SETD6	SEPT9

LOC151162	BCL2L13
CXorf38	ZNF260
TYW3	ZNF512
PPME1	LYN
CYB5RL	UBE2R2
SMG6	NCAPD2
USP22	WWTR1
MAP2K1	CANX
DAPK1	E2F6
USP1	GTF2IRD1
ZFP14	ZNF592
KIAA0317	DCTD
ITFG2	SUSD1
WDR12	ZNF420
PLEKHM1P	H2AFY
MITD1	FXC1
YWHAH	TMEM69
PCBP1	WDTC1
C10orf28	VPS54
TNRC6A	BUD13
ZNF808	RSL1D1
CEP110	RAB8B
KIAA0226	CAPZA2
MYH9	C14orf169
TAF2	SLBP
NRAS	PPP2CB
ZNF585B	ERLEC1
REV3L	INTS9
CCDC123	NAA30
NEDD1	HNRNPA3
MED23	ZNF33A
GIT1	SUCLA2
DR1	ATP8B1
EIF2AK1	UBXN2A
NT5C2	RWDD4A
NOLC1	AP3M2
ZNF562	GCA
ELMO2	GPATCH2
TOB2	TRMT6
SPIN2B	RBBP6
FPGT	GPBP1L1
DCTN1	XIAP
KIAA0652	TAOK2
E2F4	GK5
TBCCD1	ACLY
CRLF3	COG8
SLC25A24	MUS81
RBM4B	METTL6
IFT172	LOC100129034
SKI	TMEM167A
MBTPS2	C16orf72
ZNF532	NSMAF
FAM168A	ADSS
VMA21	TTC4
RCN2	PMS2CL
TBC1D12	STX17
CMTM1	IL6ST
CLK2	CIZ1

CCDC76	PIGO
LLGL1	CNOT6L
RAPGEF1	UBE2G1
MAP3K8	TMEM39A
UNK	LOC550112
EXOC8	C17orf80
MTCH1	FAM120AOS
SLC35C2	PTPRF
DEDD	RDH14
FIGNL1	ENTPD1
COPS8	UBFD1
SDF2	PPPDE1
VANGL1	EDC3
PAIP1	REV3L
C15orf52	SIPA1L3
DNAJC5	HNRNPUL1
USP19	UBE2D1
C9orf102	NIN
PPL	GNPTAB
ZNF107	UBE2E1
UBE2Q2	SPATA5
GNA13	PLXNA1
PPP3CB	HS2ST1
YTHDF2	POLG
CD47	EHBP1L1
LETMD1	ALG9
SLC30A9	CGRRF1
SDCCAG8	SLC25A24
ILF2	URGCP
CAMK2D	CLIC4
SEMA4B	OBFC1
LDOC1L	ATXN7L3
CPEB2	BMPR1A
MYO19	C10orf84
MSH2	NEK4
C14orf4	FYCO1
GIT2	RAB14
HIPK1	VIPAR
RPS6KB1	LLGL1
FAM38B	KIAA1524
IGSF3	FBXL19
SRPK1	PPP1R2
TIAL1	PTPN21
EIF2AK2	NGLY1
C5orf51	DZIP3
RAB11FIP5	C7orf25
LOC729678	FAM48A
NARS	CCDC112
AAK1	ITPR3
PKD1	CDK11A
KIAA1522	PALLD
DCBLD2	ERLIN2
KIAA1530	ERO1L
BCAR1	LSM12
C1orf52	ARL2BP
SH3BP5L	PJA2
MRPL3	ZC3H12C
ZNF490	FAM91A1

H3F3B	NACC1
VTI1A	PITPNM1
BRCC3	MAST2
ZNF468	BBS10
PRKDC	NR3C1
POFUT2	NAPEPLD
SMCR7	NIPAL2
APEX1	ANXA5
CDC27	SCAMP1
ZNF664	TMEM30A
LOC100132247	GSK3A
C1orf124	PRKCA
REPIN1	KIAA0528
C6orf134	TIAL1
UGGT1	AGTPBP1
HPS6	DUS4L
ZNF274	PHAX
PRKAR1A	ATP5A1
STAG3L3	HAUS1
CLTC	WDR33
SNAPIN	RAB2B
C2orf29	UBE2A
ZNF180	TTC33
THUMPD2	ABLIM1
ALKBH8	LAPTM4A
UBE3C	KATNAL1
FRMD4B	RBM19
XPO5	MYO18A
SCYL3	CASP7
KDM6A	UTP23
HDHD2	LASP1
UNKL	DFFA
GALNT10	XPO1
NFE2L3	KHSRP
TRIM11	PLEKHM2
ZNF585A	DNAJC24
DET1	STK38
CDC42EP4	KIAA1704
LRCH1	FAM126B
ACP1	YIPF6
STAG3L2	C1orf9
DEK	KDM5B
PSPC1	METTL14
MCPH1	ZNF268
SERINC3	EIF2C2
COQ7	IFIH1
FAM192A	ELP2
FLJ10357	ANXA7
NCEH1	AFF1
FAM188A	TRIM23
ZNF518A	MLL
DENND4A	C5orf15
LOC678655	BTRC
PHRF1	DHX30
AP3M1	NXF1
ZNF700	TES
SLC38A1	SNTB2
C9orf80	SMCR7L

DCTD	DOCK9
SPRYD3	HIF1AN
POM121	IPO11
ZNF200	DLD
RXRB	C2orf44
NUP155	EIF4A3
LOC284441	NFAT5
HIPK3	MAST4
INPPL1	HIP1R
UXS1	FECH
SFRS4	ZMIZ2
ZNF772	FUBP3
WDR90	MRPL19
TOP2B	CS
PCSK7	ZNF318
UBE2E3	NDNL2
KIAA1407	DDX19A
MPHOSPH8	WWP2
TULP4	PMS2
PFKFB3	ATAD1
NSUN3	PIP4K2B
PHLPP1	NOMO1
ARPC2	UBQLN2
ZNF140	C4orf43
NBEAL2	CASP8AP2
TAF6	INPPL1
SFRS15	FAM76A
SMURF1	POP1
ZNF302	SSR1
ANKRD36BP1	BRD2
ZCCHC8	HINFP
SPATA13	IGF2R
NAA25	TBC1D14
C11orf9	CENPO
C5orf42	TULP4
STAU2	DCTN6
AP3M2	SFRS15
DSTN	HNRPDL
SFRS14	YAP1
FRS2	C5orf42
C17orf85	C12orf66
CCDC109A	PPP1R15B
PARP4	CHMP1B
C4orf10	STRN3
ADD1	SETBP1
TRIP10	ADRBK1
KIAA1429	KIAA0513
PACS1	PDP2
LOC100129034	UBE2O
C20orf117	ZBED4
TTC14	MAFG
NMT1	TAB3
RAB10	KIAA1432
TSTD2	ZNF473
GMEB1	FAM105B
MTA2	FAM40A
ITGB4	PTGES3
YTHDF3	INTS8

RNF34	MORC3
BCL9	PEX13
PTGES3	TTF1
IFFO2	SFRS12
SYNGR2	ERCC6
INSIG2	UBA5
GCC1	RASAL2
KIAA0907	TMX2
NDST2	TCP11L2
TXNDC9	ZNF28
EFTUD1	PIGW
TCP11L2	DCTN2
GTF3C1	KPNA3
NOTCH1	YTHDF1
HERPUD2	RHEB
SPPL2B	C1orf55
DRAM2	MARK3
NFATC3	SPOP
GTF2H1	PIP4K2A
BAT1	STX18
R3HDM1	CEP110
LIN54	MPP5
THOC2	DYNC2LI1
THBS3	NCOA2
ZNF707	AZI2
SRGAP2	GYG1
CRIM1	VGLL4
ZNF546	PGK1
FAM149B1	SNX4
CLASP2	NCAPG2
GZF1	SRGAP2
RALA	FAM53C
ABCC10	ILF3
SCRN3	TXLNA
HGSNAT	FUT10
NDRG3	CLASP2
LOC645676	CPNE8
EIF2C4	GIT1
APBB2	RBM47
ZDHHC6	POMT2
FUT4	SNRNP40
AGPS	KIAA1310
SEPHS1	PNMA1
ZNF614	CHD1
C9orf30	CADPS2
SOCS4	SEPHS1
TMEM132A	KIAA0652
DDX50	CALCOCO2
ZFYVE9	ACER3
ZNRF3	EXOC4
PANK2	LCLAT1
TUBGCP5	NCOR1
BMP2K	TOMM70A
SENP5	FBXL3
GTF2E1	NKRF
SLC26A11	PELI1
FAM200B	PPT1
KCTD20	TLR3

PIK3R4	FTSJ2
GDAP2	WDSUB1
MARCH6	PIGX
MRPL49	RBM15
METT5D1	AMZ2
TMEM2	PCGF3
WNK1	POLE3
PPP1R13B	USP7
KIAA2018	RACGAP1
STARD13	USP4
SLC35A5	ZNF417
KIAA0586	DYRK3
FAM22D	NUPL2
PIGK	GLS
TTC27	TRAFD1
LSM14B	RANBP9
TRIM34	DPYSL2
IKBKB	SDCCAG8
MAPK8	CPSF7
SYPL1	RALGAPA2
DCAKD	CHST14
RNF11	TADA2A
PGS1	TFG
MCM3	PARP1
PLEKHA1	TPM4
NAPEPLD	ZNF674
PER2	HECTD3
C11orf30	ADCY6
FAM60A	RRM1
TOP1P1	RAVER2
GNAQ	CRK
UBP1	CDK19
B3GALNT2	UBP1
ETV6	GGA2
UBE2D2	ZNF192
ZNF192	RPS6KB1
CORO2A	ANAPC4
EXOC2	SRPK1
HP1BP3	C4orf29
KCTD13	FAM18B
LOC220729	AHR
AVL9	CCT4
STK24	KIAA0753
DCTN4	TRMT2B
WDR33	SETD3
SNAPC5	GPBP1
SGSH	STK10
TAF1C	C16orf63
ANXA2P1	DYNC1LI1
WRNIP1	FASTKD5
ZNF823	BBS4
CALCOCO1	KTI12
KIAA0467	ZNF567
RHBDD1	ZNF225
IQCK	PTPRE
PLCG1	C18orf8
TSGA14	CRYZL1
PRKCI	DCBLD2

ZNF175	KIAA1530
SUPT5H	SOS2
ZNF641	MARCH5
HIRA	DBF4
TSNAX	PLS1
TNS1	RUVBL1
GART	TNFAIP1
DHFRL1	SUFU
DAB2IP	TOR1AIP2
SPATA20	SLFN5
RAB12	PRKRA
GPD2	BBS1
DDX58	TEX2
ANKRD27	ZNF468
ZNF613	BCAS2
SERBP1	SNURF
DAAM1	TMEM38B
ZNF37B	RCBTB1
ZNF280C	ASB3
GOLGA6L10	ZNF527
ANXA2P2	LITAF
MOGS	SIRPB1
NCAPD3	KPNA1
SNX13	FMNL3
STK3	TRIM13
MAVS	CYTH3
ZNF322A	GXYLT1
KIAA1967	NMD3
OTUB1	REXO2
PPIP5K2	PPCS
FAM118B	PWP2
TRAK1	KDM6A
GOLPH3L	KIN
PTPN11	NUP133
TAF1B	ZBTB41
DHX30	AHCTF1
SUN1	DNASE1L1
ZNF410	FBXL17
GATAD1	SLC26A2
KIAA2026	NUMB
SH2D3A	SH3RF1
PPM1B	RFFL
ANKRD50	UBAP2
SPINT1	SSR3
TMEM17	TMEM214
IPO11	VPS33B
RNF40	TCEB3
AACS	DKC1
TERF2IP	SMC2
ACBD3	MTA3
CETN3	PAFAH2
ANKRD49	SFRS7
LRRFIP1	DTWD1
MAGEF1	NUP62
PDCD6	ZNF200
DHX38	VPS33A
ZFYVE16	NDUFS1
SPRED2	ASCC1

MMS19	CYB5R4
POC5	ZFP90
CDR2	C7orf28A
CLCN2	KIAA0182
RRAGA	LRRC37B
SEPT9	ZDHHC13
CCDC127	RNF24
TRAPPC10	ABCA2
ZNF174	USP12
APH1A	RBM15B
NCAPD2	MCM8
MCM3AP	FNBP1
WWP2	RC3H1
FAM21C	PHF3
PMS2L4	HAUS6
AP3S2	NECAP2
RIOK1	DSG2
ZNF484	LOC651250
ATAD1	SENP2
LOC642826	EDC4
UBQLN2	WDHD1
PRIM2	RAD17
TMEM69	WDR55
MOBKL2A	LRRC16A
SLC30A7	AMD1
UQCC	GPSM2
VPS35	ATXN10
C2orf64	RBM17
PLD2	UFM1
FTO	FBXL14
ZDHHC13	MGC57346
RNF160	CLK3
RGNEF	BTBD9
MAP3K7	ARHGAP29
RG9MTD2	TMEM50A
RUFY3	TTC14
TBC1D14	ACTN1
EXOC5	YTHDF3
MED29	MBTPS1
DVL2	SNX10
FNIP1	C9orf6
MYO5B	NRBP1
ATF7IP	MRFAP1L1
MRPL44	UACA
CYP20A1	PTER
ZNF708	SMEK1
ZNF446	AMIGO1
ABCF2	SACM1L
GMFB	FAM172A
TRMT6	USP22
ACLY	OLA1
GTPBP2	VTA1
ZDHHC7	KCTD1
SNX5	ALS2
TWISTNB	FAM111A
METAP2	PPP2R5B
OGFOD1	ALS2CR8
ADSS	UBE2MP1

CPSF2
LZTR1
MFSD6
CCT2
PIK3C2A
NRBP1
PMS2CL
GPR176
MDM1
SEH1L
STX17
DBF4B
SLC35E3
ZC3H10
DDX3X
CSTF1
C13orf23
MRPL45
C1orf55
DYNC1H1
YPEL5
LRRC42
DND1
ITGA6
SF3B2
ZNF223
KIAA0556
ECT2
DUSP12
NUCKS1
ZNF691
MPP5
MANBA
INPP4A
C7orf64
MANBAL
ZNF197
JAG1
C1orf25
GOLGA1
SNX4
JOSD1
HSDL1
RANBP3
ZNF45
CLINT1
ZBTB9
TROVE2
RCE1
TMEM194A
CUL2
DKFZP586I1420
GATC
ZBED5
ZBTB34
ZNF202
RAP1GAP
MARCH8
CLP1
KIAA0174
SMC4
C1orf25
ZNF585B
ZNF621
EPB41
PRKRIR
LEPROTL1
TRIM8
PDCL3
TAOK3
PAWR
C1orf107
OGFRL1
HCG18
MED15
KDM2B
RCN2
ARHGAP17
ARMC1
PDE6D
FAM135A
CD58
EXOC8
RAP2B
POLR2D
ZNF461
ATP6V1B2
ZNF642
DPH2
LRRC40
MRPL49
NOMO2
TGFBR2
KPNA2
ZNF25
PHF16
DGKD
PGAM1
C1orf103
ACOT9
CALM2
ZFX
HN1L
GPRIN1
SPAG1
WHAMM
RAC1
PLCB3
GUCY1B3
CHUK

TAF4
LDOC1L
ZNF618
TOP1P1
NIP7
CKAP2
TNIP2
VAMP3
HBS1L
RPF1
TBC1D9
UBE2D2
SEC22A
ATF6B
PTCD3
MAPKAPK2
LHFPL2
EED
MFF
MYO5C
PTPN2
TRAF7
AKAP2
KIAA0319L
MAPK7
IL10RB
EIF4ENIF1
NT5C3
RNH1
SPIN3
MIS12
ANXA2P1
TYW1
POLG2
UBE2I
ARIH2
SLC4A7
WSB2
DAP
ZNF613
GLTP
SLC33A1
FBXO42
ANXA2P2
STK3
TMEM5
IFT74
ZNF548
CENPE
H2AFV
EXOC7
TAF15
GRIPAP1
LOC647979
LIMA1
BZW1
ZFP30
LOC400657

ZNF558
NCAPH
ST3GAL2
RMI1
RNF220
LNX2
ZC3H11A
TM7SF3
GORASP1
XPO5
PARN
PPP1R3D
NCDN
ANKLE2
RDX
NCK2
WDR26
C1orf183
BRI3BP
FANCM
ZFYVE16
KLHL26
KIAA1712
TUBGCP3
NBEAL1
ZNF248
LNPEP
FAM188A
SEC31A
CREBL2
TPP1
CCDC82
LRRC59
FAM21C
BET1L
MFSD11
SLC6A6
ZNF805
TOR1AIP1
LRRC37A4
MCFD2
ZNF786
SRP54
LRRC8B
KTELC1
AGBL5
ETV1
B3GNT2
C9orf85
UBE2E3
FEM1B
KIF16B
PBRM1
TFRC
WHSC2
SIAH1
RNF13
C7orf44

UBA1
SPTBN1
PQLC3
ZNF84
STXBP4
C20orf117
CAMTA2
WDR73
PARVA
LRP10
ZRANB2
BRD1
BBS2
FBXO21
TMEM65
LOC729082
KLF7
NME7
C12orf26
FAM173B
TBC1D25
MYCBP
MCM6
KIAA0649
UBR2
NOTCH1
AHDC1
HERPUD2
ZNF673
UTRN
WDR19
RG9MTD3
NR2F2
RFC1
STIP1
UTP6
ZWILCH
PIP5K1A
FAM83G
SBF2
KIAA1826
GTDC1
GOLIM4
TMEM194A
NT5C2
CCT5
ZNF828
CLUAP1
MUTED
SLC10A7
TOB2
SNAPC1
ASNSD1
CENPJ
ZFP36L1
SKI
ABCC5
PTPRG

PSMD10
NONO
TBC1D8B
DFFB
PSMC6
SAMD1
MRPL35
CAMK2G
KIAA1737
PTMA
FIGNL1
METT5D1
FAM115A
CRKL
MESDC2
KIAA1949
KIAA2018
TTC21B
NEDD4L
RUFY1
COMMD8
PPP3CB
WDR53
PPIC
PGS1
NUP214
MAPKAP1
PRPF19
SMAD7
DENND4B
FNBP4
CMPK1
ZNF443
PARD3
ANO6
CCDC115
LOC441294
CHORDC1
SUPT16H
CDC40
SLC22A5
C2orf67
BAHD1
MECOM
RBM41
ZNF416
TNIK
PKD1
KIAA1522
ATP11A
KIAA1143
WDR75
NAA16
PHC2
ZNF259
SNX7
ZNF354B
DCUN1D4

MUL1
ENDOD1
COMMD10
PHF13
ZAK
MTRR
LRRC37B2
RANBP6
ASAP2
NFRKB
CEP68
TEP1
TSC1
SLC25A17
EHD4
PTPLB
ZBTB17
PRKAR1A
GOPC
ZC3H7B
EIF2A
PRPF18
KLHDC5
KLHDC10
RNF40
FAM114A1
OSBPL8
PRDM2
ANKRD6
OSBP
RSU1
MAP2K4
ITGA2
TCTN3
PTPN23
PDCD6
TMEM50B
HCFC2
ZNF160
ACP1
DEK
C5orf24
FBXO22
ZRANB3
RCC2
TMED2
MRPS30
ELK1
USP21
ACVR1B
RBM8A
UCHL5
TMEM168
GARS
CCDC46
CASP6
ATG2B
MAFK

ACVR2A
GANAB
MOBKL2A
EAF1
ARHGAP12
FAM107B
XYLT2
INTS5
USP40
BUB3
SCAND2
PLSCR1
KCTD11
USP36
ZUFSP
TAF5L
SMPD4
FARSB
KRAS
OCIAD1
HSP90AA1
OGT
CYP20A1
NUMA1
CCDC97
COX15
RAD23B
SNX19
KDM2A
IDS
LOC283267
YBX1
MTF2
EIF2B1
STT3A
FBXW8
LRRC37A2
KIAA1217
GPR176
TGFBR1
POMGNT1
FAM190B
FTSJ3
ARHGAP18
SASS6
TMCO4
EFTUD1
RABL3
SAE1
RNF19A
CSRNP2
MUDENG
TMEM33
HPS4
ASTE1
DND1
NOL11
ZBTB24

NUP35
TYK2
LYSMD3
COL4A1
USP5
ZBTB3
SLC39A1
C7orf64
TINF2
ZNF45
ZBTB9
MTRF1L
EWSR1
LLPH
TBC1D10B
DKFZP586I1420
SEMA4C
FAM49B
